# Exploring the mechanism of Taohong Siwu Decoction on the treatment of blood deficiency and blood stasis syndrome by gut microbiota combined with metabolomics

**DOI:** 10.1186/s13020-023-00734-8

**Published:** 2023-04-23

**Authors:** Yao He, Huajuan Jiang, Kequn Du, Shengju Wang, Minmin Li, Chuan Ma, Fang Liu, Yan Dong, Chaomei Fu

**Affiliations:** 1 State Key Laboratory of Southwestern Chinese Medicine Resources, Pharmacy College, Chengdu University of Traditional Chinese Medicine, 1166 Liutai Avenue, Wenjiang District, Chengdu, 611137 Sichuan China; 2 Guizhou Yibai Pharmaceutical Co., Ltd, 550008 Guiyang, China; 3 The Affiliated Hospital of Chengdu University of Traditional Chinese Medicine, 39 Shierqiao Road, Jinniu District, Chengdu, 610032 Sichuan China

**Keywords:** Taohong Siwu decoction, Blood deficiency and blood stasis syndrome, Metabolomics, Gut microbiota, Platelets, Amino acid metabolism

## Abstract

**Background:**

Taohong Siwu Decoction (THSWD) is a prescription which included in the “List of Ancient Classic Prescriptions (First Batch)” issued by the National Administration of Traditional Chinese Medicine (TCM) and the National Medical Products Administration of the People’s Republic of China. THSWD is effective and widely applied clinically for many diseases caused by blood deficiency and stasis syndrome in TCM, such as primary dysmenorrhea, menopausal syndrome, coronary heart disease, angina pectoris, and diabetes.

**Methods:**

The TCM model of blood deficiency and blood stasis syndrome was prepared by ice water bath combined with cyclophosphamide, and the rats were randomly divided into control group, blood deficiency, and blood stasis model group, positive group, and THSWD treatment group. Pharmacodynamics measured the blood routine, blood coagulation, and other related indexes in rats. UHPLC-MS technology was used to analyze the changes in the fingerprints of metabolites in the plasma of rats with blood deficiency and blood stasis syndrome, and combined with mass spectrometry information and public database retrieval, to find potential biomarkers for screening metabolites. At the same time, 16S rDNA sequencing technology was used to identify intestinal flora, and statistical analysis was used to find differences in strain diversity between groups.

**Results:**

THSWD administration can significantly improve the physical signs, blood routine, and hematopoietic factors caused by the blood deficiency and blood stasis syndrome model, and improve the symptoms of blood deficiency. The results of the general pharmacological studies showed THSWD groups improved changes in blood plasma viscosity and coagulation-related factors caused by modeling, and improved coagulation function significantly. The metabolomic analysis found that compared to the model group, THSWD exerted better effects on β-alanine, taurine, l-tyrosine, l-arginine, Eugenol, sodium deoxycholate, and deethylatrazine. Twenty-three potential differential metabolites showed intervention effects, mainly involved in eight metabolic pathways, including amino acid metabolism, taurine and hypotaurine metabolism, vitamin metabolism, and nucleotide metabolism. Gut microbiota data showed that, compared to the control group, the relative abundance and value of Firmicutes and Bacteroidota of the blood deficiency and blood stasis model group was significantly reduced, while the relative abundance of Actinobacteria, Spirochaetota, Proteobacteria, Campilobacterota, and other pathogenic bacteria was significantly increased. Following THSWD intervention, the abundance of beneficial bacteria increased, and the abundance of pathogenic bacteria decreased. Correlation analysis between the gut microbiota and differential metabolites showed that the two are closely related. THSWD affected the host blood system through mutual adjustment of these two factors, and improved blood deficiency and blood stasis syndrome in rats.

**Conclusion:**

The blood deficiency and blood stasis syndrome model of TCM disease caused by ice bath combined with cyclophosphamide lead to changes in the pharmacology, metabolomics, and gut microbiota. The intervention of THSWD can improve the symptoms caused by blood deficiency and blood stasis. The mechanism is mainly through the regulation of platelet function and amino acid metabolism.

**Supplementary Information:**

The online version contains supplementary material available at 10.1186/s13020-023-00734-8.

## Background

Taohong Siwu decoction (THSWD) is a well-known prescription for blood deficiency and blood stasis syndrome in Traditional Chinese Medicine (TCM). THSWD is widely used in gynecological diseases caused by blood deficiency and stasis traditionally, such as irregular menstruation, menstrual blood block, menstrual sticky, abdominal pain, and abdominal distension. Based on its function in blood deficiency and blood stasis syndrome, THSWD is also widely used clinically for fractures, postoperative thrombosis, coronary heart disease, angina pectoris, breast cancer, diabetes, and complications [1–4]. THSWD was first included in the “Yi zong jin jian - Gynecological Treatment Principles” in the Qing Dynasty (1636–1912), and consists of six botanical drugs, including *Rehmanniae Radix*, *Angelicae Sinensis Radix*, *Chuanxiong Rhizoma*, *Paeoniae Radix Alba*, *Persicae Semen*, and *Carthami Flos*. At present, THSWD is a prescription documented in the “List of Ancient Classic Prescriptions (First Batch)” issued by the National Administration of TCM and the National Medical Products Administration of the People’s Republic of China.

THSWD is a typical clinical prescription that can be used to treat different diseases with the same therapeutic principles. In the TCM system, the same syndrome may cause different diseases, which can be treated with the same therapeutic principles using a single formula. Blood deficiency syndrome describes a condition in which there is insufficient blood in the human body to fully nourish the viscera, meridians, and the body as a whole. Diseases are common in anemia, arrhythmia, hypertension, neuropathic headache, etc. [5, 6]. Blood stasis syndrome is one of the most common clinical syndromes and a common syndrome of many complicated major cardiovascular and cerebrovascular diseases, including hypertension, coronary heart disease, and cerebral infarction [7–9]. Blood deficiency and stasis affect each other and are closely related. Blood deficiency can cause blood stasis, and long-term blood stasis aggravates blood deficiency. Clinically, blood deficiency and stasis often occur simultaneously in women and are common clinical symptoms and syndromes in many diseases. However, the underlying mechanisms have not yet been elucidated. We have long been engaged in the basic research and preparation development of THSWD and found that most of the literature reports are aimed at studying the pharmacological mechanism of a certain disease in isolation. There has been almost no research on the pathogenesis of blood deficiency and blood stasis syndrome and the treatment mechanism of THSWD.

THSWD has been proven to be effective in treating blood deficiency and blood stasis syndrome, but its multicomponent, multi-target, and multipathway therapeutic mechanisms are still unclear. Metabolomics is a holistic and dynamic study of the influence of exogenous TCM on the metabolic state of the body. This method coincides with the overall view of TCM and has shown great potential in clarifying the basic treatment mechanism of TCM, as well as disease-related metabolites and metabolic pathways [10–12]. The intestinal microbiota is both a participant and a regulator of metabolic processes [13–15]. In recent years, an increasing number of studies have shown that alterations in the host metabolic phenotype and imbalances in the gut microflora are closely related and play a key role in the development and progression of the disease [16]. The metabolism of a host is regulated not only by its genome but also indirectly by the commensal flora. Metabolomics can effectively screen for biomarkers of host health or disease, while most TCM decoctions are taken orally and can interact directly or indirectly with the gut microbiota. The combination of metabolomics and microbiology techniques provides a powerful tool to further study the relationship between host metabolism and gut microbiota and helps to reveal the mechanism of action of TCM decoction in treating diseases [17–23].

This study thoroughly analyzed the efficacy and mechanism of THSWD in treating blood deficiency and blood stasis syndrome. To this end, an ice-water bath combined with cyclophosphamide was used to prepare a blood deficiency and blood stasis syndrome model. UHPLC-MS technology was used to analyze the metabolites in rat plasma. Through mass spectrometry and public database retrieval, the detected metabolites were identified, the differences in metabolites between each group were screened, and their metabolic pathways were analyzed. Using microbial 16S rDNA high-throughput sequencing technology, we further studied the changes in the structure and composition of the gut microbiota of rats after THSWD intervention and identified the bacterial genera with significantly different abundances. Finally, the metabolites and gut microbiota were comprehensively analyzed, and the mechanism of THSWD in the treatment of blood deficiency and blood stasis syndrome was explained from the perspective of plasma metabolites and intestinal microecology. Our results lay the foundation for the clinical application of THSWD in the treatment of blood deficiency and blood stasis syndrome.

## Materials and methods

### Botanical drugs and preparation of THSWD extraction

The six botanical drugs in THSWD were all purchased from Sichuan Neautus Traditional Chinese Medicine CO., LTD. *Carthami Flos* produced in Xinjiang (China) is the dried flower of *Carthamus tinctorius* L., *Persicae Semen* produced in Beijing (China) is the dried mature seed of *Prunus persica* (L.) Batsch, *Rehmanniae Radix* produced in Henan (China) is the dried root of the Scrophulariaceae plant *Rehmannia glutinosa* Libosch., Paeoniae Radix Alba produced in Anhui (China) is the dried root of *Paeonia lactiflora* Pall., *Chuanxiong Rhizoma* produced in Sichuan (China) is the dried rhizome of *Ligusticum chuanxiong* Hort., *Angelicae Sinensis Radix* produced in Gansu (China) is the dried root of *Angelica sinensis* (Oliv.) Diels. The above-mentioned Chinese medicines all comply with the relevant regulations of the 2020 Chinese Pharmacopoeia. The prescription was based on the “List of Ancient Classic Prescriptions (First Batch)”, 11.19 g *Rehmanniae Radix*, 14.92 g *Angelicae Sinensis Radix*, 5.6 g white *Paeoniae Radix Alba*, *Chuanxiong Rhizoma* 3.73 g, *Carthami Flos* 3.73 g, and *Persicae Semen* 3.78 g. The medicinal materials were soaked in 8 times the amount of water for 30 min, decocted for 1 h for the first time, decocted for 1.5 h for the second time, combined the two decoctions, concentrated under reduced pressure, the concentrated liquid was fixed to 43 mL (1 g crude drug amount/mL) to obtain THSWD.

### Chemical composition analysis and quality control of THSWD

THSWD was diluted 10 times with double-distilled water. The reference substances of gallic acid, ferulic acid, paeoniflorin, hydroxysafflor yellow A, amygdalin, and ligustilide were accurately weighed and dissolved in methanol to make the mass concentrations respectively 0.2037, 0.02625, 0.3159, 0.0565, 0.1287, 0.5000 mg/mL single reference solution. And the single reference solution was stored at − 20 ℃.

Samples were filtered with a 0.22 μm membrane before UHPLC-MS (Thermo Fisher, United States) analysis. Analytes were separated using a SunFire-C18 column (3.0 mm×150 mm, 3.5 μm) with a column temperature of 25℃ and a flow rate of 0.2 mL/min. Mobile phase B was acetonitrile, and the mobile phase A was water containing 0.5% formic acid. A gradient elution program was applied (0–10 min, 5–7% B; 10–35 min, 17–22% B; 35–40 min, 22–65% B; 40–45 min, 65–80% B; 45–50 min, 80% B). The detection wavelengths are, respectively, 210, 230, 328, and 403 nm.

### Animal and experimental design

Female SPF SD rats (180 ± 20 g) were purchased from the Laboratory Animal Research Institute of Chongqing Academy of Chinese Materia Medica (Chongqing, China), animal license number: SCXK (Chongqing) 2017-0003. The rats were kept in a temperature-controlled room at 25 ± 2 ℃. During the experiment, standard feed and water were provided. The study was reviewed and approved by the Animal Ethics Committee of Chengdu University of TCM (2014DL-023). After 1 week of adaptive feeding, they were randomly divided into a control group, model group, positive group, THSWD low (THSWD-L) group, THSWD medium (THSWD-M) group, THSWD high (THSWD-H) group with 8 animals in each group. Rats in the model group, positive group, and THSWD group were immersed in ice water at 0–1 ℃ for 20 min for 14 consecutive days, and 40 mg/kg cyclophosphamide was injected intraperitoneally from the 11th day for 4 consecutive days. Rats in the THSWD-L group, THSWD-M group, and THSWD-H group were orally administered THSWD at dosages of 5 g/kg, 10 g/kg and 20 g/kg (equal to the clinical dose, calculated by the weight of the crude drug), the positive group was given Aifu Nuangong Pills by gavage every day (by pill weight: 1 g/kg), once in the morning and evening, for 14 consecutive days. The control group and model group were given an equal volume of normal saline.

### Observation of rat signs

Observe the physique, coat color, paw color, mental state, and activity of rats in each group. After blood collection, the spleens were dissected to remove the excess fat tissue, and the analytical balance was quickly used to accurately weigh. Next, we calculated the organ index [organ index = organ mass (mg)/body weight (g)], and compared the spleen index of rats in each group. After the rat spleen was weighed, it was stored in polyformaldehyde for use in HE-stained sections.

### Detection of related indexes of blood deficiency in rats

0.5 mL of heparin sodium anticoagulated whole blood from the abdominal aorta of the rat was taken, and the blood routine, white blood cell count (WBC), platelet (PLT), red blood cells (erythrocytes), and hemoglobin (HGB) of the rat were detected by an automatic blood cell analyzer. 1.5 mL blood was drawn from the abdominal aorta and left for 2 h. After serum separation, centrifugation. Use a pipette to aspirate the serum into an EP tube and store it in a refrigerator at − 80 ℃ for testing. ELISA kit was used to detect granulocyte-macrophage colony stimulating factor (GM-CSF), macrophage colony stimulating factor (M-CSF), interleukin-3 (IL-3), and interleukin-6 (IL-6) in rat serum.

### Detection of blood stasis related indexes in rats

A part of the whole blood in the EDTA anticoagulation tube was taken out, the whole blood viscosity was measured, and the remaining supernatant was centrifuged to measure the plasma viscosity. Take 1 mL of heparin sodium anticoagulant blood for the determination of whole blood viscosity. The shearing parameters were set to five grades: 1 S^− 1^, 5 S^− 1^, 50 S^− 1^, 100 S^− 1^, and 200 S^− 1^, and the blood viscosity meter was used to measure the whole blood viscosity. One mL of heparin sodium anticoagulated blood was centrifuged at 3000 r/min, and 0.25 mL was used for plasma viscosity determination. The centrifuged plasma was taken, and the thrombin time (TT), prothrombin time (PT), activated partial thromboplastin time (APTT), and fibrinogen content (FIB) were determined with kit.

### UHPLC-MS-based metabolomics

#### Plasma sample preparation

Blood was collected from the abdominal aorta of rats in the control group, model group, and THSWD-M group, anticoagulated with EDTA, and the plasma was collected by centrifugation and stored at − 80 °C for plasma metabonomic determination. All plasma samples were thawed at 4 °C, 100 µL of each sample was placed in a 2 mL centrifuge tube, and 400 µL of methanol was added to each centrifuge tube, shaken for 60 s, and mixed. The samples were centrifuged at 12,000 rpm at 4 ℃ for 10 min, and all supernatants were taken, transferred to a new 2 mL centrifuge tube, concentrated, and dried in vacuo. 150 µL of 2-chlorophenyl alanine (4 ppm) 80% methanol solution was reconstituted, and the supernatant was filtered with a 0.22 μm filter to obtain the sample to be tested. Take 20 µL of each sample to be tested and mix them into a QC sample, which is used to correct the deviation of the mixed sample analysis results and the error caused by the analytical instrument itself.

Samples were analyzed by UHPLC-MS (Thermo Fisher, United States). Analytes were separated using an ACQUITY UPLC® HSS T3 1.8 μm (2.1 × 150 mm) with a column temperature of 40℃ and a flow rate of 0.25 mL/min. Mobile phase A was acetonitrile, and mobile phase B was water containing 0.1% formic acid. A gradient elution program was applied (0–1 min, 2% A; 1–9 min, 2–50% A; 9–12 min, 50–98% A; 12-13.5 min, 98% A; 13.5–14 min, 98 − 2% A; 14–20 min, 2% A). The positive ion spray voltage was 3.50 kV, the negative ion spray voltage was 2.50 kV, the sheath gas flow rate was 30 arb, and the auxiliary gas flow rate was 10 arb. The capillary temperature was 325 ℃, the full scan was performed with a resolution of 70,000, the scan range was 81–1000 m/z, and the HCD was used for secondary cracking, the collision voltage was 30 eV, and the dynamic elimination was used to remove unnecessary MS/MS information.

### Fecal DNA extraction and high-throughput 16S rDNA sequencing

The control group, the model group, and the THSWD-M group of rats were selected, each with 8 rats. After the animals were sacrificed, the abdominal cavity was opened, the intestines were separated, and the contents of the large intestine were cut out. After collecting the feces, put them in a freezing tube, immediately place them in liquid nitrogen for quick freezing, and then transfer and store them in liquid nitrogen or −80 ℃ refrigerator after quick freezing. The CTAB method was used to extract the total genomic DNA in the sample. Monitor DNA concentration and purity on a 1% agarose gel. We used sterile water to dilute the DNA to 1 ng/µL and used specific primers to amplify 16srrna/ITS genes in different regions (16sv4/16sv3/16sv3/16sv4/16sv4/16sv4/18sv4, ITS1/ITS2, arcv4). All PCR reactions were performed using 15 µL Phusion® high-fidelity PCR master mix (New England Biolabs); 2 µM forward and reverse primers and approximately 10 ng template DNA. The thermal cycle includes initial denaturation at 98 ℃ for 1 min, then denaturation at 98 ℃ for 10 s, annealing at 50 ℃ for 30 s, elongation at 72 ℃ for 30 s, and finally 72 ℃ for 5 min.

### Statistical analysis

The pharmacodynamics data was analyzed by SPSS 21.0 statistical software, expressed as ± s, and the comparison between multiple groups was performed by one-way ANOVA; when the variances of each group were uniform, the pairwise comparison between the means of multiple groups was used by the significant test, the correlation study adopts linear correlation analysis, *p < 0.05*, *p < 0.01* indicates that the difference is statistically significant.

Metabolomics data was converted to mzXML format (xcms input file format) through Proteowizard software (v3.0.8789), and the XCMS program package of R (v3.3.2) was used for peak identification and peak filtering (peaks filtration), peaks alignment (peaks alignment), the main parameters were bw = 5, ppm = 15, peak width = c (5,30), mzwid = 0.015, mzdiff = 0.01, method = “centWave”. Obtain a data matrix including mass to charge ratio (*m/z*), retention time, and peak area (intensity). Export the data to excel for subsequent analysis. In order to compare data of different magnitudes, batch normalization of peak area was performed on the data.

Mass spectrometry of metabolites in rat plasma first confirmed the exact molecular weight of the metabolites (molecular weight error < 5 ppm), and then the fragment information obtained according to the MS/MS model in the HumanMetabolome Database (HMDB) (http://www.hmdb.ca), Metlin (http://metlin.scripps.edu), massbank (http://www.massbank.jp/), LipidMaps (http://www.lipidmaps.org), mzclound (https://www.mzcloud.org), for further confirmation and identification of metabolites. Use the heatmap package in R (v3.3.2) to zoom the data set to obtain a hierarchical clustering diagram of the relative quantitative value of metabolites; use multivariate statistical analysis (software SIMCA-P (v13.0) and R language ropls package 1) Methods Partial Least Squares-Discriminant Analysis (PLS-DA) and Orthogonal Partial Least Squares Discriminant Analysis (OPLS-DA) were performed.

The gut microbiota data was first spliced with the original data, and a certain proportion of the interference data (Dirty Data) was filtered to obtain the effective data (Clean Data). Then, based on the valid data, perform OTUs (Operational Taxonomic Units) clustering and species classification analysis. According to the OTUs clustering results, on the one hand, species annotations were made on the representative sequence of each OTU, and the corresponding species information and species-based abundance distribution are obtained. At the same time, the abundance, alpha diversity calculation, Venn diagram, and other analyses of OTUs were performed to obtain the species richness and uniformity information in the sample and the common and unique OTUs information among different samples or groups. On the other hand, multiple sequence alignments of OTUs can be performed and phylogenetic trees can be constructed. Through dimensionality reduction analysis such as PCoA and NMDS and sample clustering tree display, the differences in community structure between different samples or groups can be explored.

To further explore the differences in community structure among grouped samples, LEfSe statistical analysis method was used to test the significance of differences in species composition and community structure of grouped samples. At the same time, the CCA/RDA/dbRDA analysis and the correlation analysis between the diversity index and environmental factors can also be combined with environmental factors to obtain environmental impact factors that significantly affect the community changes between groups. The annotation results of amplicons can also be associated with the corresponding functional database, and PICRUST software can be used to perform functional prediction and analysis on the microbial community in the ecological sample.

## Results

### UPLC content determination result of THSWD

The content of six components in THSWD water extract was determined by UPLC. The chromatographic results of the sample showed that the retention times of rehmannia glycoside D, amygdalin, hydroxysafflor yellow A, paeoniflorin, ferulic acid and ligustilide were 3.70, 19.36, 21.42, 24.36, 31.65, 41.35 min, respectively. The content determination results were 4.23, 0.11, 1.95, 2.43, 0.57, and 0.076 mg/g, respectively.

### The body weight of rats and the results of HE staining of the spleen

**Fig. 1:**
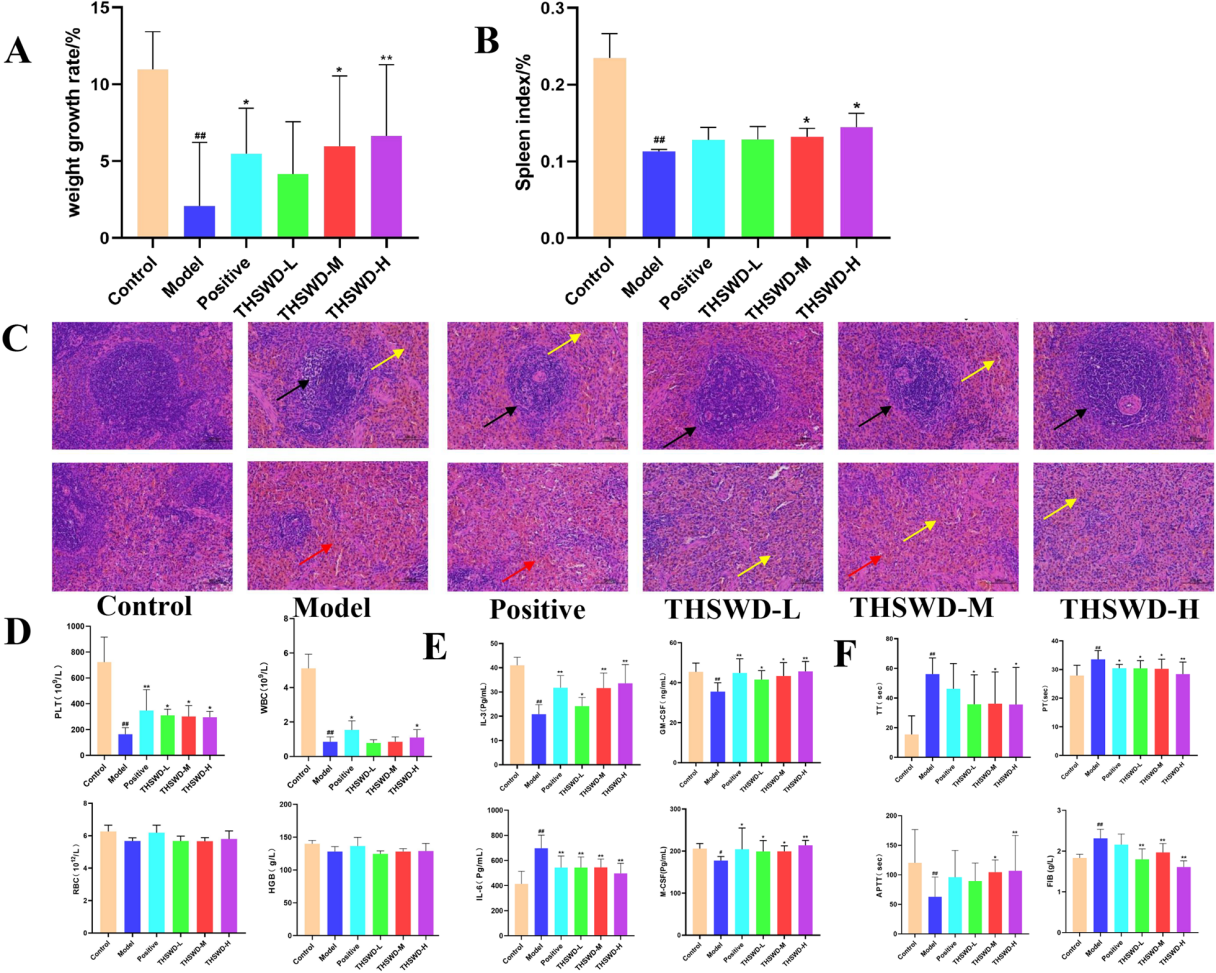
Changes in body weight, growth rate (**A**), and spleen index of rats in each group before and after modeling (**B**); STAINING section, results of spleen (**C**);WBC, PLT, RBC, and HGB levels of rats in each group (**D**); ≤ Serum hematopoietic related factors in each group of rats (**E**); Blood coagulation function index of each group (**F**). Compared with the model group, *p < 0.05, **p < 0.01; compared with the control group, ^*#*^*p < 0.05*, ^*##*^*p < 0.01*

The rats in the model group were induced to show signs of blood stasis after bathing in ice water, showing dark lips, dark red around the ears, dark purple nails, and dark red tongues. After the injection of cyclophosphamide, they moved slowly and shrank, the hair was erect and less shiny, and their face, eyes, ears, and tail were pale. Through the growth rate of body weight and spleen index before and after modeling (Fig. 1A, B), compared with the control group, the growth rate of body weight of the model group was small, and there was a significant difference (*p < 0.01*). The different dosage groups of THSWD were used for modeling. The rate of weight change before and after was higher than that of the model group, and the middle and high dose groups had a significant difference (*p < 0.05*). Compared with the control group, the spleen index of the model group was significantly lower (*p ≤ 0.01*). Compared with the model group, the different dosage groups of THSWD can improve the reduction of the spleen index caused by the modeling. The medium and high-dose groups of THSWD had significant differences compared with the model group (*p ≤ 0.05*).

The results of HE staining sections of the spleen of each group can be shown in Fig. 1C. Compared with the control group, the white pulp volume of the model group is reduced, the marginal band is not obvious (black arrow), the red pulp lymphocytes are greatly reduced, hemorrhage is widely seen (red arrow), and more brown-yellow pigmentation is seen (yellow arrow). Compared with the model group, the white pulp volume, marginal zone, red pulp lymphocytes, hemorrhage, and brown pigmentation in the THSWD administration groups were greatly improved.

### Test of blood routine and hematopoietic related factors in rats

The blood routine results of rats in each group are shown in Fig. 1D. The experimental results showed that, compared with the control group, the number of PLT and WBC in the model group was significantly reduced (*p ≤ 0.01*), an indication of blood deficiency. Compared with the model group, the different dose groups of THSWD can significantly improve the thrombocytopenia caused by modeling (*p ≤ 0.05*), and can improve the reduction of WBC count caused by modeling to some extent, but there was no significant difference.

The results of rat serum hematopoietic related factors GM-CSF, M-CSF, IL-3, and IL-6 are shown in Fig. 1E. Compared with the control group, the GM-CSF, M-CSF, and IL-3 in the serum of the model group are all significantly decreased (*p ≤ 0.05 or p ≤ 0.01*); compared with the model group, each group of THSWD can significantly increase GM-CSF, M-CSF, IL-3 in rat serum (*p ≤ 0.05 or p ≤ 0.01*). Compared with the control group, the rat serum IL-6 in the model group increased significantly (*p ≤ 0.01*); compared with the model group, each group of THSWD could significantly reduce the IL-6 in the rat serum (*p ≤ 0.01*).

### Detection of hemorheology and coagulation function indexes in rats

The results of plasma and whole blood viscosity of each group are shown in Tables 1 and 2. Compared with the control group, the model group has a significant increase in the viscosity of rat plasma and whole blood. Compared with the model group, each dose group of THSWD had a significant improvement effect (*p ≤ 0.05*). The coagulation function index results of each group are shown in Fig. 1F. Compared with the control group, the model group can significantly increase the TT, PT, and FIB and significantly reduce the APTT (*p ≤ 0.01*). It was speculated that the increase of TT and PT may be caused by the decrease of platelet content caused by modeling, while the increase of FIB and decrease of APTT may be caused by the combination of blood deficiency and cold coagulation blood stasis in the rat blood stasis model, caused by changes in its coagulation function. Compared with the model group, the THSWD group with different doses has a significant improvement effect (*p ≤ 0.05*).

**Table 1:** Whole blood viscosity results of each group

Group	Whole blood viscosity (mPa · s)				
	1 S^− 1^	5 S^− 1^	50 S^− 1^	100 S^− 1^	200 S^− 1^
Control group	13.69 ± 2.86	5.15 ± 1.38	2.20 ± 0.79	1.89 ± 0.72	1.69 ± 0.68
Model group	30.33 ± 6.19^##^	10.80 ± 2.16^##^	4.25 ± 0.96^##^	3.58 ± 0.86^##^	3.15 ± 0.79^##^
Positive group	21.18 ± 3.67**	7.77 ± 1.47**	3.19 ± 0.74**	2.71 ± 0.66*	2.42 ± 0.61*
THSWD-L group	24.18 ± 3.82**	9.12 ± 1.33*	3.90 ± 0.54	3.36 ± 0.47	3.01 ± 0.42
THSWD-M group	24.05 ± 3.34**	8.98 ± 1.14*	3.79 ± 0.55	3.24 ± 0.50	2.89 ± 0.47
THSWD-H group	23.96 ± 1.84**	8.97 ± 0.54*	3.79 ± 0.32	3.26 ± 0.31	2.90 ± 0.30

Compared with the model group, *p < 0.05, **p < 0.01; compared with the control group, ^*#*^*p < 0.05*, ^*##*^*p < 0.01*

**Table 2:** Plasma viscosity results of each group

Group	Plasma viscosity(mPa · s)				
	1 S^− 1^	5 S^− 1^	50 S^− 1^	100 S^− 1^	200 S^− 1^
Control group	12.64 ± 4.29	5.20 ± 1.22	2.50 ± 0.29	2.21 ± 0.22	2.04 ± 0.21
Model group	28.30 ± 5.90^##^	9.84 ± 1.64^##^	3.73 ± 0.47^##^	3.13 ± 0.38^##^	2.73 ± 0.33^##^
Positive group	18.04 ± 5.73**	6.79 ± 1.91**	2.89 ± 0.67**	2.49 ± 0.56**	2.22 ± 0.48*
THSWD-L group	19.33 ± 3.25**	7.25 ± 1.00**	3.07 ± 0.36*	2.64 ± 0.31*	2.35 ± 0.28
THSWD-M group	18.06 ± 2.17**	7.08 ± 0.85**	3.17 ± 0.41*	2.76 ± 0.37	2.49 ± 0.34
THSWD-H group	16.64 ± 3.04**	6.55 ± 0.96**	2.96 ± 0.36*	2.58 ± 0.32*	2.33 ± 0.29

Compared with the model group, *p < 0.05, **p < 0.01; compared with the control group, ^*#*^*p < 0.05*, ^*##*^*p < 0.01*

### Results of plasma metabolomics

**Fig. 2:**
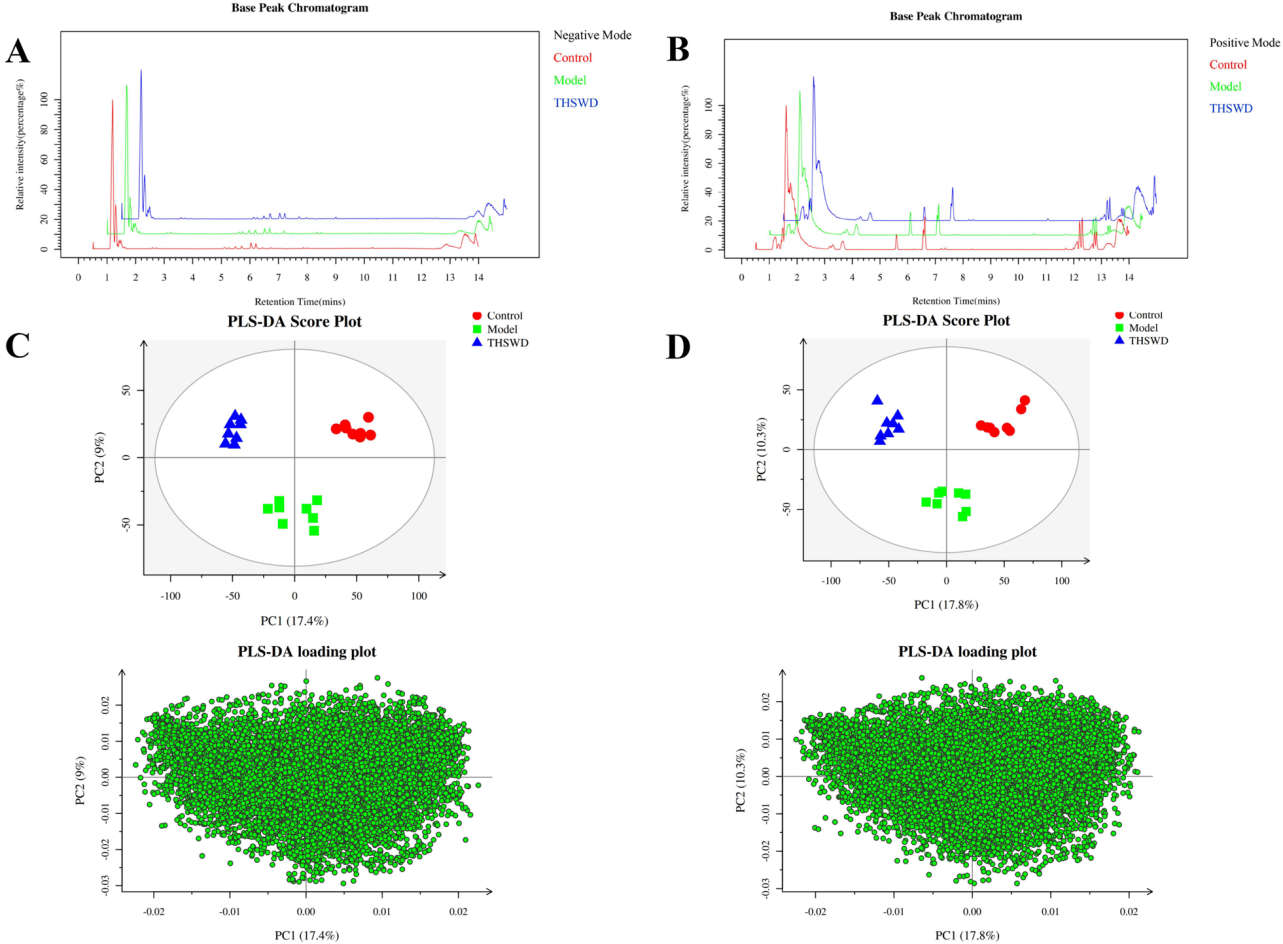
Basic peak chromatograms in positive ion mode (**A**); basic peak chromatogram in negative ion mode (**B**); PLS-DA score chart and load chart in positive ion mode (**C**); PLS-DA score chart and load chart in negative ion mode (**D**)

**Fig. 3:**
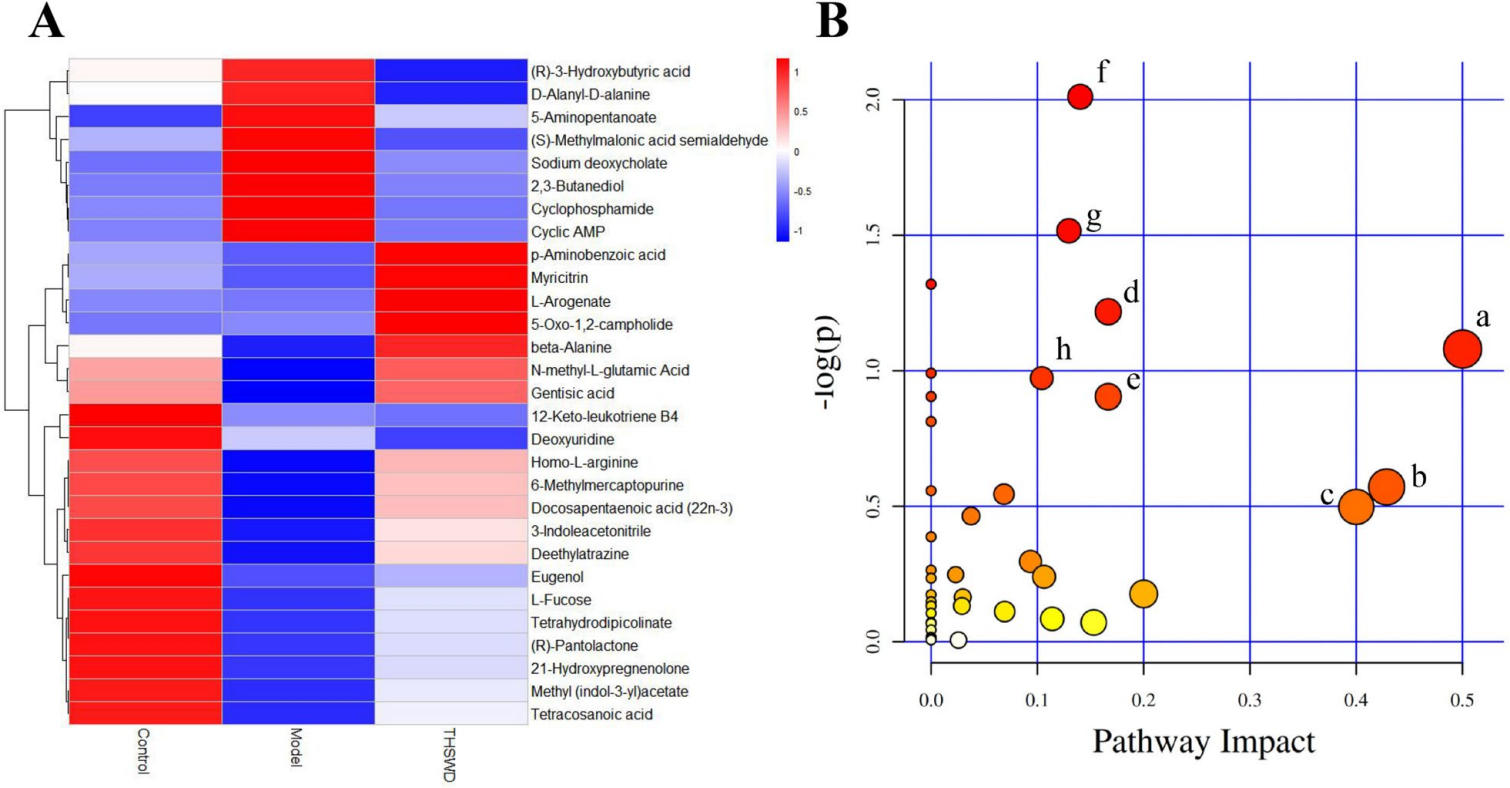
Heatmap showing the abundance of differential metabolites (**A**); results of metabolic pathway analysis, Main metabolic pathways interfered by THSWD (**B**) (a. Phenylalanine, tyrosine, and tryptophan biosynthesis; b. Taurine and hypotaurine metabolism; c. Ascorbate and aldarate metabolism; d. Riboflavin metabolism; e. Biotin metabolism; f. Arginine and proline metabolism; g. Phenylalanine metabolism)

When conducting metabolomics research based on mass spectrometry technology, in order to obtain reliable and high-quality metabolomics data, quality control (QC) is usually required. The dense distribution of QC samples on the PCA analysis chart shows that the data is reliable (Additional file 1: Fig. S1). The results of mass spectrometry data prove that the UHPLC-MS technology has good reproducibility. 13,008 precursor molecules were obtained in positive ion mode, and 12,912 precursor molecules were obtained in negative ion mode. The typical basic peak intensity chromatograms of the control group, model group, and THSWD group are shown in Fig. 2A and B. The difference between the groups was analyzed by PLS-DA, and the results showed that the classification effect was significant, and each group was separated from the other (ESI + R_2_Y = 0.995, Q_2_ = 0.863: ESI − R_2_Y = 0.988, Q_2_ = 0.912) as shown in Fig. 2C and D, revealed the obvious difference between the model group and control group. The THSWD group was close to the control group, which indicates that THSWD can significantly improve the plasma metabolism of rats with blood deficiency and blood stasis. For the identified metabolites, based on the screening indicators of *p ≤ 0.05* and VIP ≥ 1, twenty-three different metabolites were identified (Additional file 2: Fig. S2, Additional file 3: Fig. S3), and the heat map of differential metabolites is shown in Fig. 3A.

Next, the identification of metabolites was launched using an online metabolite database and self-built database to identify and screen metabolites through its accurate m/z fragment and MS/MS spectrum. Taking interesting variables (tR/s-*m/z* 85.9595–90.0554) to form the ESI^+^ data set as an example, the quasi-molecular ion of m/z 90.0554 [M + H]^+^. The molecular formula for the interesting variable was speculated to be C_3_H_7_NO_2_ by elemental composition analysis using Masslynx 4.1. The main fragment ions of C_3_H_7_NO_2_ were observed at *m/z* 72.06, 91.06, 90.06. In these fragments, the *m/z* 72.06 [M + H-H_2_O]^+^ and 91.06 [M + H]^+^ are dehydrated and hydrolyzed products.

Twenty-three metabolites related to THSWD for improving blood deficiency and blood stasis syndrome were screened and identified. The detailed information is shown in Table 3. THSWD can regulate the metabolic disorder induced by blood deficiency and blood stasis syndrome, and all potential biomarkers after THSWD administration intervention tend to restore the control group.

After modeling, (S)-Methylmalonic acid, a semialdehyde, which is involved in Propanoate metabolism, was significantly increased and beta-Alanine was significantly decreased in rats. Tetracosanoic acid and Docosapentaenoic acid (22n-3), which are involved in the biosynthesis of the unsaturated fatty acid metabolism pathway, were significantly reduced. Deoxyuridine and β-Alanine, which are involved in Pyrimidine metabolism, were significantly reduced. Participate in Synthesis and degradation of ketone bodies (R)-3-Hydroxybutyric acid is significantly increased. Gentiolic acid involved in tyrosine metabolism was significantly reduced. Cyclophosphamide, which is involved in drug metabolism cytochrome P450 and drug metabolism and other enzymes metabolic pathways, was significantly increased, and 6-Methylmercaptopurine was significantly decreased. Cyclic AMP, a different metabolite involved in purine metabolism, was significantly increased. 5-Aminopentanoate, which is involved in the metabolic pathways of Lysine degradation, Arginine, and proline metabolism, was significantly increased. After treatment with THSWD, these indicators can be significantly adjusted.

The MetaPA database was used to analyze the related metabolic pathways of the differential metabolites in the model group and the THSWD administration group. The results showed that THSWD had a significant regulatory effect on the eight imbalanced metabolic pathways caused by blood deficiency and blood stasis syndrome (Fig. 3B), which were phenylalanine, tyrosine and tryptophan biosynthesis, taurine and hypotaurine metabolism, ascorbic acid and alginate metabolism, riboflavin metabolism, biotin metabolism, arginine and proline metabolism, phenylalanine metabolism, pyrimidine metabolism.

**Table 3:** Differential metabolites in different groups (n = 8)

No	Name	VIP	RT/min	*m*/*z*	ppm	Molecular formula	KEGG Metabolic pathway	Ion mode	MS/MS	Fold change	
										Model/Control	THSWD/Model
M165T734	Eugenol	2.161	12.24	165.09104	0.216	C_10_H_12_O_2_	C10453	[M^+^H]^+^	165.09	0.357	1.424
M138T93	p-Aminobenzoic acid	1.938	1.55	138.05495	0.004	C_7_H_7_NO_2_	C00568	[M^+^H]^+^	138.06	0.629	4.412
M261T600	Cyclophosphamide	1.534	10.00	261.0317	1.448	C_7_H_15_Cl_2_N_2_O_2_P	C07888	[M^+^H]^+^	261.03, 189.05	3.226	0.325
M415T801_1	Sodium deoxycholate	1.732	13.34	415.2095	2.119	C_24_H_39_O_4_. Na	C11171	[M^+^H]^+^	119.09	5.182	0.198
M166T314	6-Methylmercaptopurine	1.738	5.24	166.04809	3.255	C_6_H_6_N_4_S	C16614	[M]^+^	166.05, 149.02	0.595	1.540
M172T653	Tetrahydrodipicolinate	1.736	10.89	172.07562	1.879	C_7_H_9_NO_4_	C03972	[M^+^H]^+^	172.08, 130.07	0.345	1.819
M188T627	Deethylatrazine	1.684	10.45	188.07057	4.202	C_6_H_10_C_l_N_5_	C06559	[M^+^H]^+^	188.07, 170.06	0.272	2.889
M189T653	Methyl (indol-3-yl) acetate	1.807	10.89	189.07826	0.236	C_11_H_11_NO_2_	C20635	[M]^+^	130.07	0.311	2.063
M102T56	(S)-Methylmalonic acid semialdehyde	1.098	0.94	102.03393	5.644	C_4_H_6_O_3_	C06002	[M]+	103.09	3.178	0.151
M156T775	3-Indoleacetonitrile	1.382	12.92	156.12099	2.569	C_10_H_8_N_2_	C02938	[M]^+^	133.10, 115.96	0.599	1.484
M160T424	d-Alanyl-d-alanine	1.381	7.07	160.09693	0.658	C_6_H_12_N_2_O_3_	C00993	[M]^+^	85.07, 76.04	1.423	0.419
M227T585	l-Arogenate	1.479	9.75	227.08144	4.965	C_10_H_13_NO_5_	C00826	[M]^+^	226.05, 213.06, 90.98	0.242	62.098
M87T205	(R)-3-Hydroxybutyric acid	1.38	3.42	87.044682	2.154	C_4_H_8_O_3_	C01089	[M^+^H-H_2_O]^+^	87.04, 69.07	1.310	0.525
M90T86	Beta-Alanine	1.496	1.43	90.055448	1.579	C_3_H_7_NO_2_	C00099	[M^+^H]^+^	91.06, 72.06	0.844	1.375
M155T163	2,3-Butanediol	1.356	2.71	154.9903	0.021	C_4_H_10_O_2_S_2_	C00265	[M^+^H]^+^	131.97, 113.96	2.406	0.361
M116T219	5-Aminopentanoate	2.293	3.65	116.06989	4.836	C_5_H_11_NO_2_	C00431	[M^−^H]^−^	116.07, 98.2	4.785	0.464
M349T678	Tetracosanoic acid	1.999	11.30	349.23791	1.253	C_24_H_48_O_2_	C08320	[M^−^H_2_O^−^H]^−^	349.24, 287.24	0.322	1.905
M227T236	Deoxyuridine	1.698	3.94	227.06669	2.177	C_9_H_12_N_2_O_5_	C00526	[M-H]^−^	184.00, 159.01	0.698	1.348
M153T436	Gentisic acid	1.822	7.26	153.01804	3.218	C_7_H_6_O_4_	C00628	[M-H]^−^	153.02, 109.03	0.456	2.425
M142T100	*N*-methyl-l-glutamic Acid	1.564	1.67	142.05045	0.246	C_6_H_11_NO_4_	C01046	[M-H_2_O-H]^−^	142.06, 116.07	0.698	1.547
M328T320	Cyclic AMP	1.673	5.33	328.04498	0.753	C_10_H_12_N_5_O_6_P	C00575	[M-H]^−^	328.05, 134.05	0.478	0.703
M463T434	Myricitrin	1.708	7.24	463.08663	3.389	C_21_H_20_O_12_	C10108	[M-H]^−^	316.02, 287.04	0.788	2.310
M187T95_2	Homo-l-arginine	1.635	1.58	187.11891	4.952	C_7_H_16_N_4_O_2_	C01924	[M-H]^−^	145.10	0.579	1.608
M329T830	Docosapentaenoic acid (22n-3)	1.722	13.83	329.24856	0.113	C_22_H_34_O_2_	C16513	[M-H]^−^	329.25, 285.26	0.608	1.476

### High-throughput sequencing of 16S rDNA

The dilution curve verifies the rationality of the amount of sequencing data and indirectly reflects the abundance of species in the sample. The flat curve indicated that the depth of sequencing is enough (Additional file 4: Fig. S4). With the increase of sequencing time, the number of OTUs in each group also increased, but the increase rate showed a downward trend, indicating that all samples in this study had sufficient sequences, and the number of sequencing reads covered most of the microorganisms in the samples, which can be used for data analysis. Species cumulative boxplots were used to judge whether the sample size was sufficient. Under the premise of sufficient sample size, species richness was predicted using the species cumulative boxplot. As can be seen from the species accumulation boxplot, as the sample size increases, if the position of the graph tends to be flat, it means that the species in the environment does not increase significantly with the increase of the sample size, indicating that the sampling is sufficient to carry out data analysis (Additional file 5: Fig. S5).

**Fig. 4:**
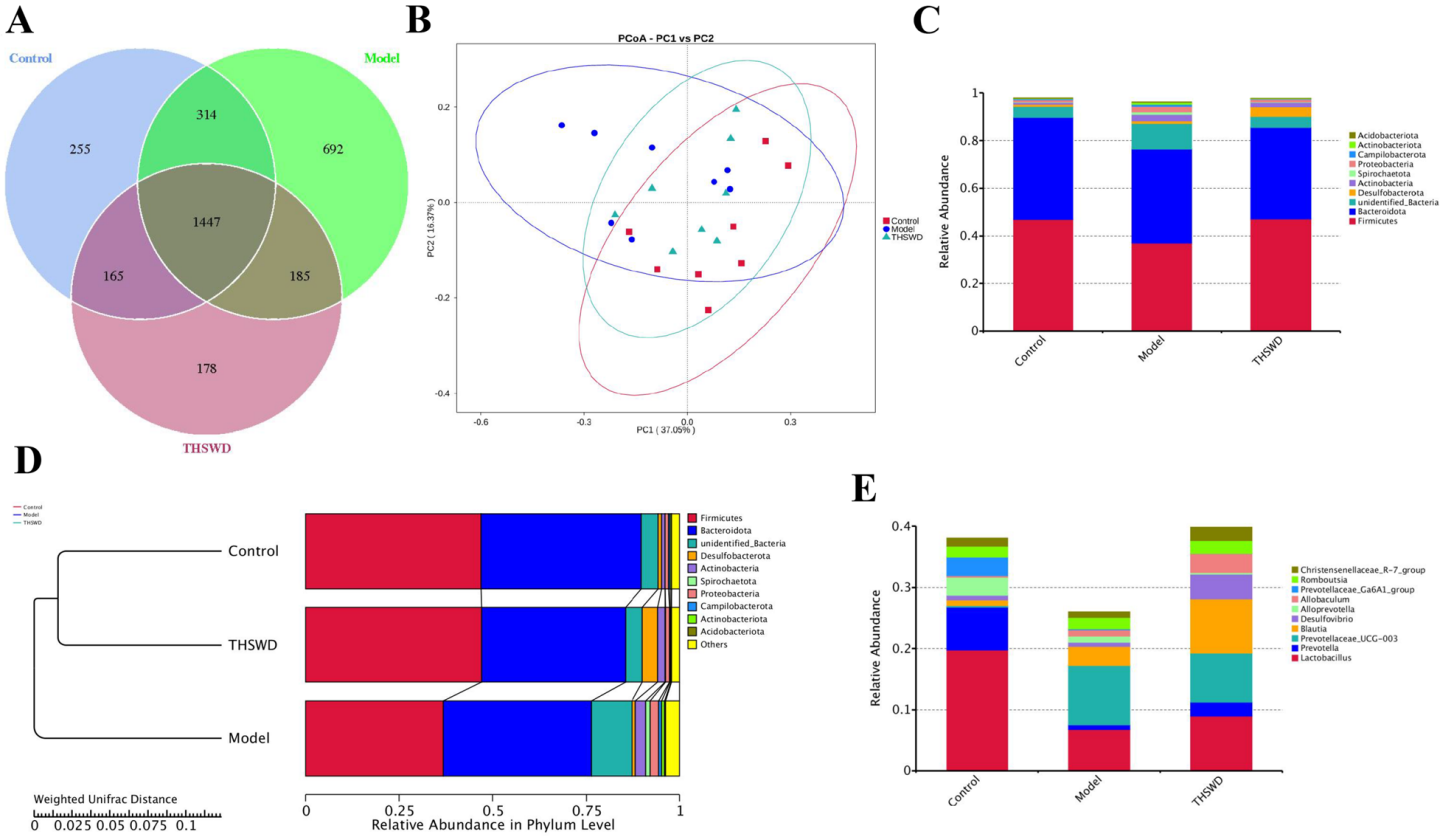
OUTs Venn diagram (**A**); PCoA analysis diagram (**B**); UPGMA clustering tree (**D**); histogram of species abundance in each group Phylum level (**C**); histogram of species abundance in each Genus level (**E**)

In order to explore the relationship between THSWD in the treatment of blood deficiency and blood stasis syndrome and gut microbiota, we analyzed the fecal flora of each group of rats. The species composition of each sample was studied, and the effective sequences of all samples were clustered by OTUs (Operational Taxonomic Units) at a similarity level of 97%, and then the species of OTUs were annotated to obtain 3236 OTUs. According to the results of OTUs obtained by clustering and research needs, analyze the common and unique OTUs between different groups and draw the Venn diagram (Fig. 4A). From the figure, the difference in the number of OTUs in the three groups can be initially seen. The model group has 692 unique OTUs, the control group has 255 unique OTUs, and the THSWD group has 178 unique OTUs. It can be seen from the Venn diagram that, compared with the control group, there were 877 unique OTUs in the model group and 363 unique OTUs in the THSWD group, which was smaller than the difference between the model group and the control group. THSWD can regulate the composition and abundance of intestinal microflora in rats with blood deficiency and blood stasis syndrome, which may be the mechanism of its treatment of blood deficiency and blood stasis syndrome.

The diversity of microbial communities in the samples was analyzed by Alpha Diversity. The Chao1 and ACE indices responded to intestinal community richness, and the Shannon and Simpson indices responded to the diversity of the flora (Table 4). The results showed that Chao1, ACE, and Shannon indices were significantly higher in the model group compared with the control group (*p < 0.01*); and Chao1, ACE, and Shannon indices were significantly lower in the THSWD administration group compared with the model group (*p < 0.01, 0.05*). These results indicated that the abundance and diversity of intestinal microbiota increased and the intestinal microbiota became disturbed after the rats were modeled through blood deficiency and blood stasis syndrome. THSWD can promote the decrease of gut microbiota richness and diversity, and may promote its return to a normal state.

**Table 4:** Influence of THSWD on Alpha Diversity Index of gut microbiota (x ± s, n = 8)

Group	Dose (g/kg)	Diversity index			
		Chao1	ACE	Shannon	Simpson
Control group	–	950.97 ± 205.70	956.42 ± 204.21	6.14 ± 0.50	0.94 ± 0.04
Model group	–	1303.09 ± 78.61^##^	1321.55 ± 86.80^##^	6.82 ± 0.25^##^	0.96 ± 0.01
THSWD group	10	841.58 ± 168.21^**^	1048.39 ± 229.22^*^	6.16 ± 0.41^**^	0.95 ± 0.02

Compared with the control group, ^*#*^*p < 0.05*, ^*##*^*p < 0.01*; compared with the model group, *p < 0.05, **p < 0.01

PCoA analysis and UPGMA cluster analysis revealed differences in gut microbiota between the three groups. In the PCoA score chart (Fig. 4B), the percentages explained by PC1 and PC2 to the overall variance are 37.05% and 16.37%. PCoA analysis and UPGMA cluster analysis (Fig. 4D) to investigate the similarity between different samples, and can also construct a cluster tree of samples by clustering the samples. The results show that the control group and the THSWD group are clustered into one category, indicating the relative abundance of gut microbiota in the state of blood deficiency and blood stasis syndrome changes greatly, and the intervention of THSWD can effectively alleviate this change.

According to the results of species annotations, select the top 10 species with the largest abundance in each group in Phylum and Genus, and generate a columnar cumulative map of the relative abundance of species to visually view the different classification levels of each sample. The proportion of species with higher relative abundance is shown in Fig. 4C and E.

At the phylum level, Firmicutes and Bacteroidota account for more than 80% of species abundance. Compared with the control group, the relative abundance of Firmicutes in the model group decreased by 21.4%, the relative abundance of Bacteroidota decreased by 7.8%, unidentified_Bacteria increased by 1.4 times, Actinobacteria increased by 1.85 times, Spirochaetota increased by 19 times, Proteobacteria increased by 1.48 times, and Campilobacterota increased by 1.18 Times. Compared with the model group, the relative abundance of Firmicutes in the THSWD group increased by 27.5%, unidentified_Bacteria decreased by 59.5%, Actinobacteria decreased by 29.8%, Spirochaetota decreased by 84.0%, Proteobacteria decreased by 65.2%, and Campilobacterota decreased by 72.1%. At the genus level, compared with the control group, the relative abundance of Lactobacillus in the model group decreased by 65.9%, Prevotella decreased by 89.4%, Prevotellaceae_UCG-003 increased by 33.6 times, and Christensenel decreased by 29.2%; compared with the model group, Lactobacillus increased in THSWD group 32.2%, Prevotella increased by 2.13 times, Prevotellaceae_UCG-003 decreased by 17.9%, and Christensenellaceae_R-7_group increased by 1.3 times.

MetaStat method was used to analyze the abundance data of species between groups. Species with significant differences were screened based on q-values. The relative abundance of each sample group differed significantly at the level of phylum, family, and genus (Table 5). The relativeabundances of Actinobacteriota and Deferribacteres in the model group were significantly higher than the control group at the phylum level. At the family level, the relative abundance of Rikenellaceae, Rs-E47_termite_group, and Prevotellaceae was significantly higher than the model group. At the genus level, the relative abundances of Prevotellaceae_UCG-004, Rikenellaceae_RC9_gut_group, Bilophila, and Odoribacter were significantly higher than the model group, while the relative abundance of [Eubacterium]_siraeum_group, [Bacteroides]_pectinophilus_group, Marvinbryantia were significantly lower.

**Table 5:** Species relative abundance (percentage) of each group (n = 8)

Gut microbiota	Control	Model	THSWD	q^a^	q^b^
Actinobacteriota(Phylum)	0.353	0.852	0.270	0.0400	0.0200
Deferribacteres(Phylum)	0.00561	0.148	0.0123	0.0120	0.0430
Rikenellaceae (Family)	0.355	2.100	0.282	0.0156	0.0138
Rs-E47_termite_group (Family)	0.00508	0.0705	0.00347	0.0156	0.0258
Prevotellaceae(Family)	0.000534	0.00722	0.000267	0.0156	0.0138
Prevotellaceae_UCG-004 (genus)	0.000802	0.0128	0.00134	0.0100	0.0130
Rikenellaceae_RC9_gut_group (genus)	0.0711	1.950	0.197	0.0104	0.0098
[Eubacterium]_siraeum_group (genus)	0.202	0.0214	0.169	0.0104	0.0759
[Bacteroides]_pectinophilus_group (genus)	0.299	0.0492	0.239	0.0186	0.0620
Marvinbryantia (genus)	0.176	0.0681	0.282	0.0260	0.00900
Bilophila (genus)	0.0176	0.0786	0.0142	0.0330	0.0140
Odoribacter (genus)	0.0139	0.0847	0.0152	0.00100	0.0200

q^a^ represents the comparison between the model group and control group; q^b^ represents the comparison between the THSWD group and the model group

### The potential relationship between plasma metabolites and gut microbiota

**Fig. 5:**
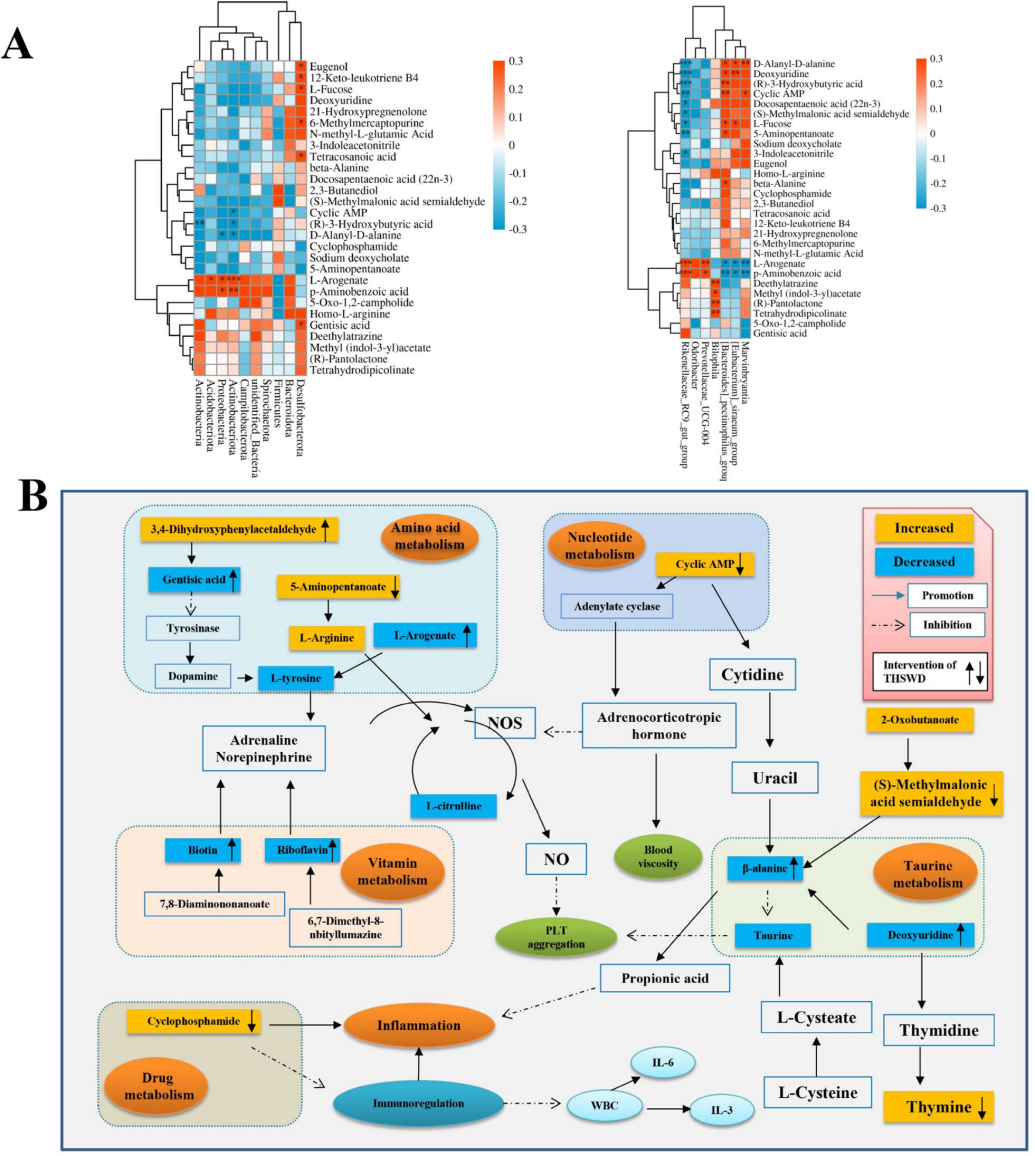
Correlation diagram between the relative abundance of gut microbiota and potential biomarkers, the correlation between the phylum level difference flora and 29 different metabolites; the difference and correlation between genus level flora and 29 different metabolites (**A**); the color of the box represents the changing trend of the model group and the control group, in which yellow represents promotion, and blue represents inhibition. The arrow on the right of the metabolite in the box indicates the trend of THSWD and the model group; the up arrow represents promotion, and the down arrow represents inhibition (**B**)

We analyzed the association between gut bacteria and host metabolism by calculating the Spearman correlation coefficient and found that some flora has a strong correlation with metabolites (*p > 0.3 or p < -0.3*) (Fig. 5A). The phylum level includes Actinobacteriota, Acidobacteriota, Proteobacteria, Actinobacteria, Desulfobacterota, and the genus level includes Prevotellaceae_UCG-004, Rikenellaceae_RC9_gut_group, [Eubacterium]_siraeum_group, [Bacteroides]_pectinophilus_group, Marvinbryantia. These microflora are closely related to the metabolic pathways and may participate in interference with blood deficiency and blood stasis syndrome. Regarding potential biomarkers, Eugenol, 12-Keto-leukotriene B4, Tetracosanoic acid, 6-Methylmercaptopurine, Cyclic AMP, (R)-3-Hydroxybutyric acid, l-Arogenate, d-Alanyl-d-alanine, P-Aminobenzoic acid, Gentisic acid, 3-Indoleacetonitrile, beta-Alanine, Methyl (indol-3-yl) acetate, Deethylatrazine, Deoxyuridine, Docosapentaenoic acid (22n-3), (S)-Methylmalonic acid semialdehyde, 5-Aminopentanoate, Tetrahydrodipicolinate are closely related to the gut microbiota. The results are shown in Fig. 5A. At the phylum level, l-Arogenate, *p*-Aminobenzoic acid, and Actinobacteriota were significantly positively correlated, while Cyclic AMP (R)-3-Hydroxybutyric acid, d-Alanyl-d-alanine and Actinobacteriota were significantly negatively correlated. At the genus level, l-Arogenate and *p*-Aminobenzoic acid were significantly positively correlated with Prevotellaceae_UCG-004 and Rikenellaceae_RC9_gut_group, but were significantly negatively correlated with [Eubacterium]_siraeum_group, [Bacteroides]_pectinophilus_group, Marvinbryantia. It can be seen from the results that the metabolites were closely related to the gut microbiota. THSWD affected the host blood system through the mutual adjustment of these two factors and relieved the blood deficiency and blood stasis syndrome in rats.

## Discussion

The rational combination of Chinese herbal medicines is key to the efficacy of Chinese medicine prescriptions [24]. THSWD consists of six botanical drugs. *Rehmanniae Radix*, which nourishes yin and blood as a monarch medicine, modern pharmacological studies have found that it has anti-inflammatory, anti-oxidative, anti-apoptotic and other pharmacological effects [25]; *Angelicae Sinensis Radix*, a minister medicine for nourishing blood and promoting blood circulation, which not only helps the monarch medicine warm and nourish the blood, but also promotes blood circulation and removes blood stasis [26]; *Paeoniae Radix Alba*, which nourishes the blood and softens the liver, modern pharmacological studies have shown that it has anti-inflammatory, analgesic and other pharmacological effects [27]; *Persicae Semen* and *Carthami Flos*, which promote blood circulation and remove blood stasis; and *Chuanxiong Rhizoma*, which promotes qi and blood circulation, and relieves pain [28]. The combination of the six medicines can nourish blood without stagnation and invigorate blood circulation without damaging it. The whole prescription can nourish the blood, promote blood circulation, remove blood stasis, and is suitable for various diseases caused by blood deficiency and blood stasis syndrome.

Blood deficiency is a common syndrome in anemia, arrhythmia, hypertension, neuropathic headache, and other diseases. According to Wang Qingren’s “YiLin GaiCuo,” blood stasis can be divided into five aspects: head, chest, abdomen, abdomen, limbs, and meridians, according to different lesions. The representative diseases of blood stasis are cerebral infarction, coronary heart disease, angina pectoris, cirrhosis, menstrual disorders, and rheumatoid arthritis.

Based on the analysis of etiology and clinical characteristics, modern medicine believes that blood deficiency syndrome can be seen in iron deficiency anemia, megaloblastic anemia, renal anemia, aplastic anemia, and bone marrow suppression caused by radiotherapy and chemotherapy. Modern medical indicators of blood deficiency syndrome mostly manifest as a decrease in the number of blood cells, a decrease in the level of hematopoietic-related factors, and a decrease in blood coagulation function. Blood stasis syndrome is accompanied by pathological changes such as increased blood viscosity, increased hematocrit, and increased platelet aggregation. Some studies believe that in addition to the above-mentioned pathological abnormalities, blood stasis syndrome is also related to vascular endothelial cell damage, atherosclerosis, ischemia, and hypoxia, thrombosis, microcirculation disorders, inflammatory pathological processes, and other pathological changes [29].

### Animal model of blood deficiency and blood stasis syndrome

THSWD is often used in women with irregular menstruation, dysmenorrhea, or menstrual blood clots. Women’s physiques are more inclined to be cold and weak, so women often experience blood deficiency and blood stasis simultaneously. Therefore, the preparation of a cold coagulation blood stasis model using an ice water bath is more in line with the traditional application of THSWD, and the model has a significant effect on the platelet aggregation rate and coagulation function. We compared the method of creating blood deficiency caused by bloodletting and cyclophosphamide injection through preliminary experiments and found that THSWD had better efficacy in a cyclophosphamide-induced model. The bloodletting model often manifests as a significant reduction in the number of red blood cells (RBCs) and white blood cells (WBCs), which has little effect on PLTs and has problems such as difficult control of bloodletting, large individual differences, and animal susceptibility to acute blood loss shock. Cyclophosphamide is a cytotoxic chemical drug that can inhibit the hematopoietic function of bone marrow. Evaluation of this model focuses on the observation of molecular indicators of the bone marrow hematopoietic process. The microstructure of the bone marrow changes, the activity of bone marrow hematopoietic reconstitution is reduced, the hematopoietic microenvironment is destroyed, and the number of bone marrow nucleated cells and their proliferation rate decline, resulting in a total decline in the number of various types of cells [30]. Based on a clinical foundation and preexperimental research, it was also found that THSWD had a greater impact on the number and aggregation of platelets in the blood. Therefore, we used an ice water bath combined with cyclophosphamide to prepare a blood deficiency and blood stasis rat model.

In this experimental study, we found that in the blood deficiency and blood stasis syndrome model, the weight growth rate of rats decreased, and the number of rats PLT and WBC were reduced, the levels of hematopoietic-related factors, including GM-CSF, M-CSF, IL-3, and IL-6 were reduced. In addition, modeling damage to the spleen of the rat can significantly increase the viscosity of the rat plasma and whole blood and reduce the blood coagulation function of the rat. After THSWD intervention, it increased the levels of hematopoietic-related factors GM-CSF, M-CSF, IL-3, and IL-6 in the serum of rats, and increased the coagulation index of rats, which further clarified that THSWD can improve the related functional indicators of blood deficiency and blood stasis syndrome.

We selected GM-CSF, M-CSF, IL-3, and IL-6 as hematopoiesis-related indicators. IL-3 promotes the proliferation and differentiation of hematopoietic stem cells into blood cells. IL-6 acts synergistically with IL-3 to stimulate bone marrow megakaryocyte DNA synthesis and promote the differentiation and proliferation of bone marrow megakaryocytes into mature platelets. M-CSF is released by the fusion of the precursor cells of monocytes and macrophages of the hematopoietic system and regulates the proliferation and differentiation of hematopoietic progenitor cells, especially monocytes and macrophages. GM-CSF has a stimulating effect on the proliferation and differentiation of bone marrow progenitor cells to mature granulocytes and can promote the differentiation of bone marrow progenitor cells to granulocytes (including neutrophils and eosinophils), megakaryocytes (common progenitor cells of myelomonocytic and monocyte cell lineages), and promote the proliferation and maturation of hematopoietic-oriented stem cells the above lineages. THSWD can improve the blood deficiency symptoms of rats by promoting the production of hematopoietic related factors GM-CSF, M-CSF, IL-3, and IL-6. Plasma and whole blood viscosity were evaluated to detect the state of blood stasis in rats. Whole-blood viscosity is a comprehensive manifestation of plasma viscosity, hematocrit (specific) volume, red blood cell deformability and aggregation ability, and platelet and WBC rheological properties. Plasma viscosity is an important factor affecting the viscosity of whole blood. The detection of thrombin time, prothrombin time, fibrinogen content, and activated partial thromboplastin time can be used to determine coagulation function, while thrombin time is used to understand whether the fibrin in plasma contains a sufficient amount of fibrinogen. The prothrombin time reflects whether exogenous coagulation is normal. Plasma fibrinogen is in the final stage of coagulation, in which soluble fibrinogen is transformed into insoluble fibrin, which causes blood coagulation. Activated partial thromboplastin time reflects whether endogenous coagulation is normal. Therefore, four coagulation indices were used to comprehensively assess the coagulation function of rats. THSWD can reduce the viscosity of plasma and whole blood to relieve the symptoms of blood stasis in rats, while also having a regulatory effect on the coagulation function of rats.

### Discussion of the results of metabolomics

The metabolomics results showed that THSWD has a callback effect on 26 metabolites of blood deficiency and blood stasis (as shown in Fig. 5B), which involves the regulation of amino acid metabolism, nucleotide metabolism, vitamin metabolism, and drug metabolism.

### Amino acid metabolism

As a precursor of energy metabolism, amino acids can increase ATP formation and play an important role in the body. Metabolomics results showed that 5-aminovalerate was significantly increased in rats with blood deficiency and blood stasis syndrome, while l-arginine, l-tyrosine, gentisic acid, l-atrogenate, tetrahydrodipicolinate, β-alanine, and taurine were significantly reduced, causing phenylalanine, tyrosine, and tryptophan biosynthesis, arginine and proline metabolism, phenylalanine metabolic pathways, and taurine and hypotaurine metabolism to be disordered. After THSWD administration, the content of 5-Aminopentanoate decreased, and the content of l-arginine, gentisic acid, l-atrogenate, and β-alanine increased and were close to those of the control group.

l-Tyrosine is an aromatic amino acid and a precursor of catecholamine neurotransmitters such as dopamine, epinephrine, and norepinephrine. l-tyrosine plays an important role in growth and metabolism regulation, hormone secretion regulation, blood pressure regulation, and the maintenance of central nervous system function. Epinephrine and norepinephrine can cause vasoconstriction and increase blood viscosity and stasis. Gentisic acid inhibits tyrosinase activity, and THSWD inhibits tyrosinase activity by increasing the gentisic acid content, inhibiting the production of epinephrine and norepinephrine, and relieving blood stasis.

Modern medical research has shown that the main cause of blood stasis syndrome is chronic inflammation caused by the obstruction of blood circulation. Therefore, blood stasis syndrome can be suppressed by promoting blood circulation and regulating immune response and inflammation. Arginine metabolism affects blood stasis syndrome by influencing inflammation. Arginine is involved in the conversion of nitric oxide synthase (NOS) into NO in the body, and NOS catalyzes the production of the substrate l-arginine to produce NO [31]. NO is a key signaling molecule that regulates the function of the cardiovascular system and has biological effects including relaxation of vascular smooth muscle, inhibition of smooth muscle cell proliferation, and inhibition of platelet adhesion and aggregation [32]. The expression of the NOS subtype iNOS, required for the conversion of arginine to NO, is induced by the pro-inflammatory cytokines IL-1, TNF-α, and TNF-γ. The l-arginine content was significantly reduced in the plasma of rats with blood deficiency and blood stasis syndrome, indicating that the l-arginine-NO pathway is disordered, leading to blood stasis symptoms. l-arginine is increased after THSWD administration, suggesting that THSWD inhibits the expression of pro-inflammatory factors and reduces the expression of iNOS, which increases arginine and regulates the l-arginine-NO pathway.

Taurine can inhibit the aggregation of PLTs induced by sodium adenosine diphosphate, collagen, and epinephrine. β-alanine is a taurine transporter inhibitor, and β-alanine can inhibit taurine transport, inhibit PLT aggregation, and relieve blood stasis. Following THSWD administration, the β-alanine level in rat plasma increased and approached that of the control group, indicating that THSWD inhibited PLT aggregation through the taurine metabolic pathway, thereby alleviating the symptoms of blood stasis.

### Vitamin and nucleotide metabolism

Vitamins are a class of organic compounds that are necessary for maintaining health and play an important role in metabolism. Based on the metabolomic results, we found four metabolites closely related to vitamin metabolism. d-glucuronic acid and hydroquinone increased significantly in the blood deficiency and blood stasis model groups, while riboflavin and biotin decreased significantly. Riboflavin (vitamin B2) can improve the body’s utilization of protein, promote growth and development, regulate the secretion of adrenaline, and have a regulatory effect on vascular smooth muscle and PLT aggregation.

The results of metabolomics demonstrate that the pyrimidine metabolism in rats with blood deficiency and blood stasis syndrome is abnormal, which is mainly manifested as a significant decrease in deoxyuridine and β-alanine, and a significant increase in cyclic AMP. In the state of blood stasis, the body’s qi and blood do not function smoothly, and thyroid and adrenal cortex functions are reduced. Moreover, endocrine hormones act on adenylates cyclase to reduce its activation, resulting in the obstruction of the production of cyclic AMP and cyclic GMP, which in turn affects the body’s health-related signal transduction function. THSWD administration can significantly decrease cAMP levels.

### Discussion of the results of the gut microbiota

The 16S rDNA sequencing analysis of the gut microbiota showed that the relative abundance of Firmicutes and Bacteroidota was significantly reduced in the model group, as was the Firmicutes/Bacteroidetes value (F/B value). Firmicutes and Bacteroidetes play important roles in the regulation of host energy metabolism [33, 34]. The F/B value increased significantly after THSWD administration, indicating that the effect of THSWD in treating blood deficiency and blood stasis syndrome may be mediated by the gut microbiota. The relative abundances of Actinobacteria, Spirochaetota, Proteobacteria, Campilobacterota, and other pathogenic bacteria in the model group were significantly increased. Actinobacteria, Spirochaetota, Proteobacteria, and Campilobacterota induce gastrointestinal inflammation and autoimmune diseases. At the genus level, Lactobacillus and Prevotella levels were significantly reduced in the model group. Lactobacillus can enhance immunity and promote digestion and absorption [35, 36], and the relative abundance of Lactobacillus was significantly increased after THSWD administration. THSWD can improve the symptoms of spleen and stomach weakness caused by blood deficiency and stasis by promoting the growth of probiotics.

### Relationship between metabolomic outcomes and gut microbiota

We combined 16S rDNA sequencing analysis and non-targeting to identify changes in the intestinal microbiota and metabolites of rats with blood deficiency and blood stasis syndrome and identify the mechanism of action of THSWD in this model. The F/B value and relative abundance of Firmicutes and Bacteroidota were significantly reduced in the model group. Firmicutes and Bacteroidetes play important roles in regulating host energy metabolism. Propionic acid is formed via the acrylic acid pathway and inhibits TNF-α expression and intestinal inflammation. Metabolomics results showed that blood deficiency and blood stasis syndrome can cause disturbances in the metabolic pathways of propionate and butyrate, which is consistent with the results of the gut microbiota. The F/B value increased significantly after THSWD administration, indicating that the effect of THSWD in treating blood deficiency and blood stasis syndrome may be mediated by the gut microbiota. At the genus level, Lactobacillus and Prevotella were significantly reduced in the blood deficiency and stasis model groups. Lactobacillus can enhance immunity and promote digestion. These are the main beneficial bacteria for amino acid metabolism. The weakened spleen and stomach of rats with blood deficiency and blood stasis syndrome and the low weight growth rate may be related to the decrease in beneficial intestinal bacteria. At the same time, metabolomic results show that blood deficiency and blood stasis syndrome can cause disorders of lysine degradation, arginine, proline metabolism, and amino acid biosynthesis pathways, all of which are consistent with our gut microbiota results. The imbalance of Lactobacillus is related to the absorption of fatty acids, the metabolism of propionate and butyrate, and the biosynthesis of unsaturated fatty acids is disordered in the blood deficiency and blood stasis models. The relative abundance of Lactobacillus increased significantly after THSWD administration, indicating that THSWD may promote the growth of beneficial bacteria.

## Conclusion

In this study, we innovatively established a model of blood deficiency and blood stasis syndrome in TCM. After modeling, the levels of WBCs, PLTs, GM-CSF, M-CSF, IL-3, and IL-6 decreased, as did the blood rheology and blood coagulation function decreased. and after modeling, the metabolism of amino acids, fatty acids and steroids were disordered, and the changes in the diversity and abundance of gut microbiota. THSWD can regulate the biosynthesis of phenylalanine, tyrosine, and tryptophan, as well as the metabolism of taurine, hypo, taurine, ascorbic acid, alginate, riboflavin, biotin, acid and proline, phenylalanine, and pyrimidine, improving the diversity and abundance and adjusting the structure of the gut microbiota, thereby alleviating the state of blood deficiency and blood stasis. This study analyzed the potential metabolic biomarkers and key functional bacteria of blood deficiency and blood stasis syndrome and clarified the therapeutic effect and mechanism of THSWD. Our results clarified the pathogenesis of blood deficiency and blood stasis syndrome in TCM, the mechanism of action of THSWD to some extent, and laid the foundation for the clinical application of THSWD in the treatment of disease.

## Supplementary Information

**Additional file 1.** PCA score chart of QC samples.

**Additional file 2.** MS/MS mapping of twenty-three different metabolites.

**Additional file 3.** Histogram of differential metabolites for each group.

**Additional file 4.** Rank Abundance Curve.

**Additional file 5.** Species accumulation boxplot.

## Data Availability

Not applicable.

## Abbreviations

APTT: Activated partial thromboplastin time
EDTA: Ethylene diamine tetra acetic acid
F/B: Firmicutes/Bacteroidetes
FIB: Fibrinogen content
GM-CSF: Granulocyte macrophage colony stimulating factor
HE: Hematoxylin-eosin
HGB: Hemoglobin
UPLC: Ultra performance liquid chromatography
IL-1: Interleukin-1
IL-3: Interleukin-3
IL-6: Interleukin-6
M-CSF: Macrophage colony stimulating factor
NMDS: Non-metric multidimensional scaling analysis
NO: Nitric oxide
OTUs: Operational taxonomic units
PCoA: Principal coordinate analysis
PLT: Platelet
PT: Prothrombin time
RBC: Red blood cell
TCM: Traditional Chinese medicine
THSWD: Taohong Siwu Decoction
TNF-α: Tumor necrosis factor-α
TT: Thrombin time
WBC: White blood cell
