# Supplementary figures and images for: Exploring the mechanism of Taohong Siwu Decoction on the treatment of blood deficiency and blood stasis syndrome by gut microbiota combined with metabolomics
Source: Chin Med. 2023 Apr 23;18:44. doi: 10.1186/s13020-023-00734-8 (PMC10122815; doi:10.1186/s13020-023-00734-8)

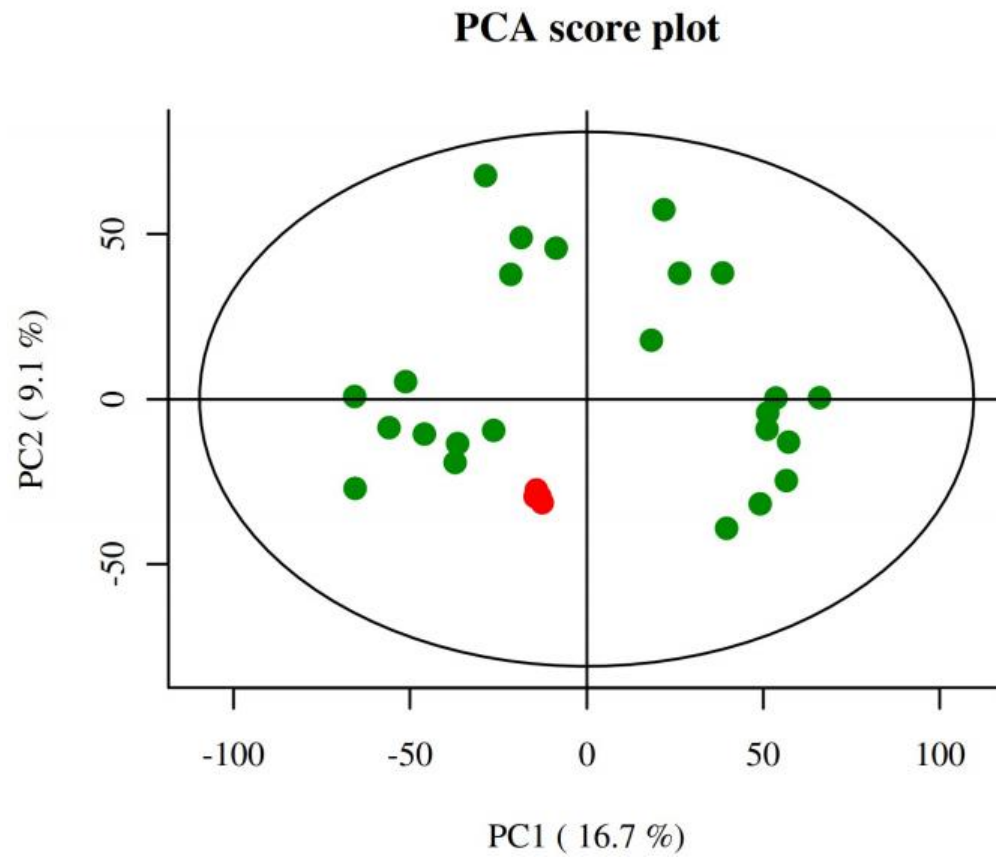

Supplementary Figure S2 PCA score chart of QC samples

Supplement: Supplementary file 1 — Additional file 1. PCA score chart of QC samples. [file 13020_2023_734_MOESM1_ESM.pdf]

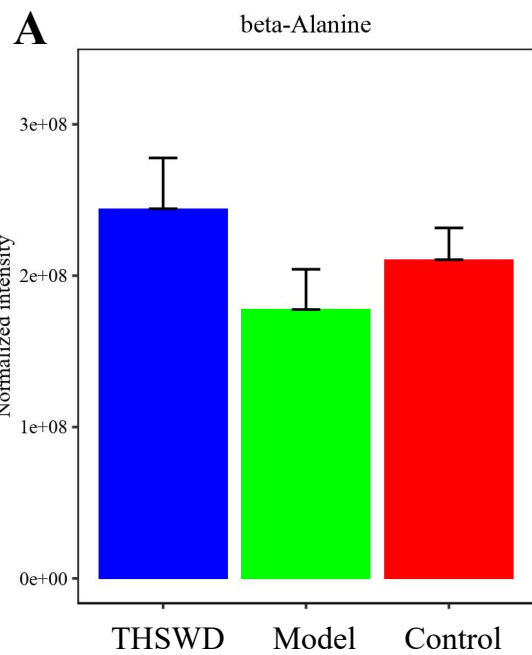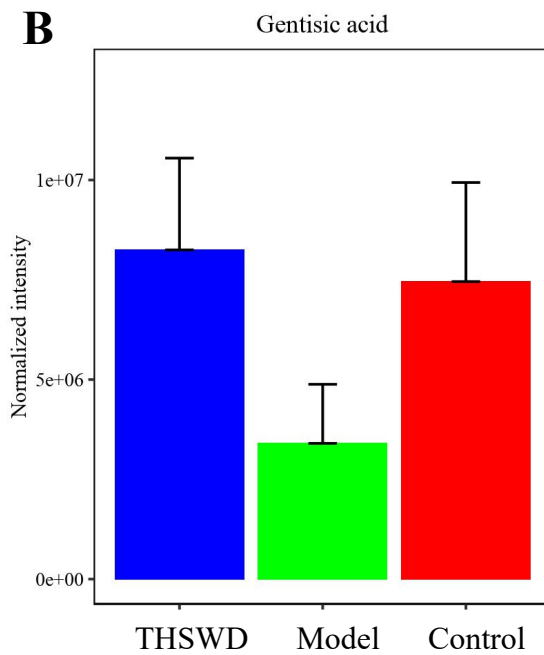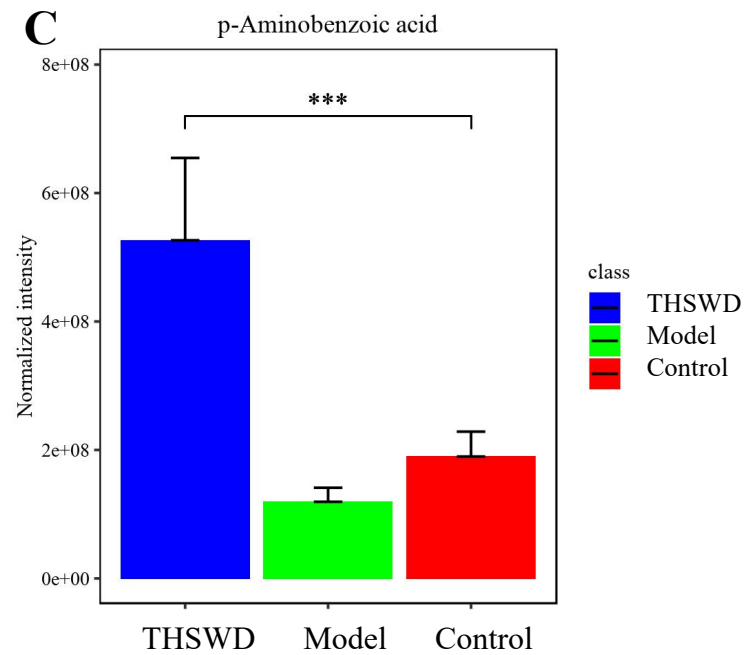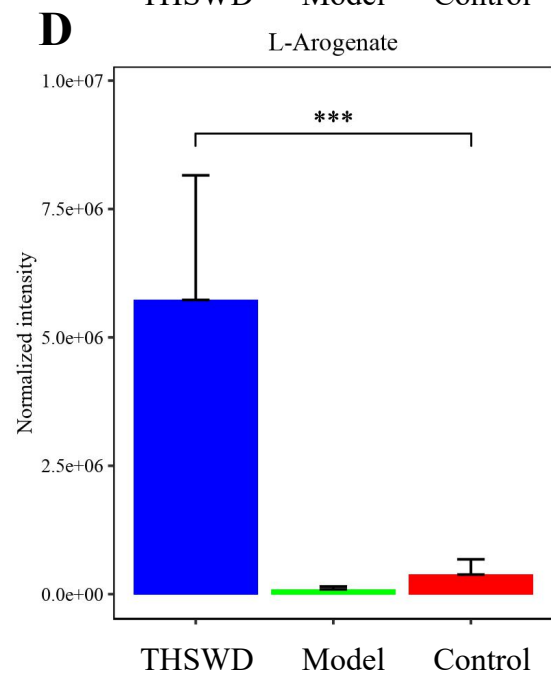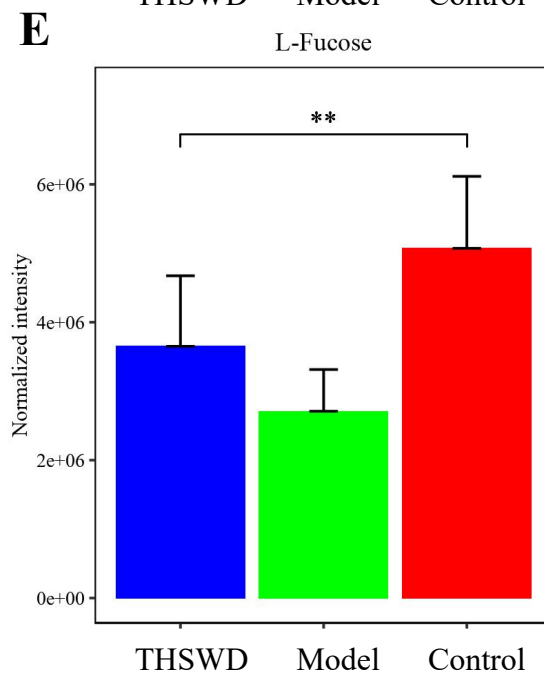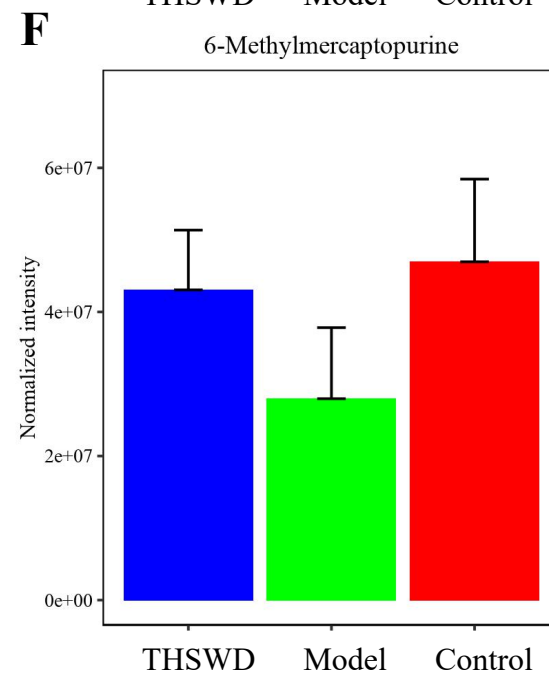

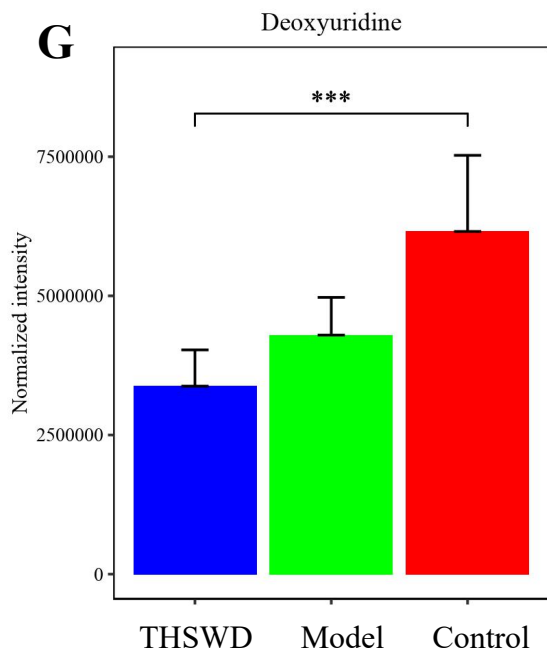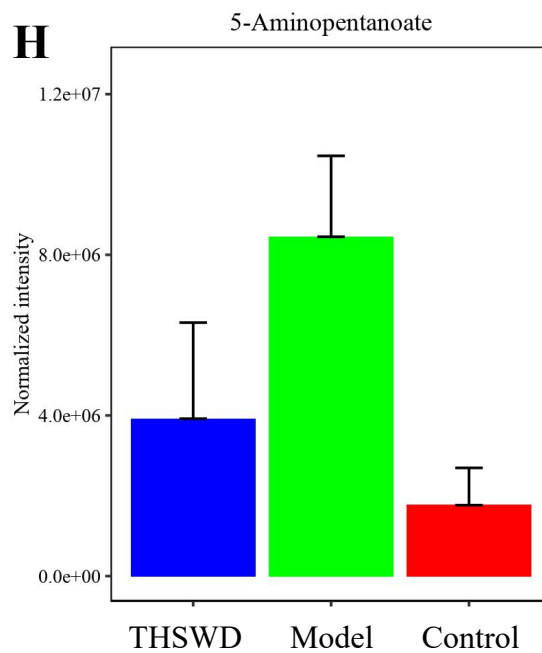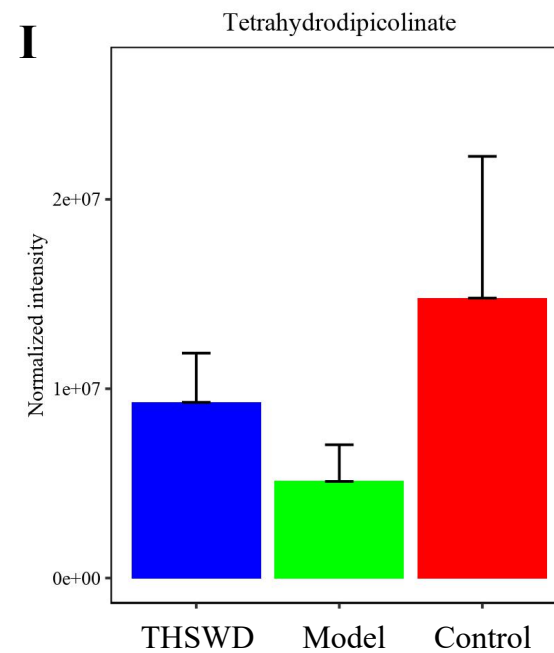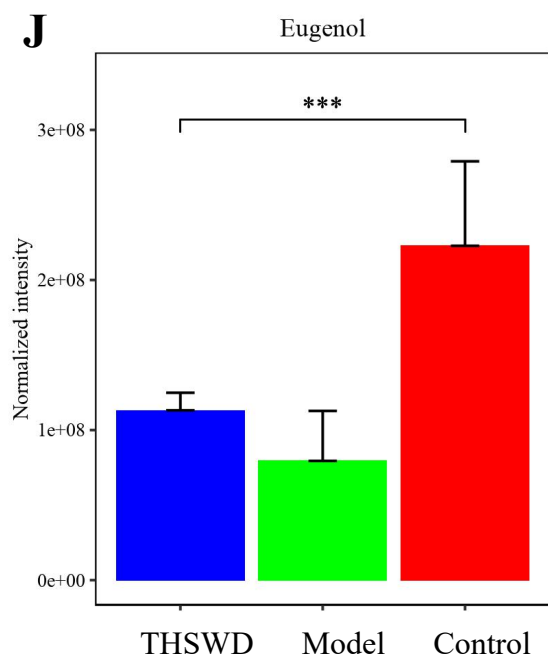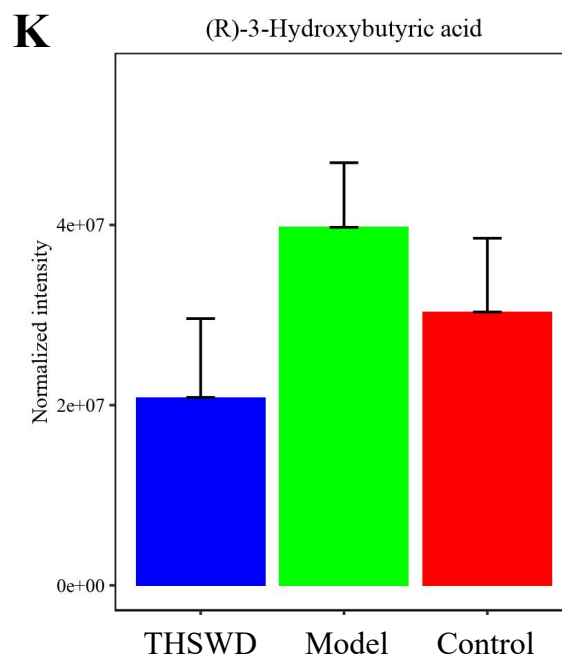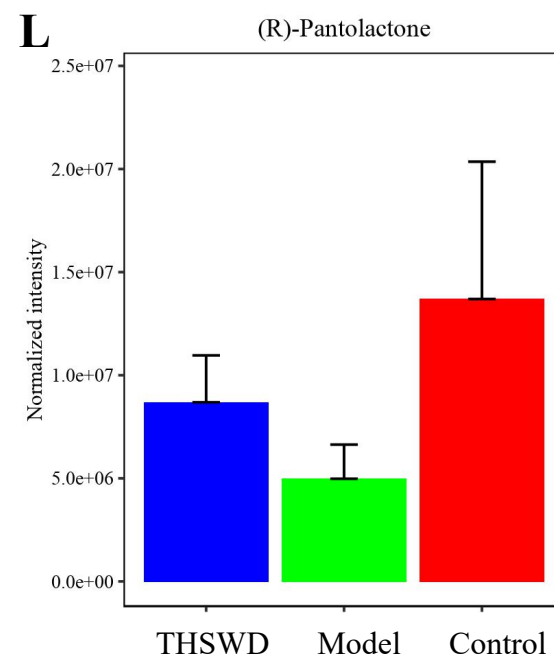

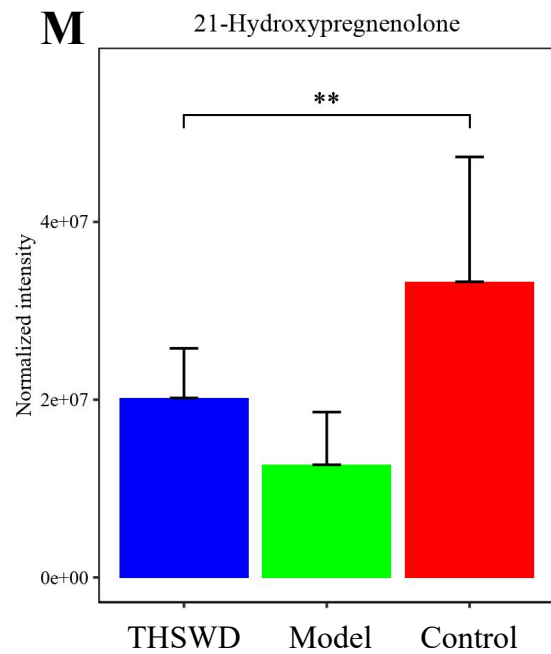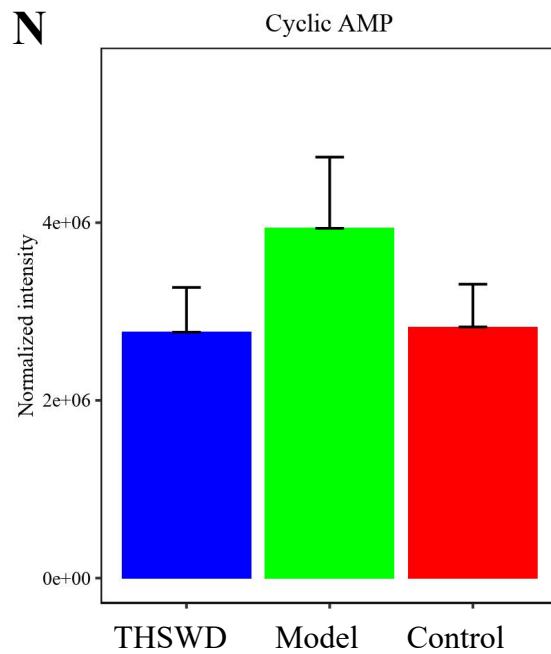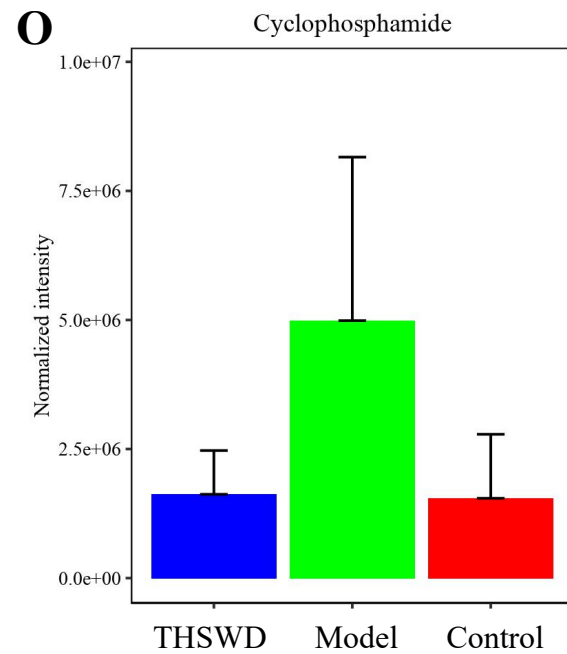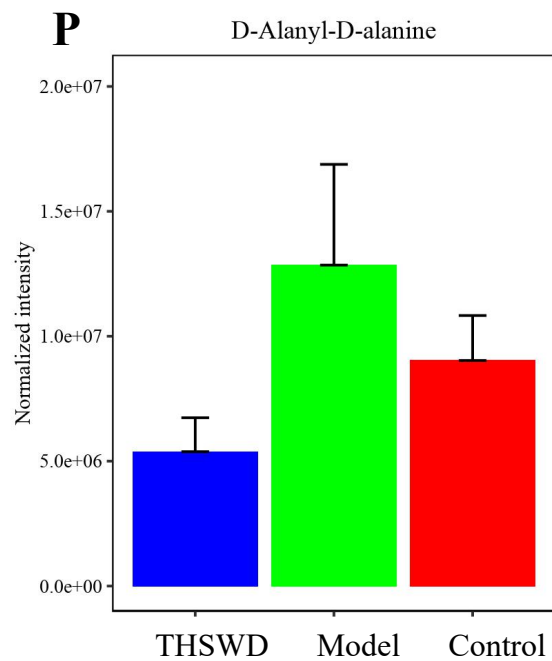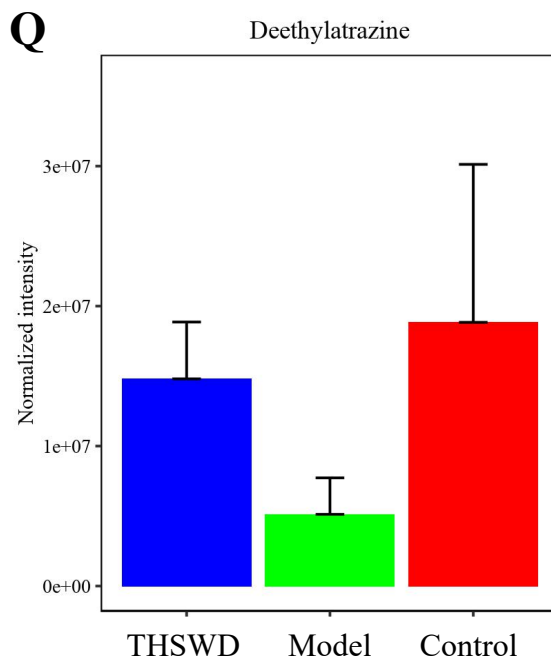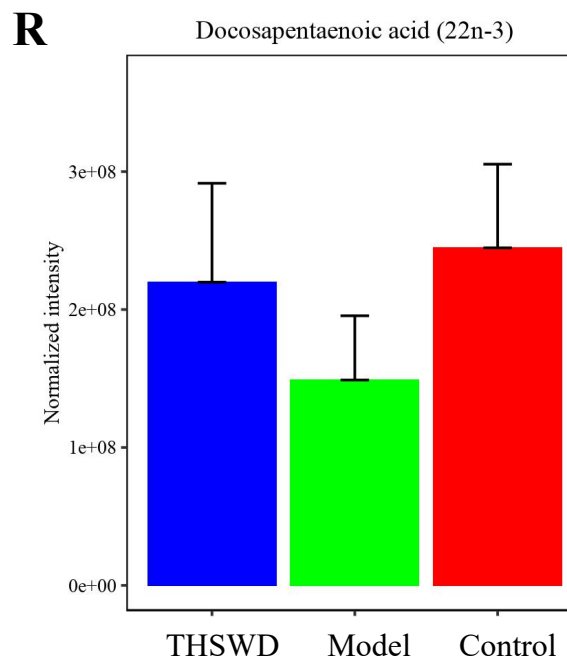

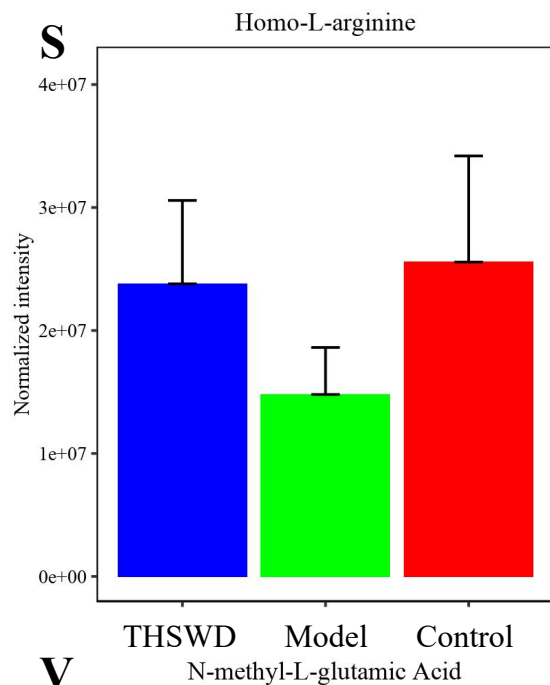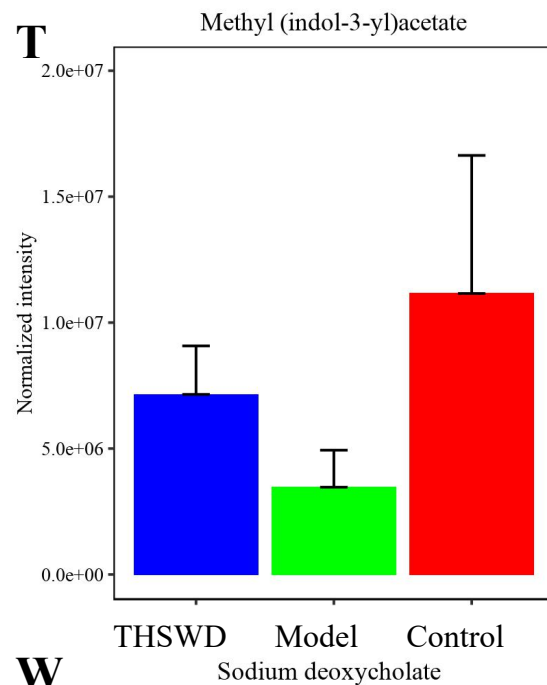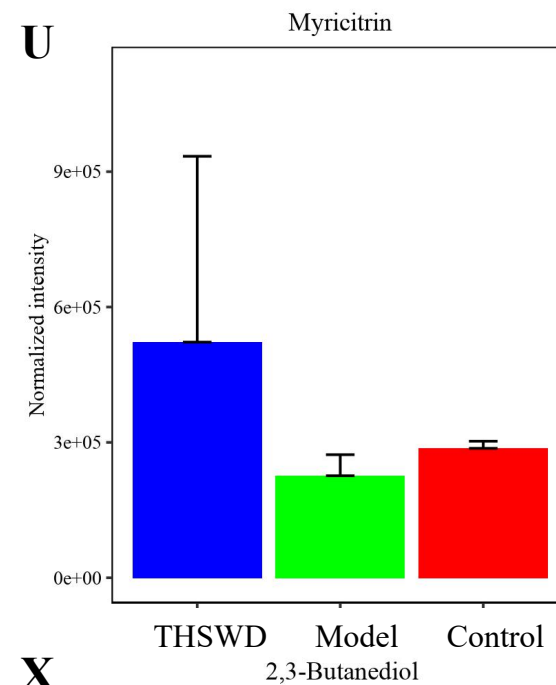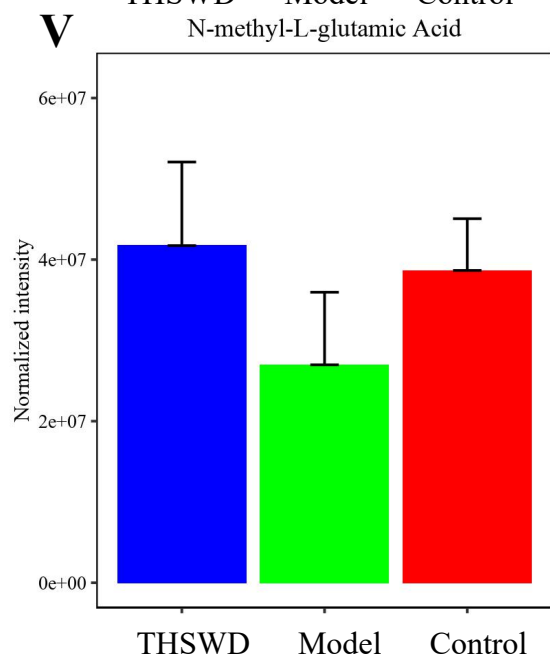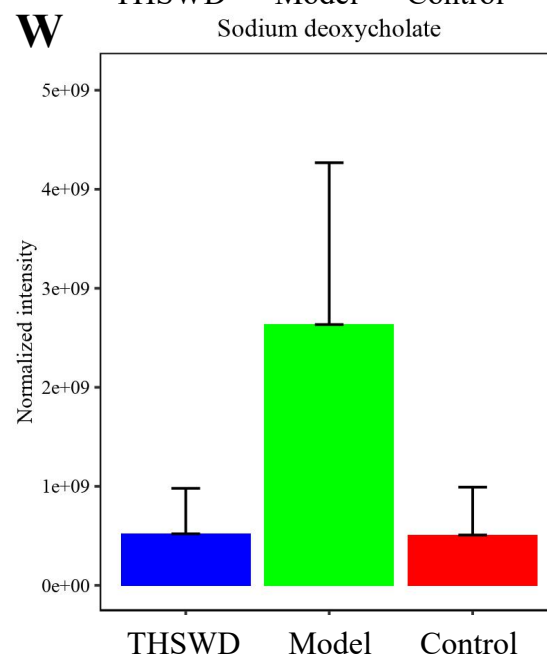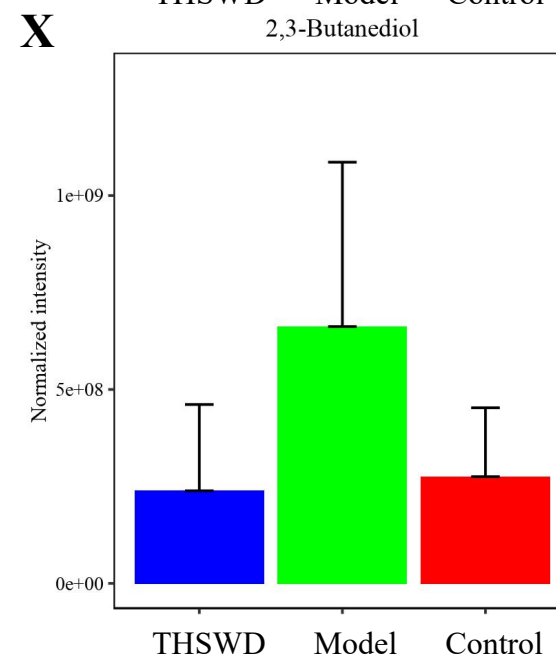

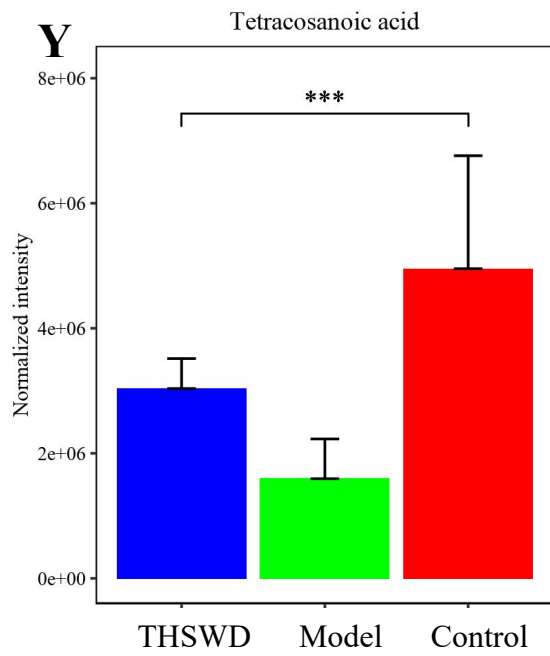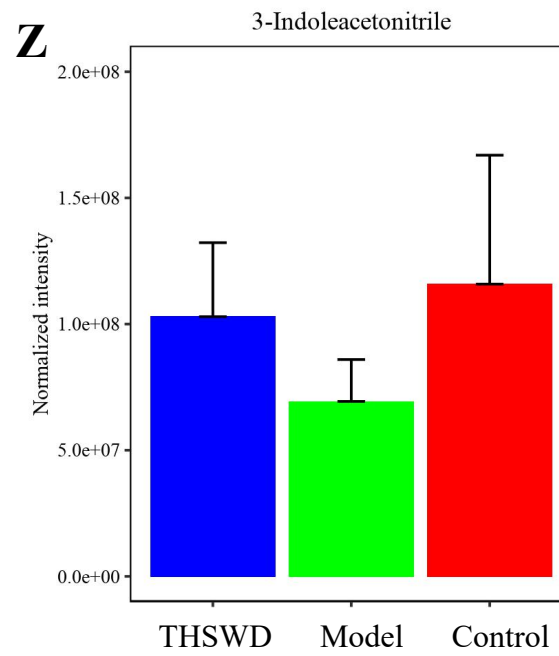

Supplement: Supplementary file 3 — Additional file 3. Histogram of differential metabolites for each group. [file 13020_2023_734_MOESM3_ESM.pdf]

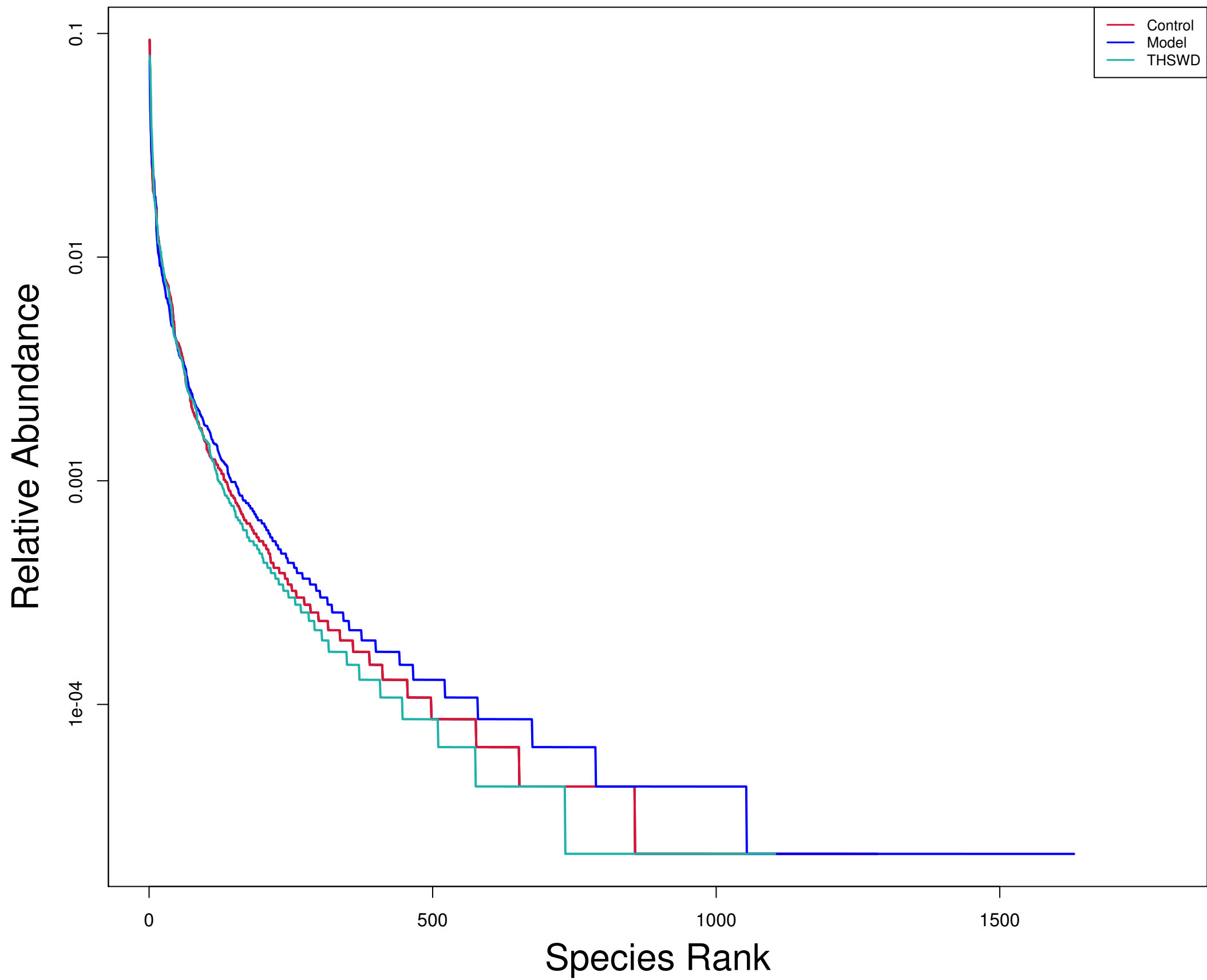

Supplement: Supplementary file 4 — Additional file 4. Rank Abundance Curve. [file 13020_2023_734_MOESM4_ESM.pdf]

Observed species

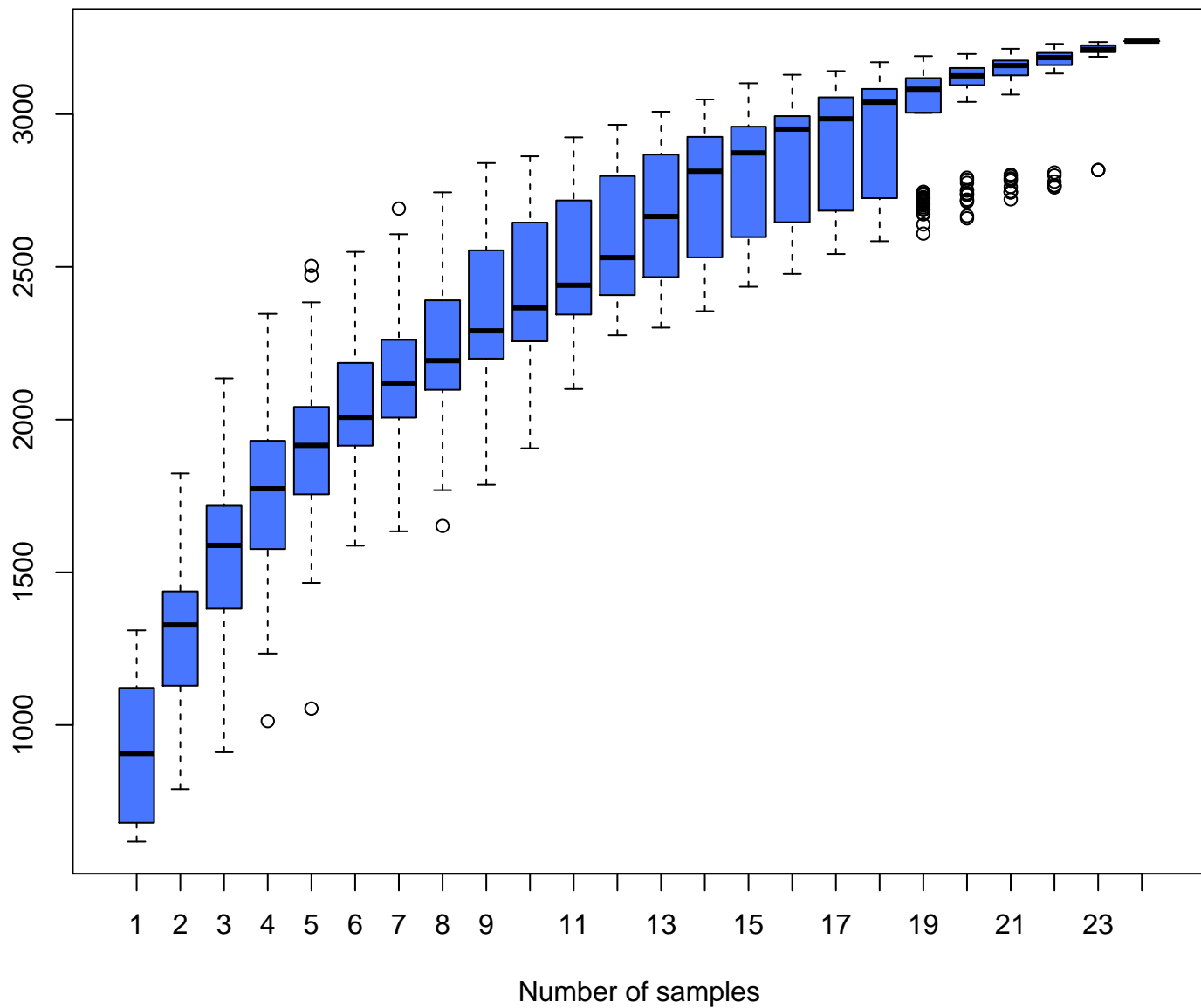

Supplement: Supplementary file 5 — Additional file 5. Species accumulation boxplot. [file 13020_2023_734_MOESM5_ESM.pdf]
