# Supplementary material for: Exploring the mechanism of Taohong Siwu Decoction on the treatment of blood deficiency and blood stasis syndrome by gut microbiota combined with metabolomics
Source: Chin Med. 2023 Apr 23;18:44. doi: 10.1186/s13020-023-00734-8 (PMC10122815; doi:10.1186/s13020-023-00734-8)
Supplement: Supplementary file 2 — Additional file 2. MS/MS mapping of twenty-three different metabolites. [file 13020_2023_734_MOESM2_ESM.pdf]

A

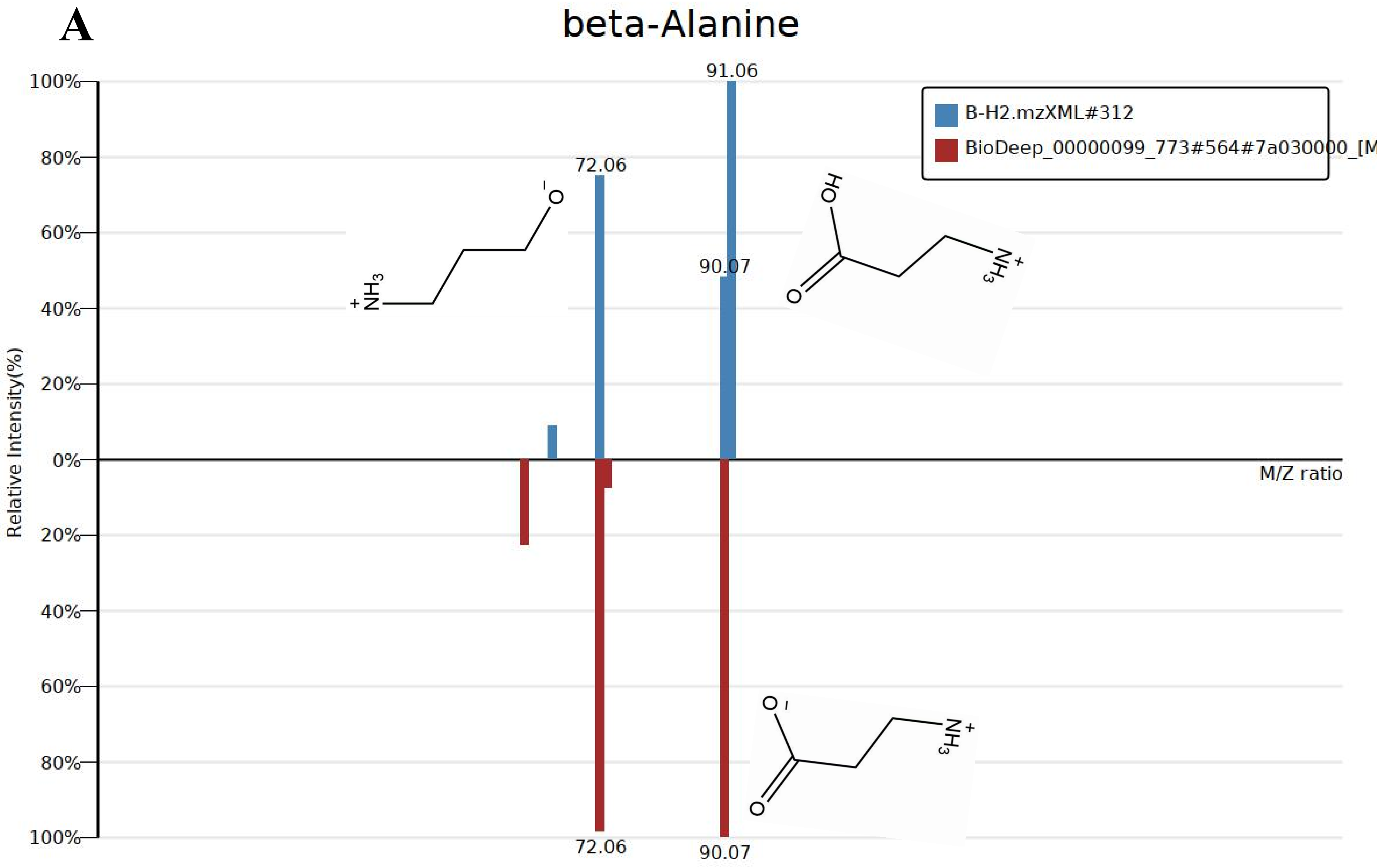

beta-Alanine
B-H2.mzXML#312
BioDeep_00000099_773#564#7a030000_[M
100%
80%
60%
40%
20%
0%
20%
40%
60%
80%
100%
Relative Intensity(%)
M/Z ratio
91.06
72.06
90.07
72.06
90.07

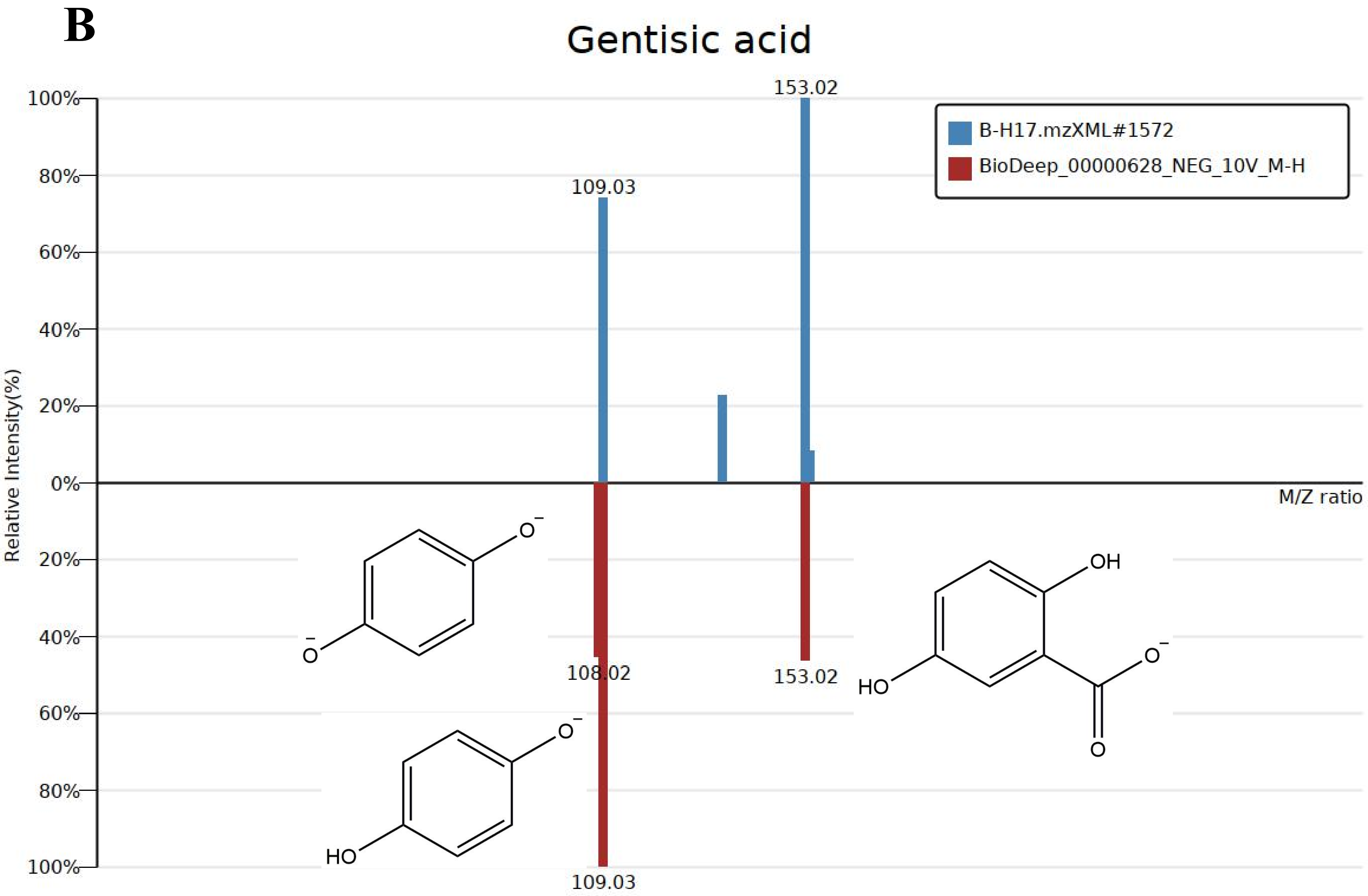

B
Gentisic acid
B-H17.mzXML#1572
BioDeep_00000628_NEG_10V_M-H
Relative Intensity(%)
100%
80%
60%
40%
20%
0%
20%
40%
60%
80%
100%
M/Z ratio
153.02
109.03
108.02
153.02
109.03
O
O
OH
HO
O
O
HO
O

C

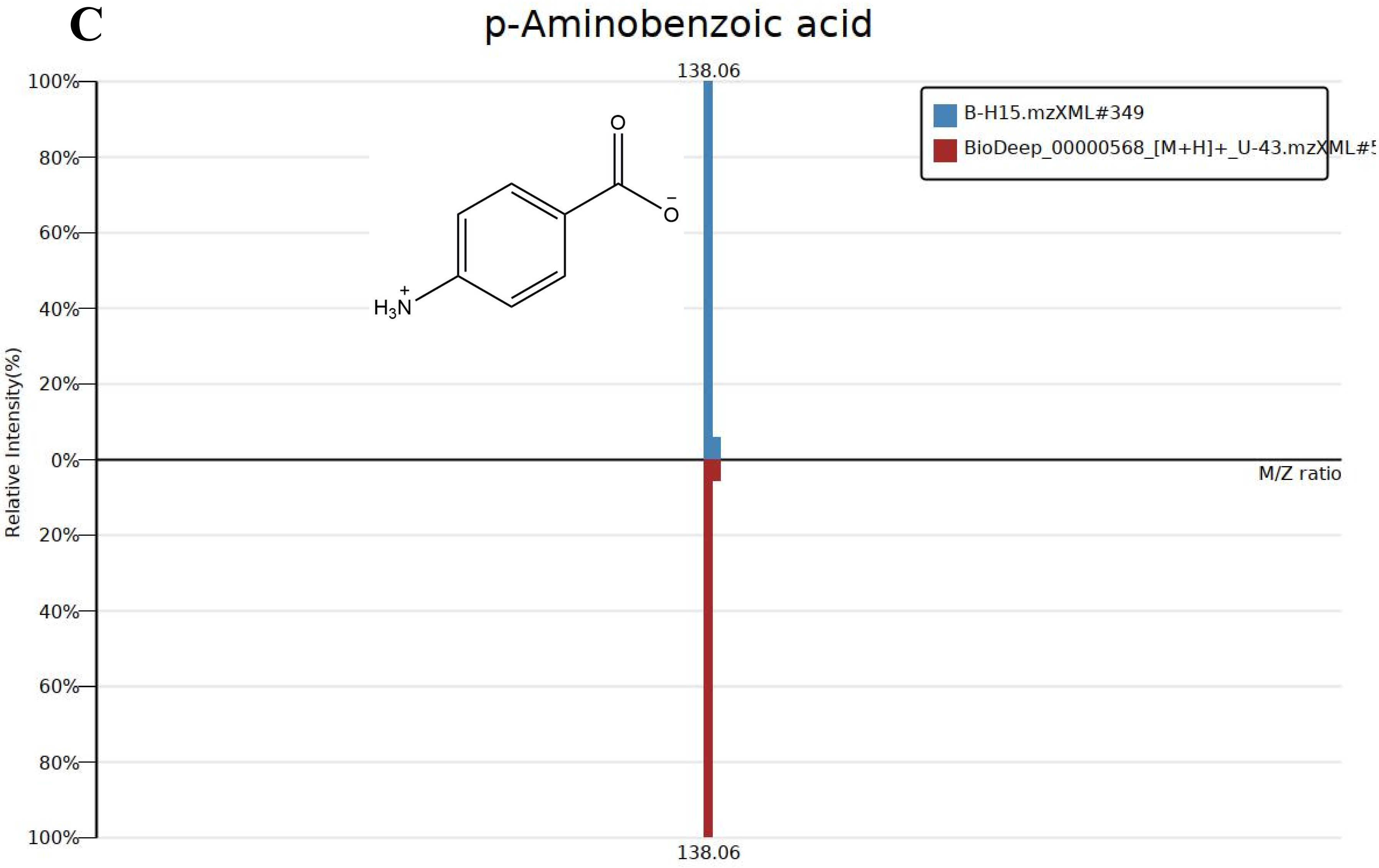
p-Aminobenzoic acid
138.06
B-H15.mzXML#349
BioDeep_00000568_[M+H]+_U-43.mzXML#5
100%
80%
60%
40%
20%
0%
20%
40%
60%
80%
100%
Relative Intensity(%)
M/Z ratio
138.06

D

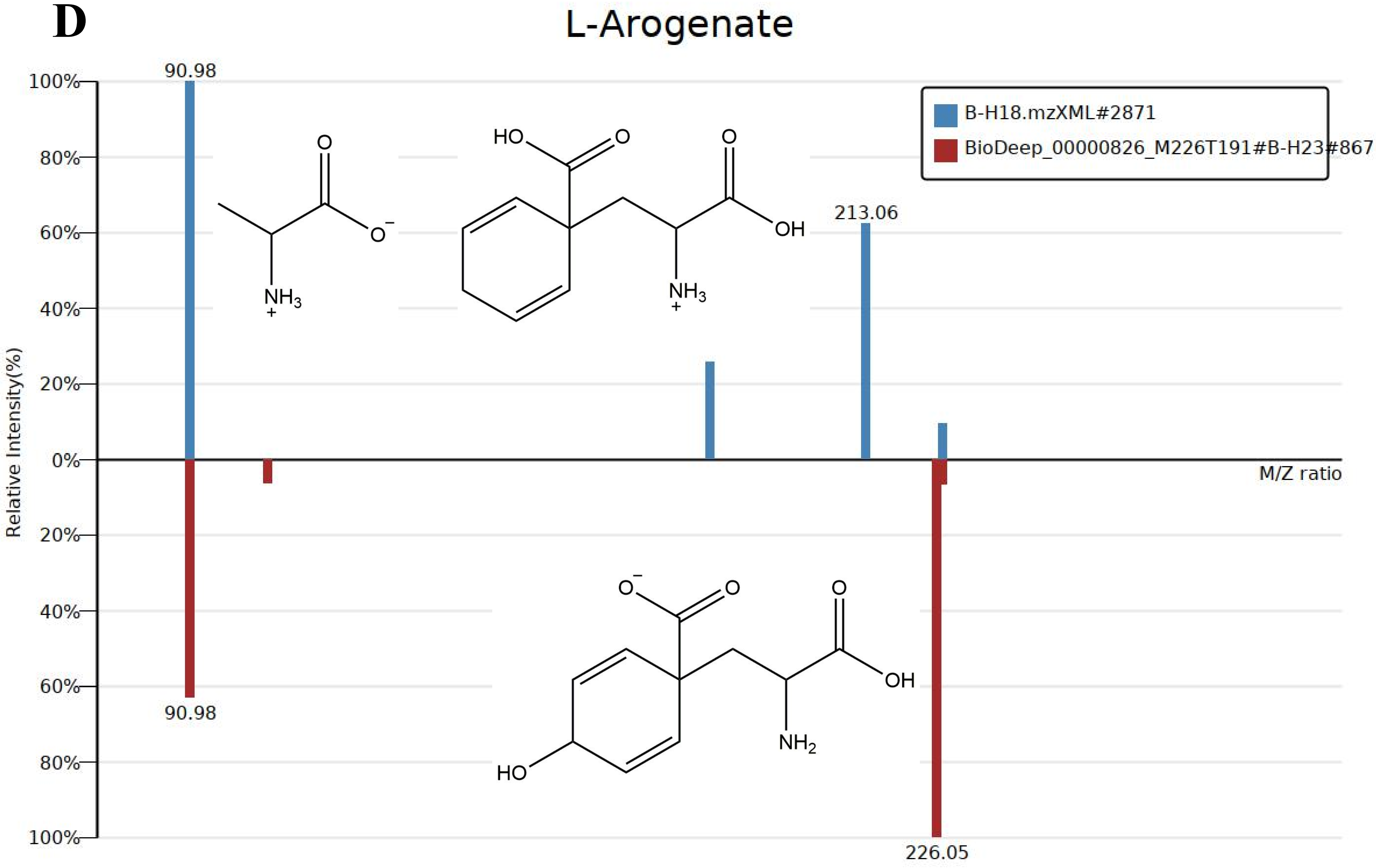
L-Arogenate
B-H18.mzXML#2871
BioDeep_00000826_M226T191#B-H23#867
90.98
213.06
90.98
226.05
Relative Intensity(%)
M/Z ratio
100%
80%
60%
40%
20%
0%
20%
40%
60%
80%
100%

E

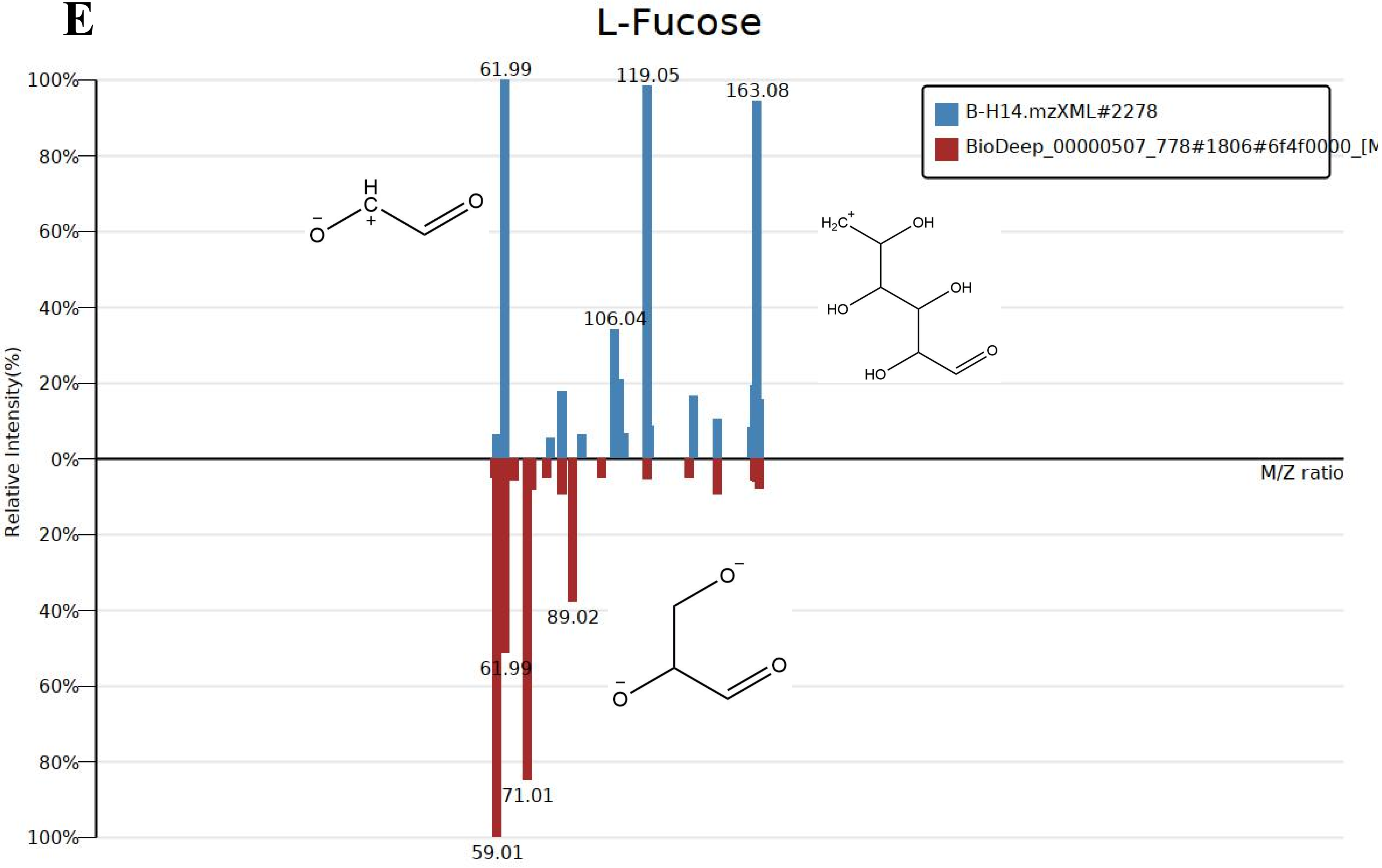
L-Fucose
B-H14.mzXML#2278
BioDeep_00000507_778#1806#6f4f0000_[M
Relative Intensity(%)
M/Z ratio
100%
80%
60%
40%
20%
0%
20%
40%
60%
80%
100%
61.99
119.05
163.08
106.04
89.02
61.99
71.01
59.01

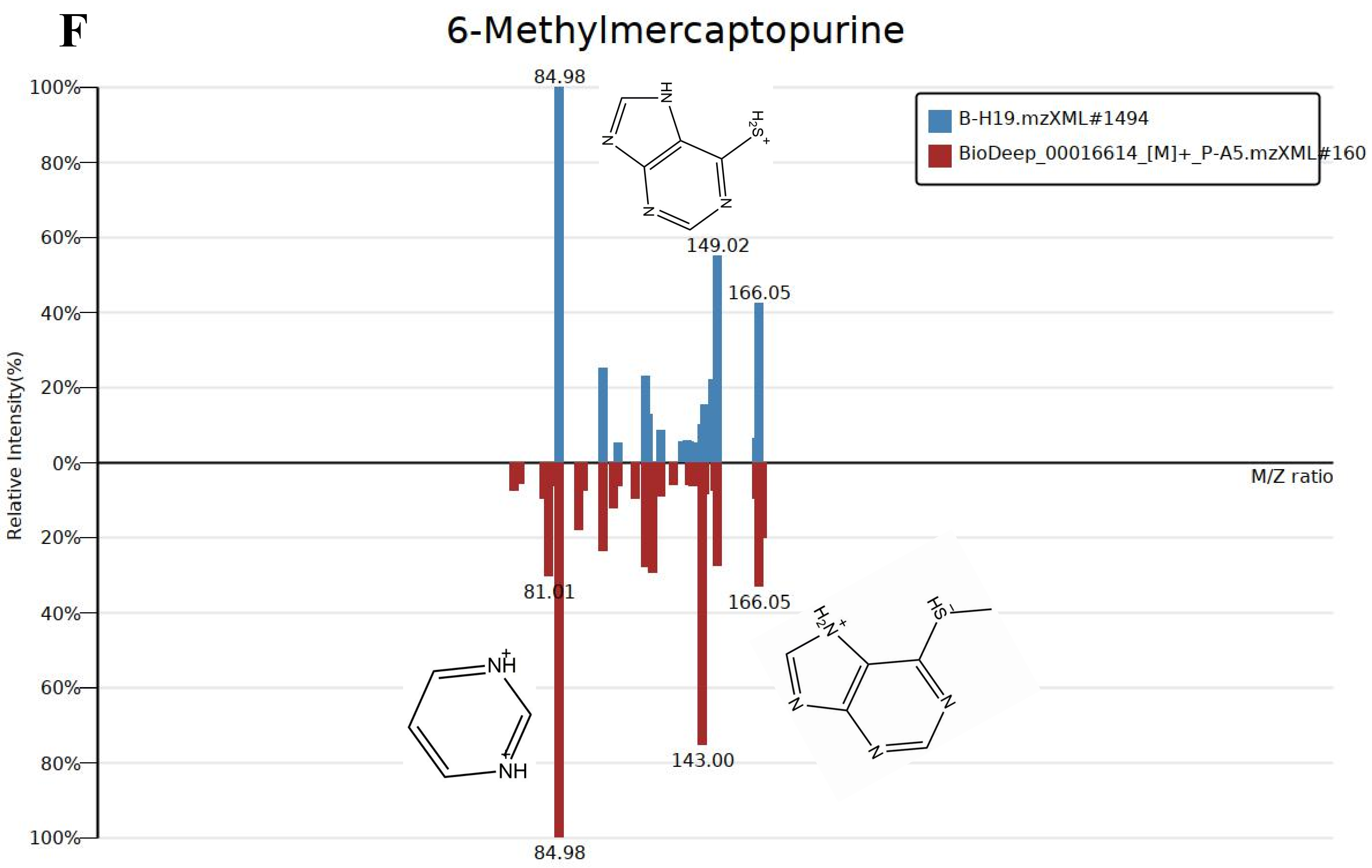

F
6-Methylmercaptopurine
B-H19.mzXML#1494
BioDeep_00016614_[M]+_P-A5.mzXML#160
Relative Intensity(%)
M/Z ratio
100%
80%
60%
40%
20%
0%
20%
40%
60%
80%
100%
84.98
149.02
166.05
81.01
166.05
143.00
84.98

G

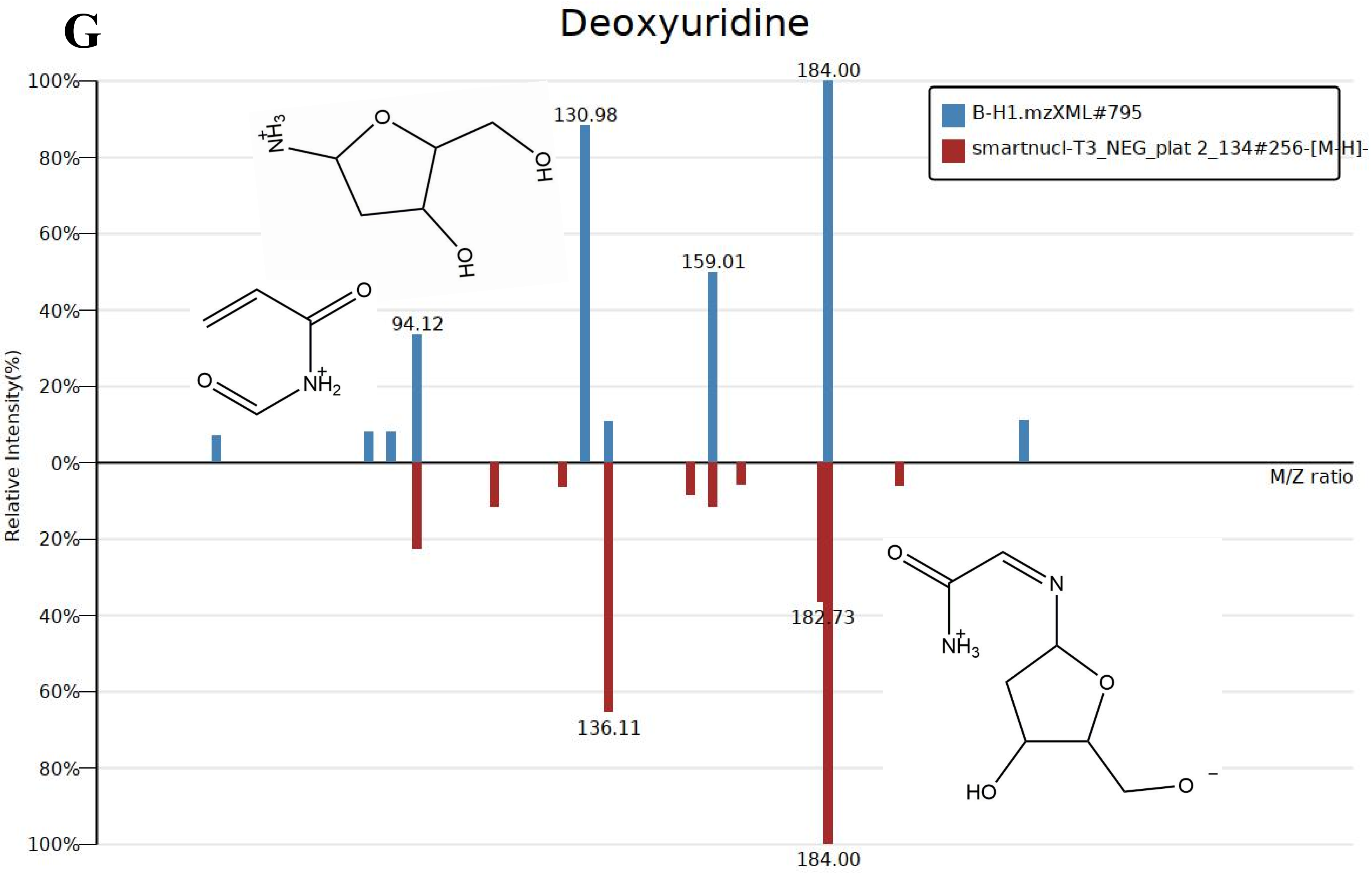
Deoxyuridine
B-H1.mzXML#795
smartnucl-T3_NEG_plat 2_134#256-[M-H]-
Relative Intensity(%)
M/Z ratio
100%
80%
60%
40%
20%
0%
184.00
130.98
159.01
94.12
136.11
182.73
184.00

H

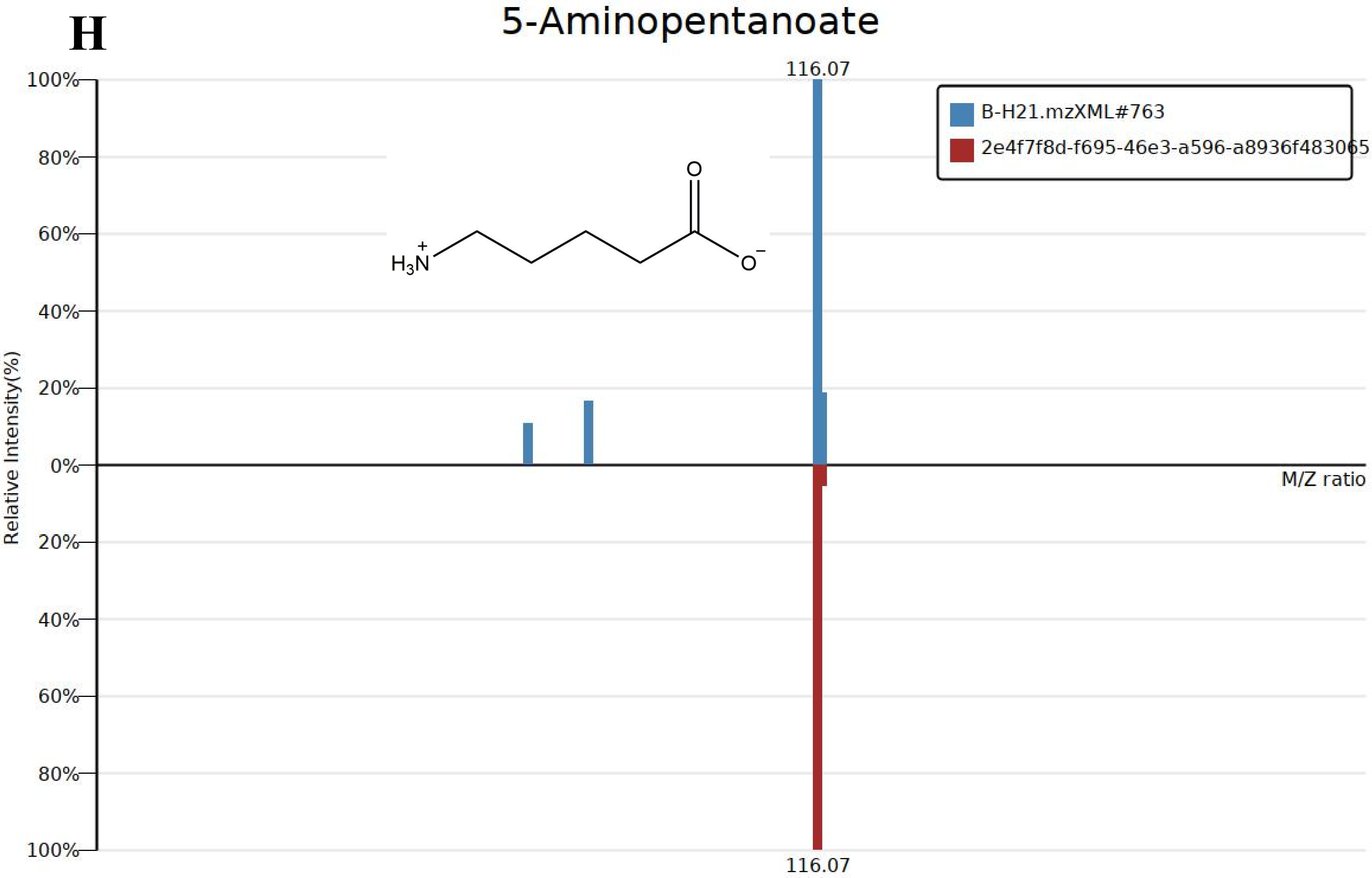
5-Aminopentanoate
116.07
100%
80%
60%
40%
20%
0%
20%
40%
60%
80%
100%
Relative Intensity(%)
B-H21.mzXML#763
2e4f7f8d-f695-46e3-a596-a8936f483065
O
H_3N^+
O^-
M/Z ratio
116.07

I

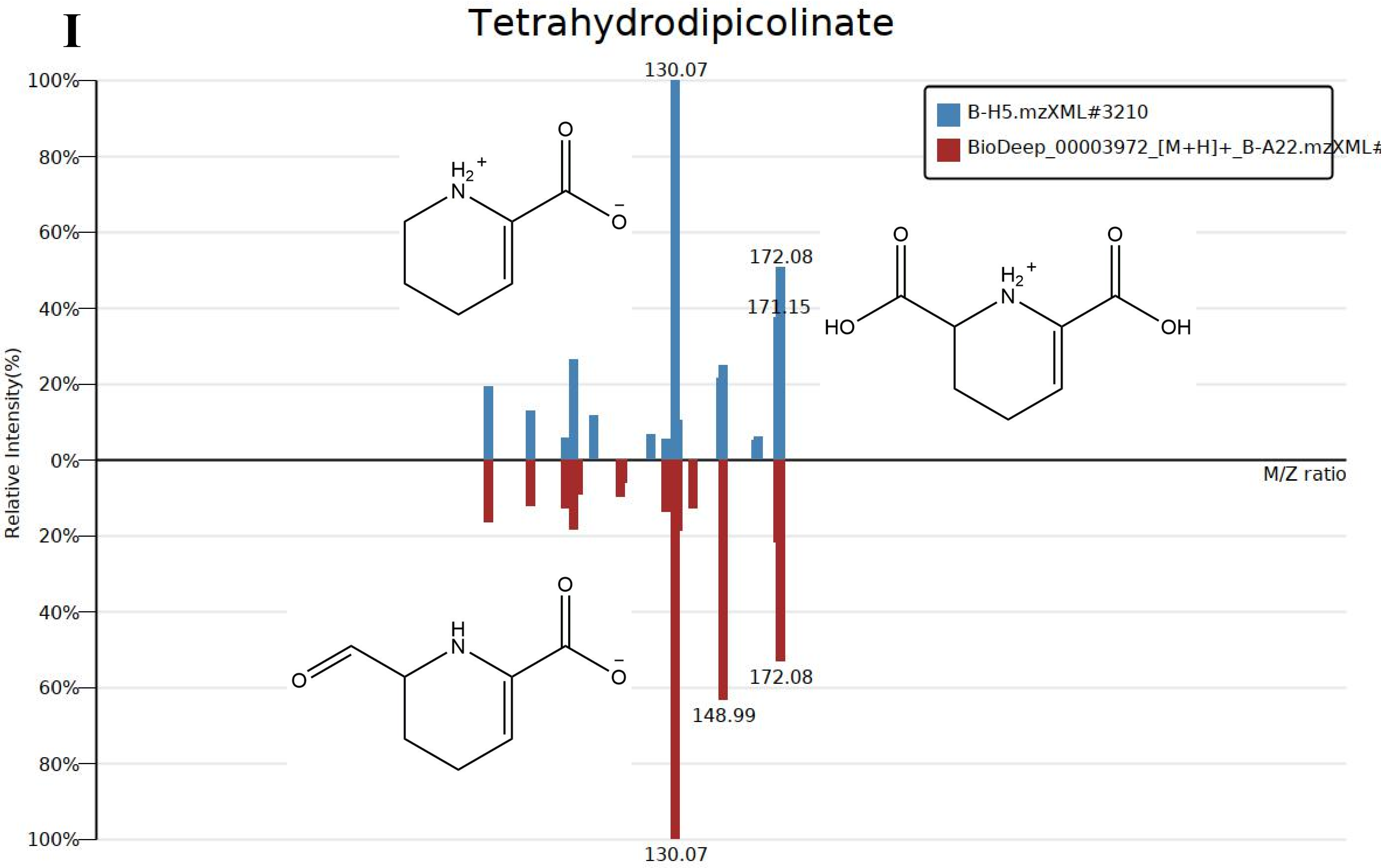

Tetrahydrodipicolinate
B-H5.mzXML#3210
BioDeep_00003972_[M+H]+_B-A22.mzXML#
130.07
172.08
171.15
148.99
172.08
130.07
100%
80%
60%
40%
20%
0%
20%
40%
60%
80%
100%
Relative Intensity(%)
M/Z ratio

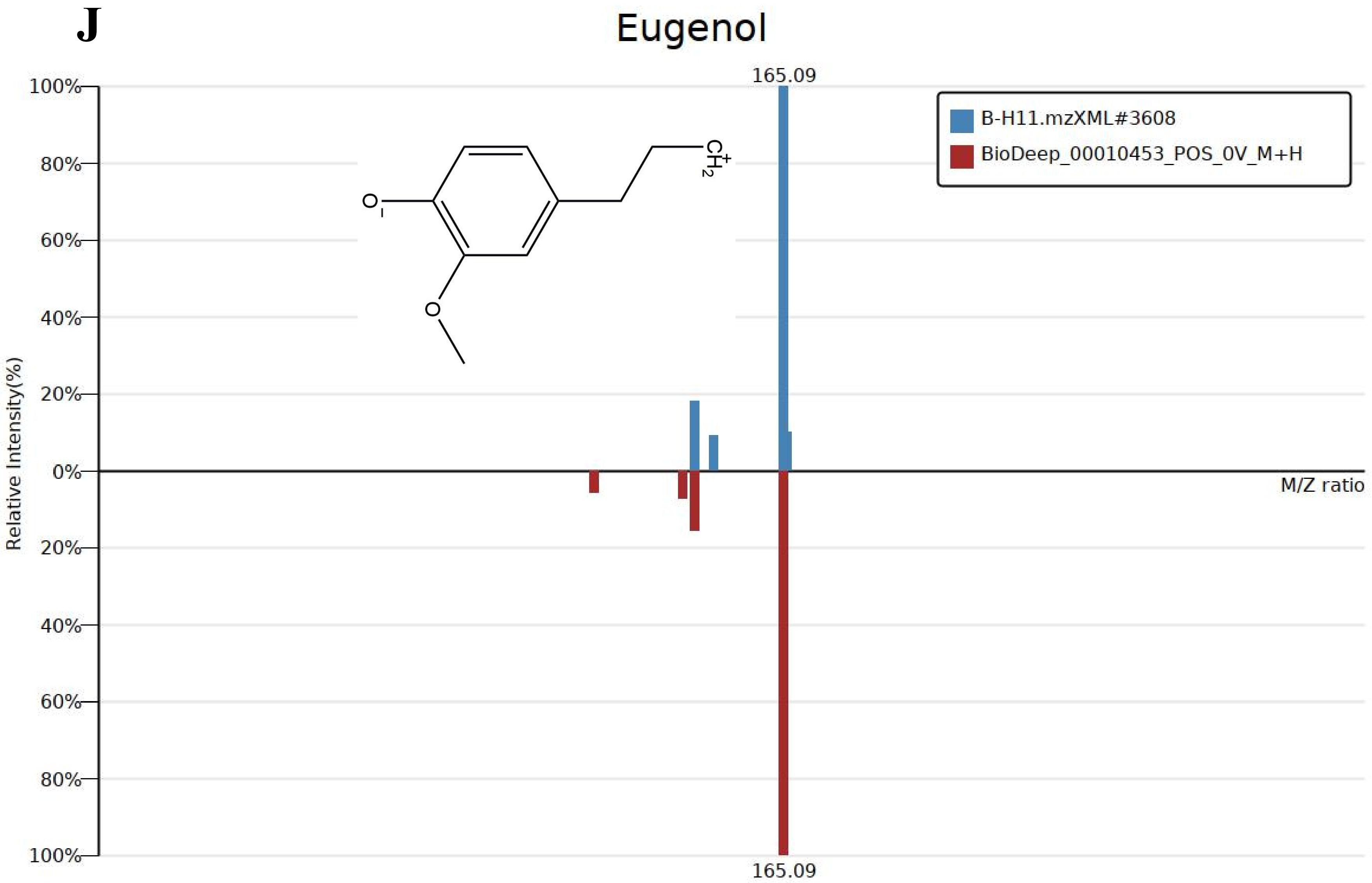
J
Eugenol
165.09
100%
80%
60%
40%
20%
0%
20%
40%
60%
80%
100%
Relative Intensity(%)
M/Z ratio
165.09
B-H11.mzXML#3608
BioDeep_00010453_POS_0V_M+H
O
O
CH2+

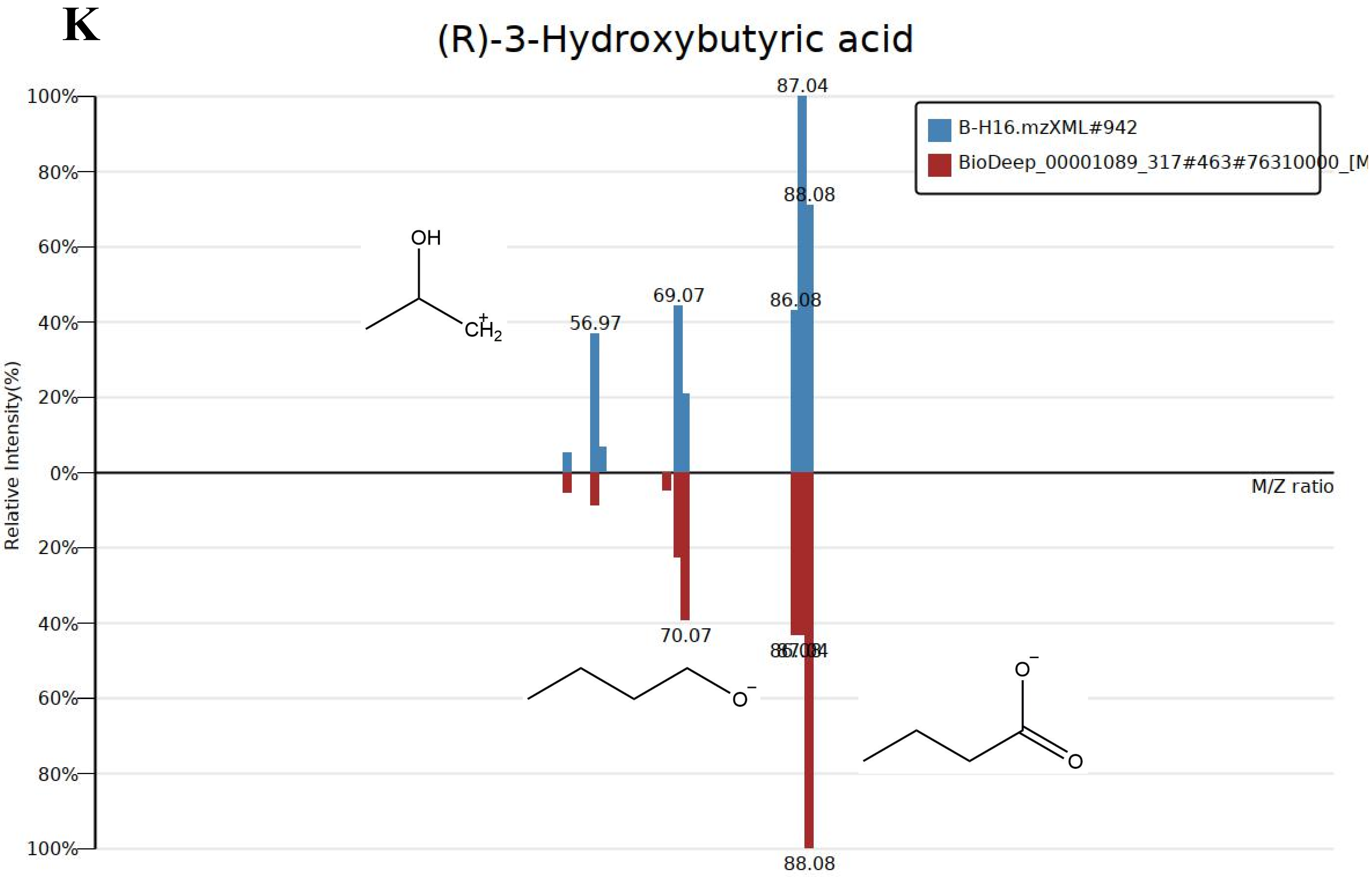
K
(R)-3-Hydroxybutyric acid
B-H16.mzXML#942
BioDeep_00001089_317#463#76310000_[M
Relative Intensity(%)
M/Z ratio
100%
80%
60%
40%
20%
0%
20%
40%
60%
80%
100%
87.04
88.08
86.08
69.07
56.97
70.07
88.08
OH
CH2
O
O
O

L

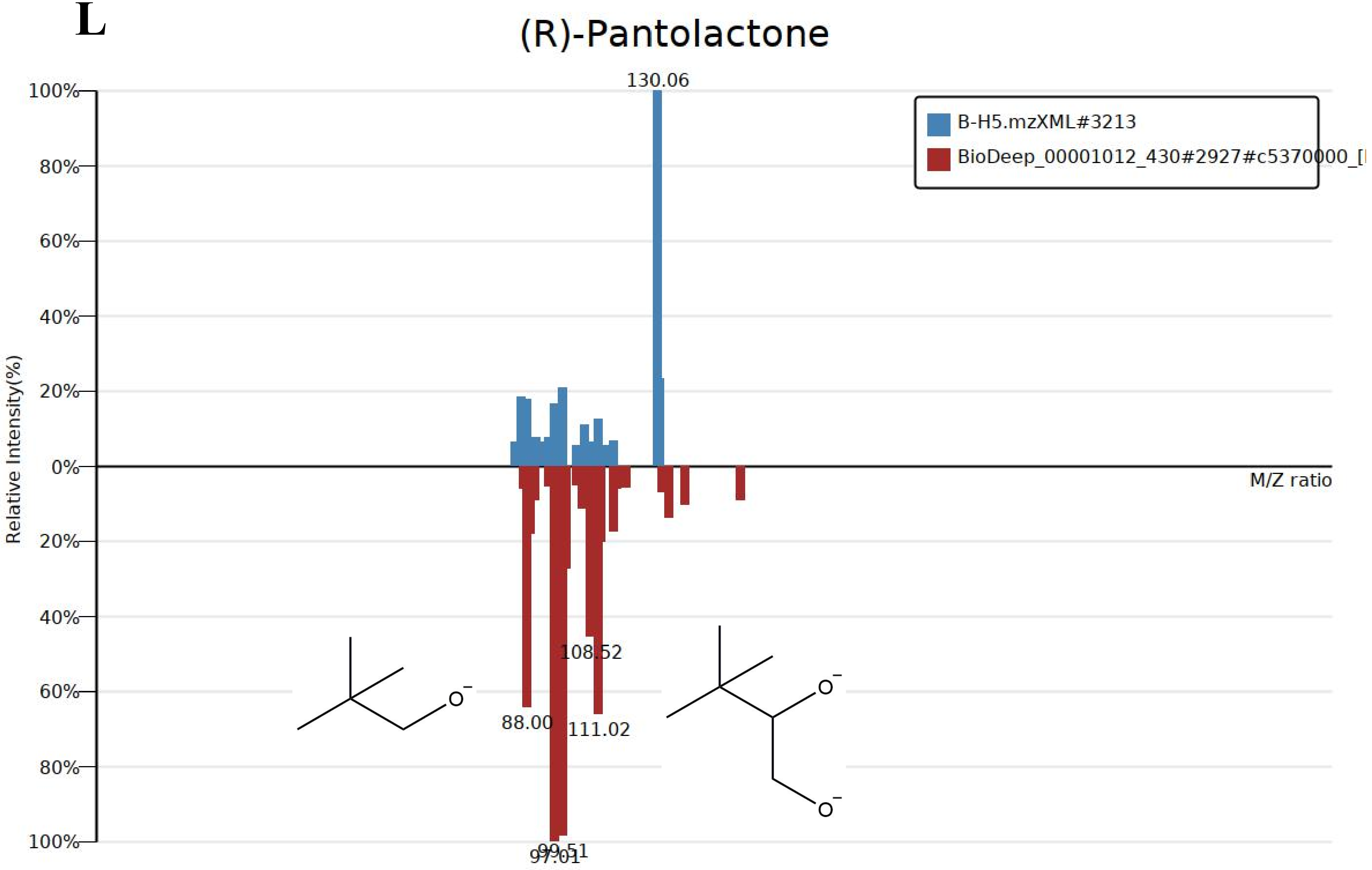

(R)-Pantolactone
130.06
B-H5.mzXML#3213
BioDeep_00001012_430#2927#c5370000_[
100%
80%
60%
40%
20%
0%
20%
40%
60%
80%
100%
Relative Intensity(%)
M/Z ratio
108.52
88.00
111.02
99.51
97.01
O⁻
O⁻
O⁻

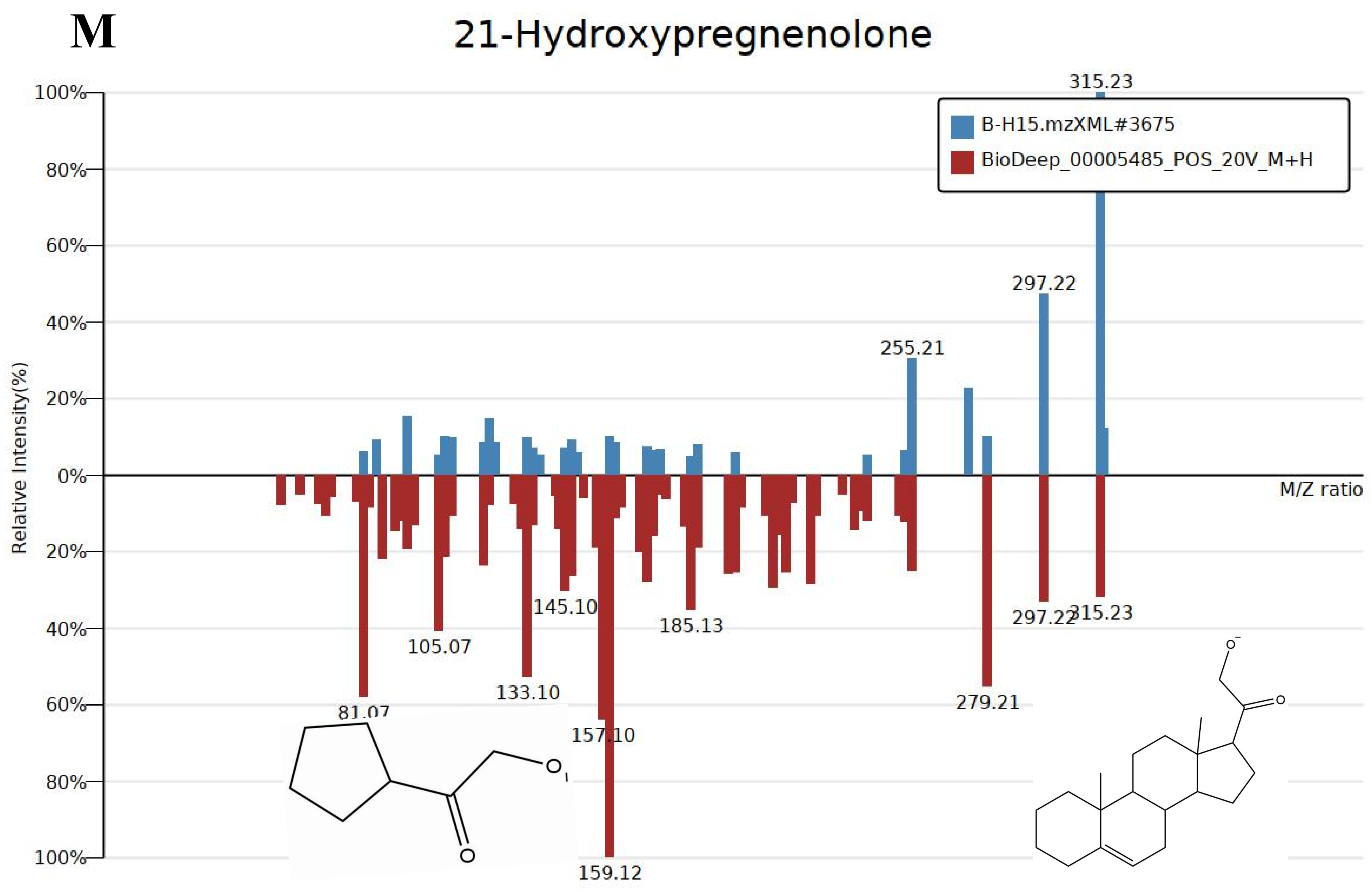
M
21-Hydroxypregnenolone
B-H15.mzXML#3675
BioDeep_00005485_POS_20V_M+H
Relative Intensity(%)
M/Z ratio
100%
80%
60%
40%
20%
0%
315.23
297.22
255.21
145.10
185.13
105.07
133.10
81.07
157.10
159.12
279.21
297.22
315.23

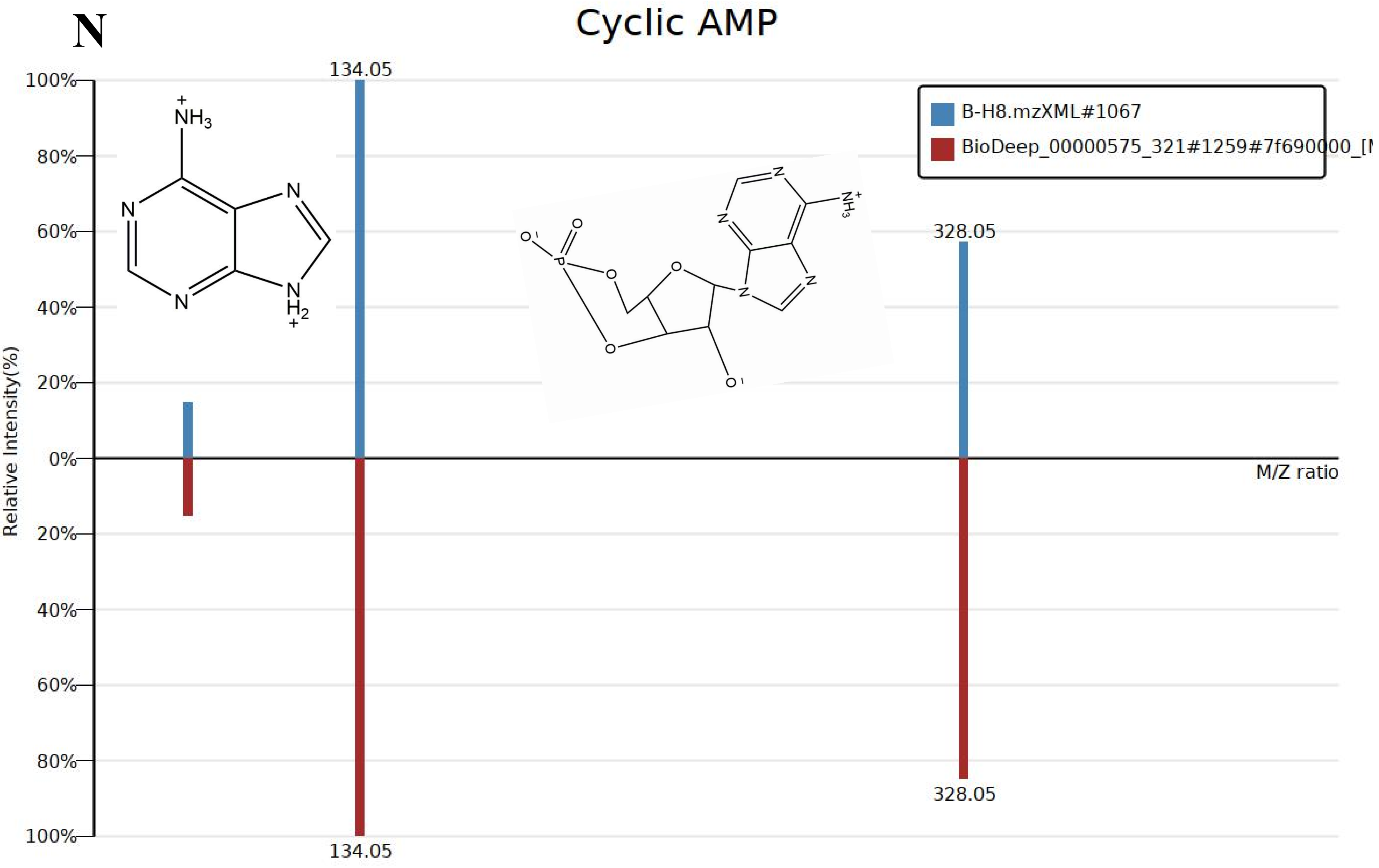
N
Cyclic AMP
134.05
328.05
B-H8.mzXML#1067
BioDeep_00000575_321#1259#7f690000_[M
100%
80%
60%
40%
20%
0%
Relative Intensity(%)
M/Z ratio
NH3
N
H2
O
P
328.05
134.05

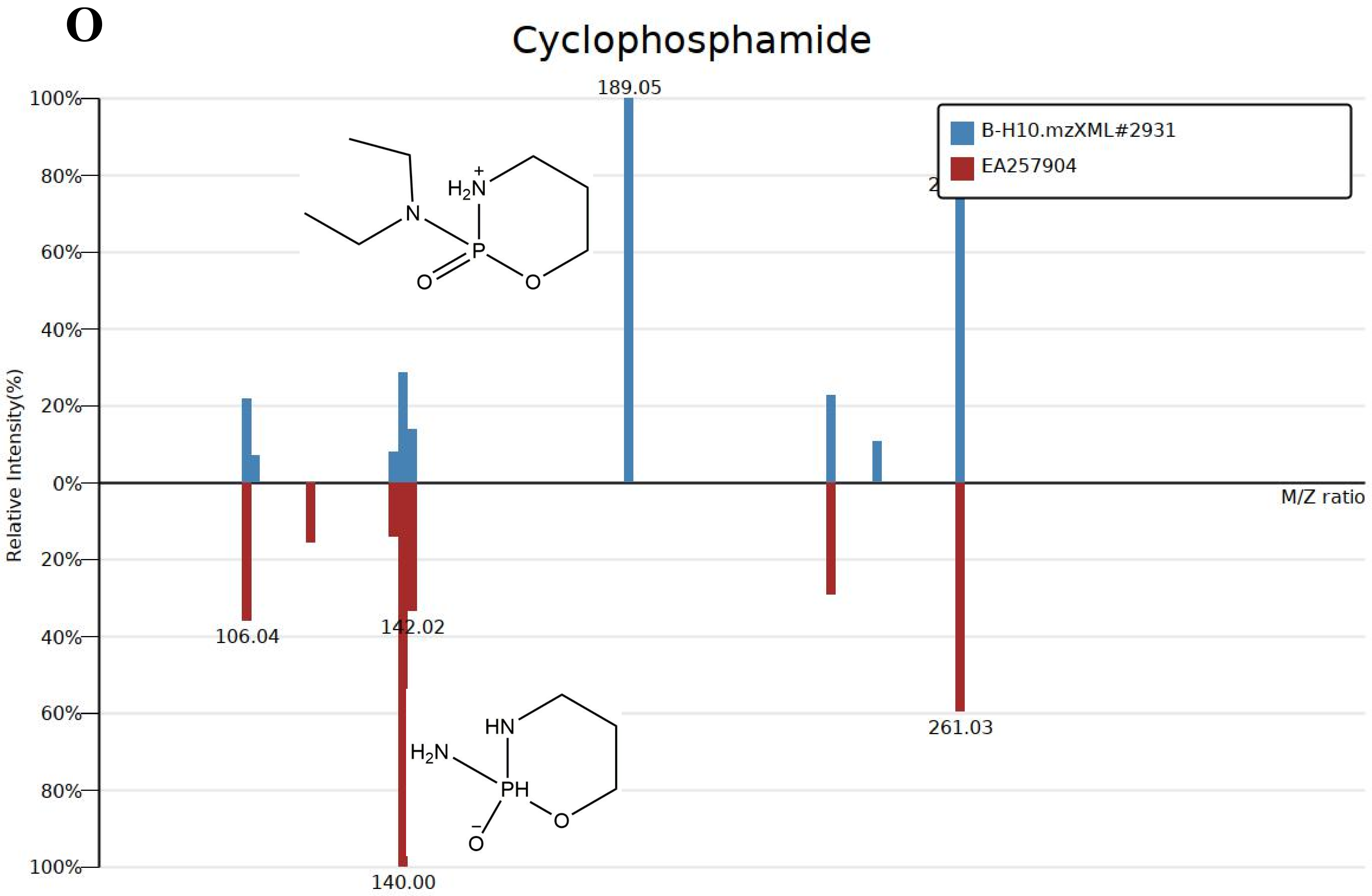
O
Cyclophosphamide
100%
80%
60%
40%
20%
0%
20%
40%
60%
80%
100%
Relative Intensity(%)
M/Z ratio
189.05
106.04
142.02
140.00
261.03
B-H10.mzXML#2931
EA257904
H2N
N
P
O
O
HN
H2N
PH
O
O

P

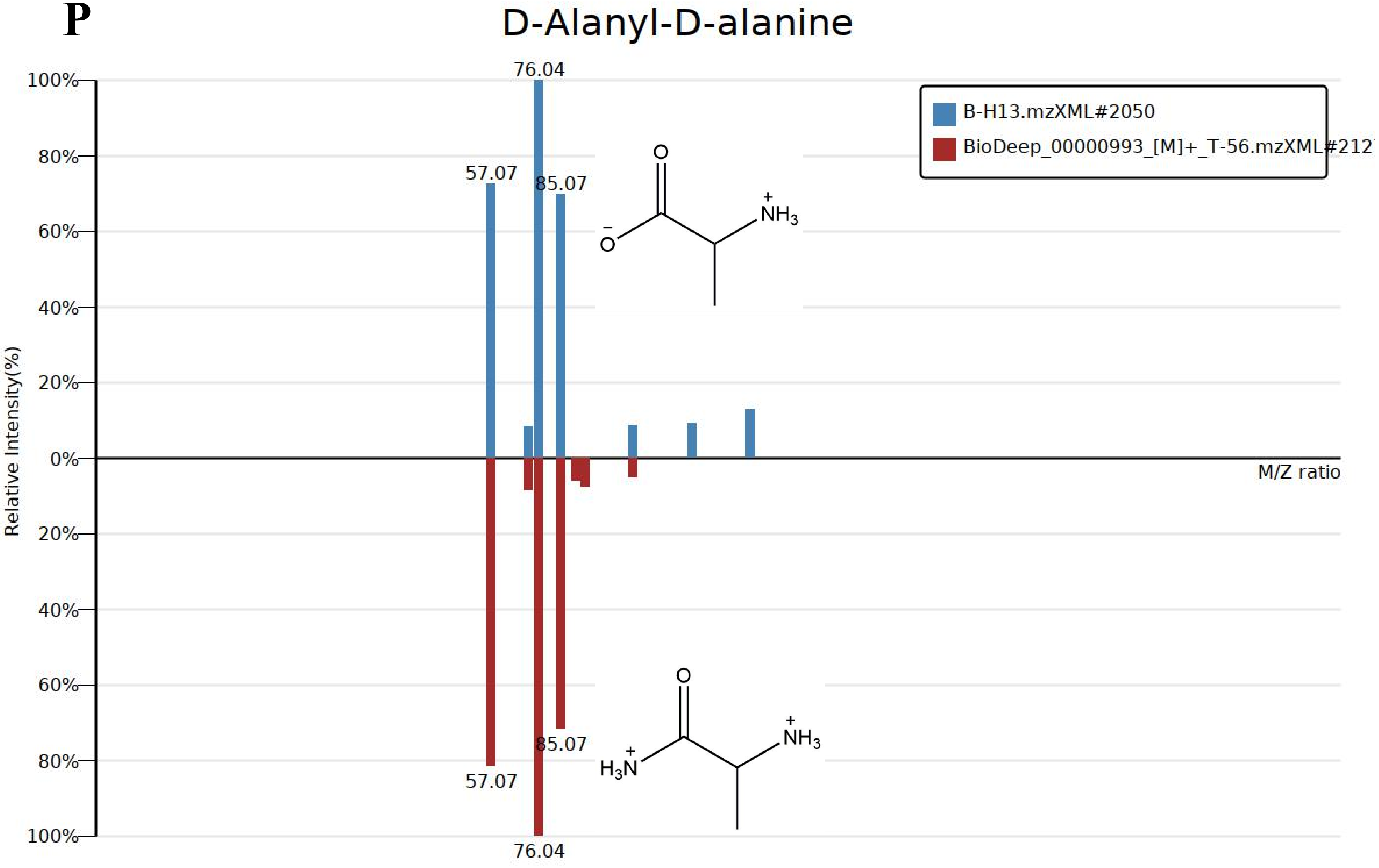
D-Alanyl-D-alanine
B-H13.mzXML#2050
BioDeep_00000993_[M]+_T-56.mzXML#212
Relative Intensity(%)
M/Z ratio
100%
80%
60%
40%
20%
0%
76.04
57.07
85.07
O
$\overset{-}{O}$
$\overset{+}{N}H_3$
$H_3\overset{+}{N}$

Q

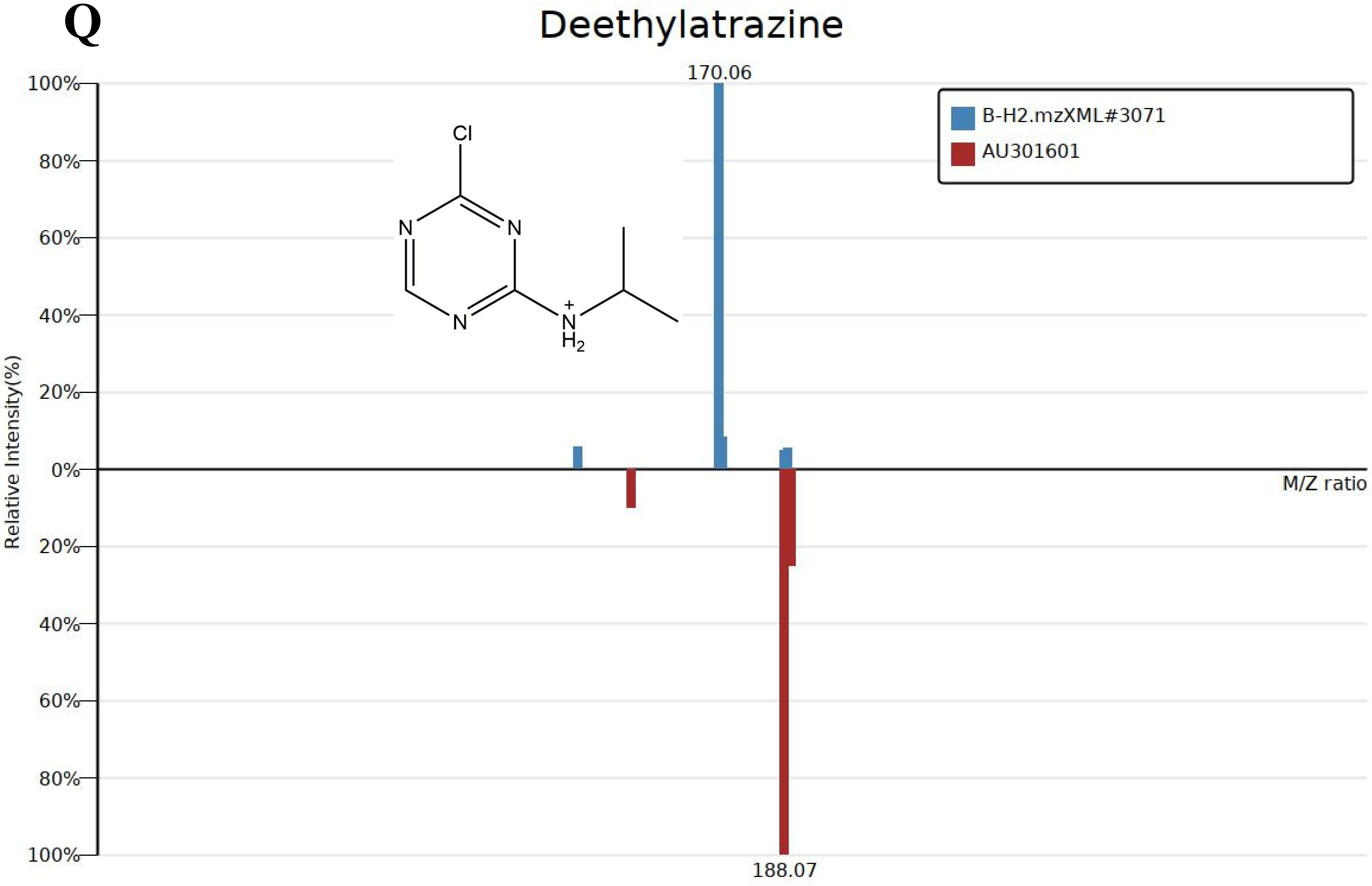
Deethylatrazine
170.06
B-H2.mzXML#3071
AU301601
Cl
N
N
N
N
H2
100%
80%
60%
40%
20%
0%
20%
40%
60%
80%
100%
Relative Intensity(%)
M/Z ratio
188.07

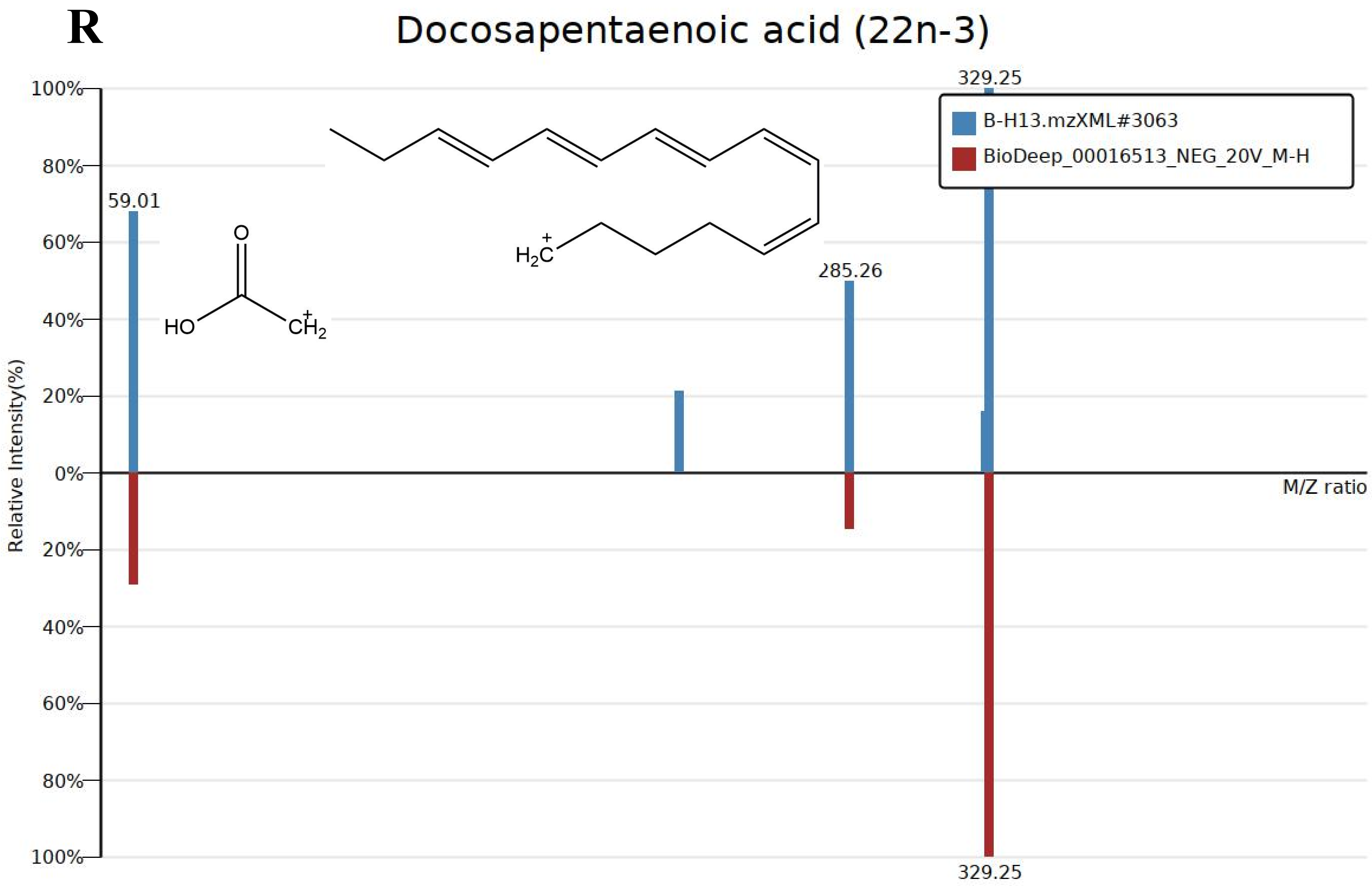
R
Docosapentaenoic acid (22n-3)
100%
80%
60%
40%
20%
0%
Relative Intensity(%)
59.01
HO
O
CH2
H2C
285.26
329.25
B-H13.mzXML#3063
BioDeep_00016513_NEG_20V_M-H
M/Z ratio

S

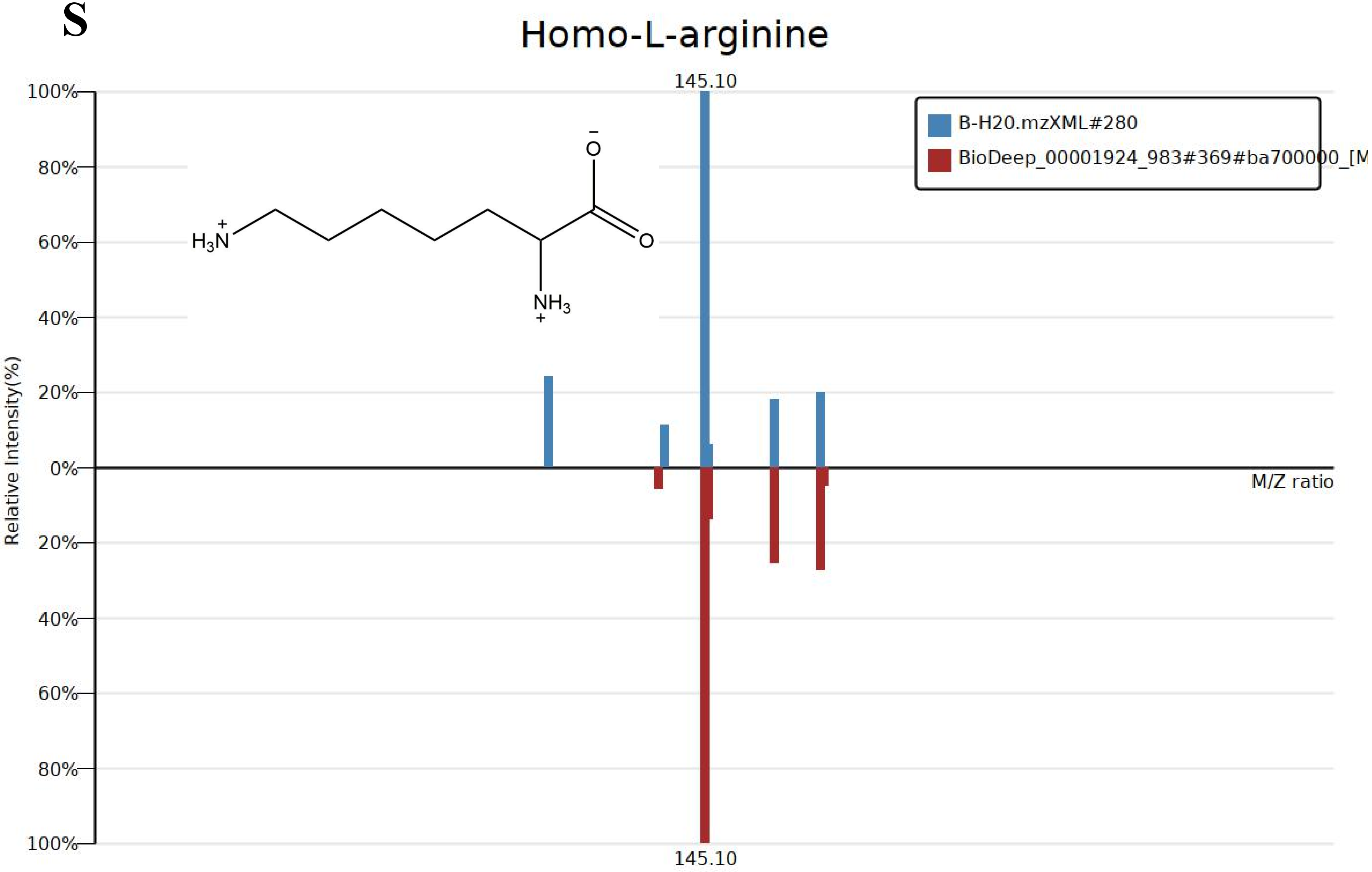

Homo-L-arginine
145.10
100%
80%
60%
40%
20%
0%
20%
40%
60%
80%
100%
Relative Intensity(%)
B-H20.mzXML#280
BioDeep_00001924_983#369#ba700000_[M
M/Z ratio
145.10

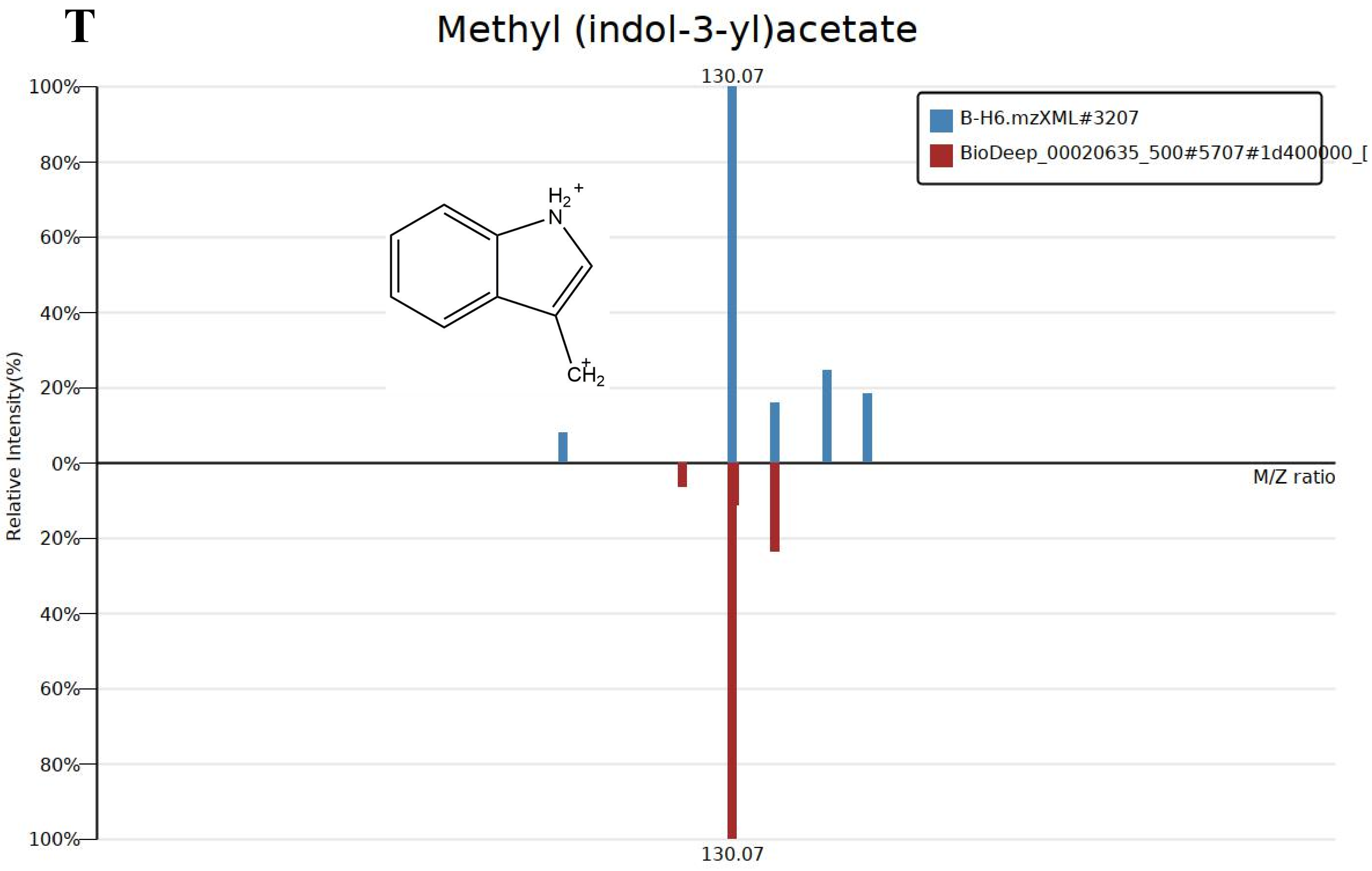
T
Methyl (indol-3-yl)acetate
130.07
100%
80%
60%
40%
20%
0%
20%
40%
60%
80%
100%
Relative Intensity(%)
B-H6.mzXML#3207
BioDeep_00020635_500#5707#1d400000_[
H_2^+
N
$\overset{+}{C}H_2$
M/Z ratio
130.07

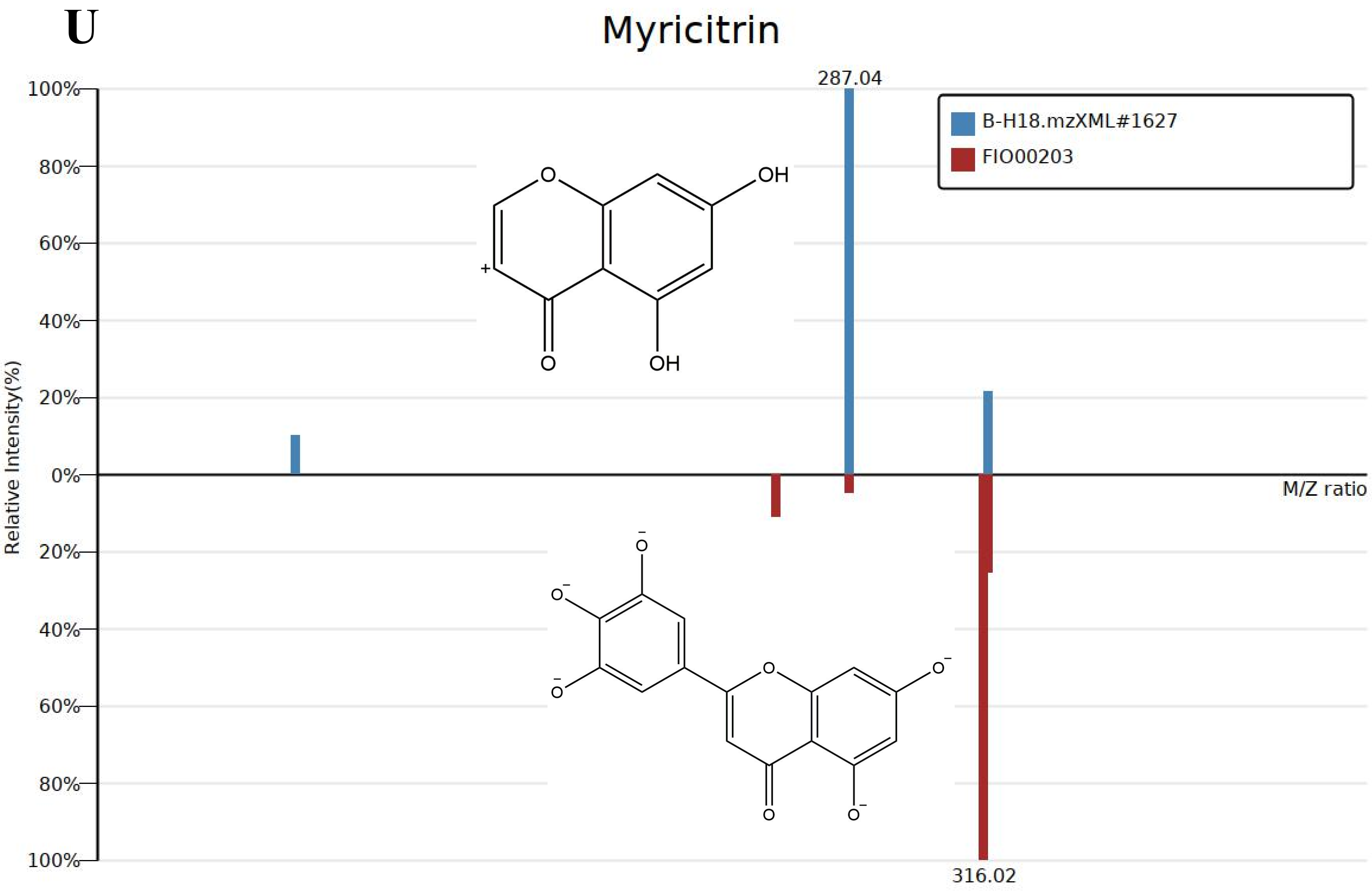
U
Myricitrin
287.04
B-H18.mzXML#1627
FIO00203
100%
80%
60%
40%
20%
0%
20%
40%
60%
80%
100%
Relative Intensity(%)
M/Z ratio
316.02
O
OH
OH
O
+
O
O
O
O
O
O
O
O
−
−
−
−
−

V

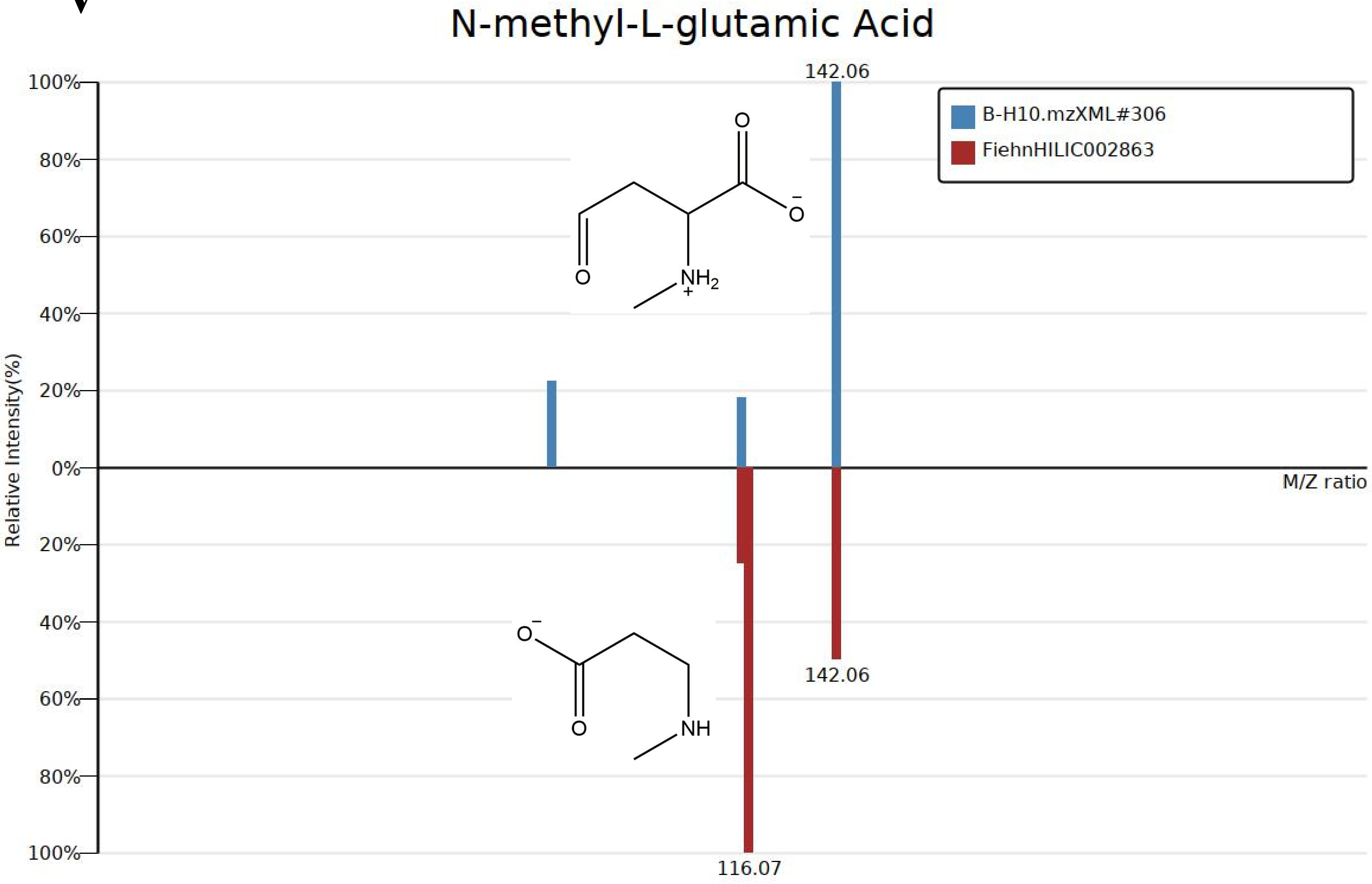
N-methyl-L-glutamic Acid
142.06
B-H10.mzXML#306
FiehnHILIC002863
100%
80%
60%
40%
20%
0%
Relative Intensity(%)
M/Z ratio
O
O
O
NH2
142.06
116.07
O
O
NH

W

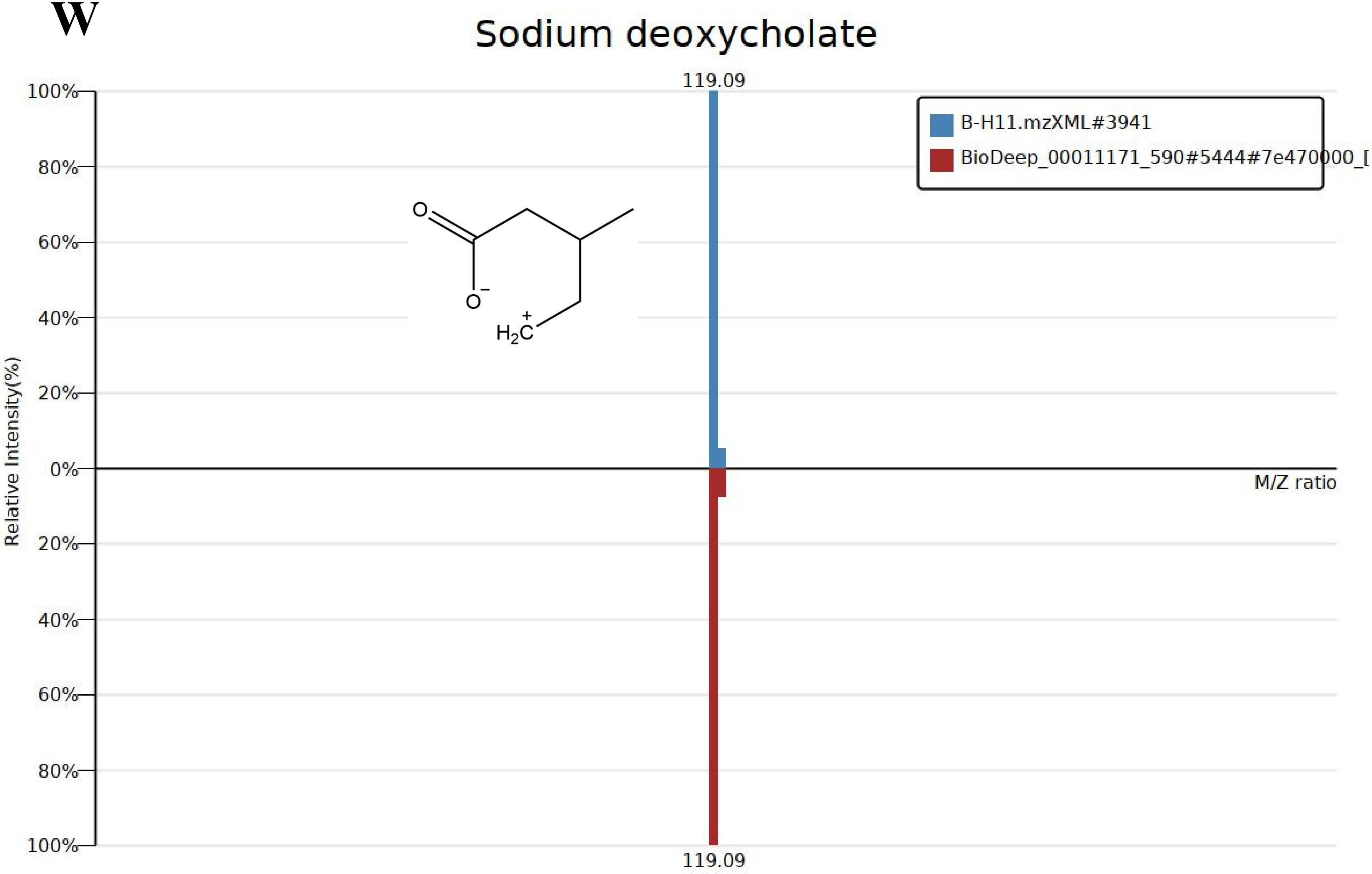

Sodium deoxycholate
119.09
100%
80%
60%
40%
20%
0%
20%
40%
60%
80%
100%
Relative Intensity(%)
B-H11.mzXML#3941
BioDeep_00011171_590#5444#7e470000_[
O
O
H2C
M/Z ratio
119.09

X

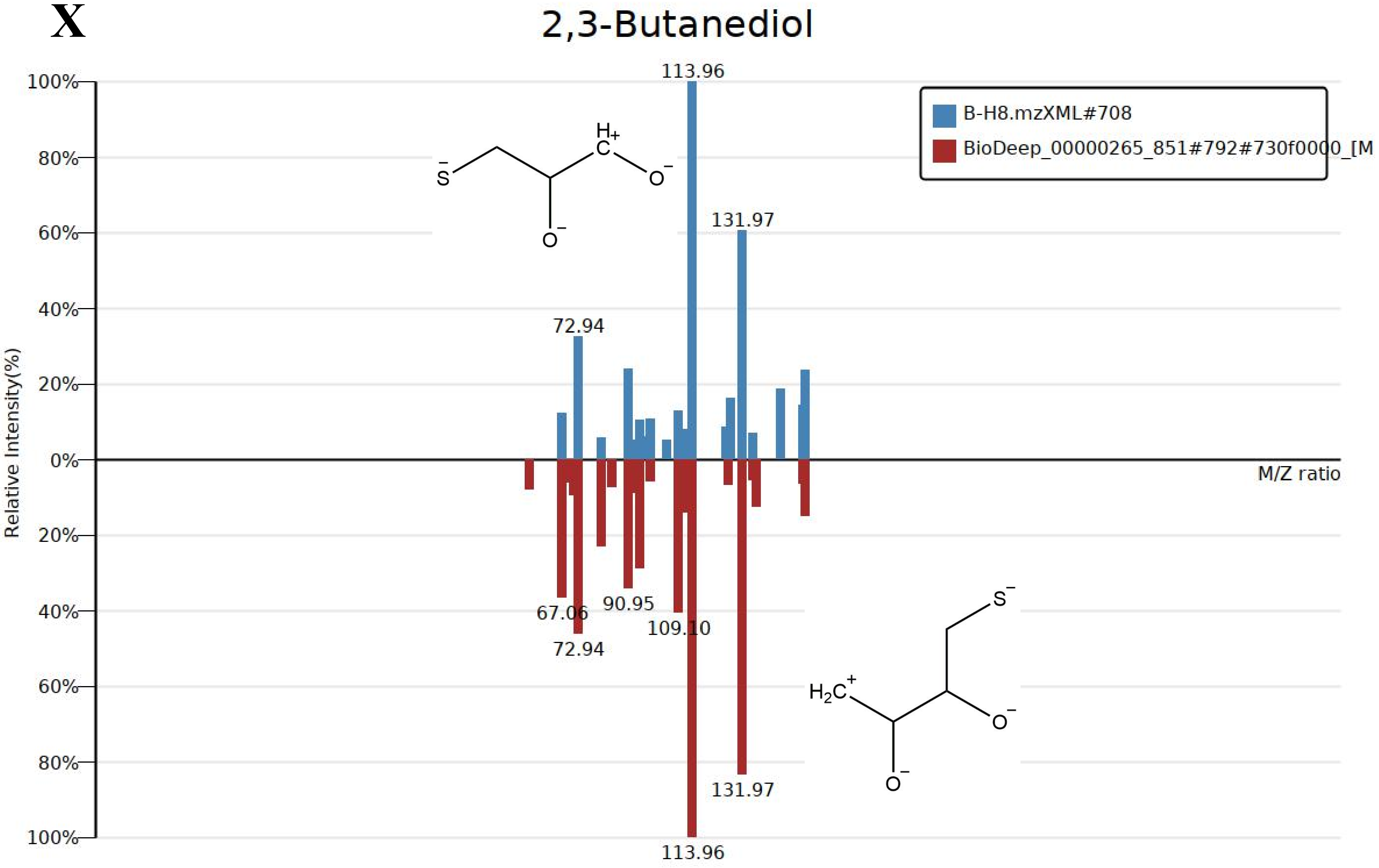

2,3-Butanediol
B-H8.mzXML#708
BioDeep_00000265_851#792#730f0000_[M
113.96
131.97
72.94
67.06
90.95
109.10
72.94
131.97
113.96
100%
80%
60%
40%
20%
0%
20%
40%
60%
80%
100%
Relative Intensity(%)
M/Z ratio

Y

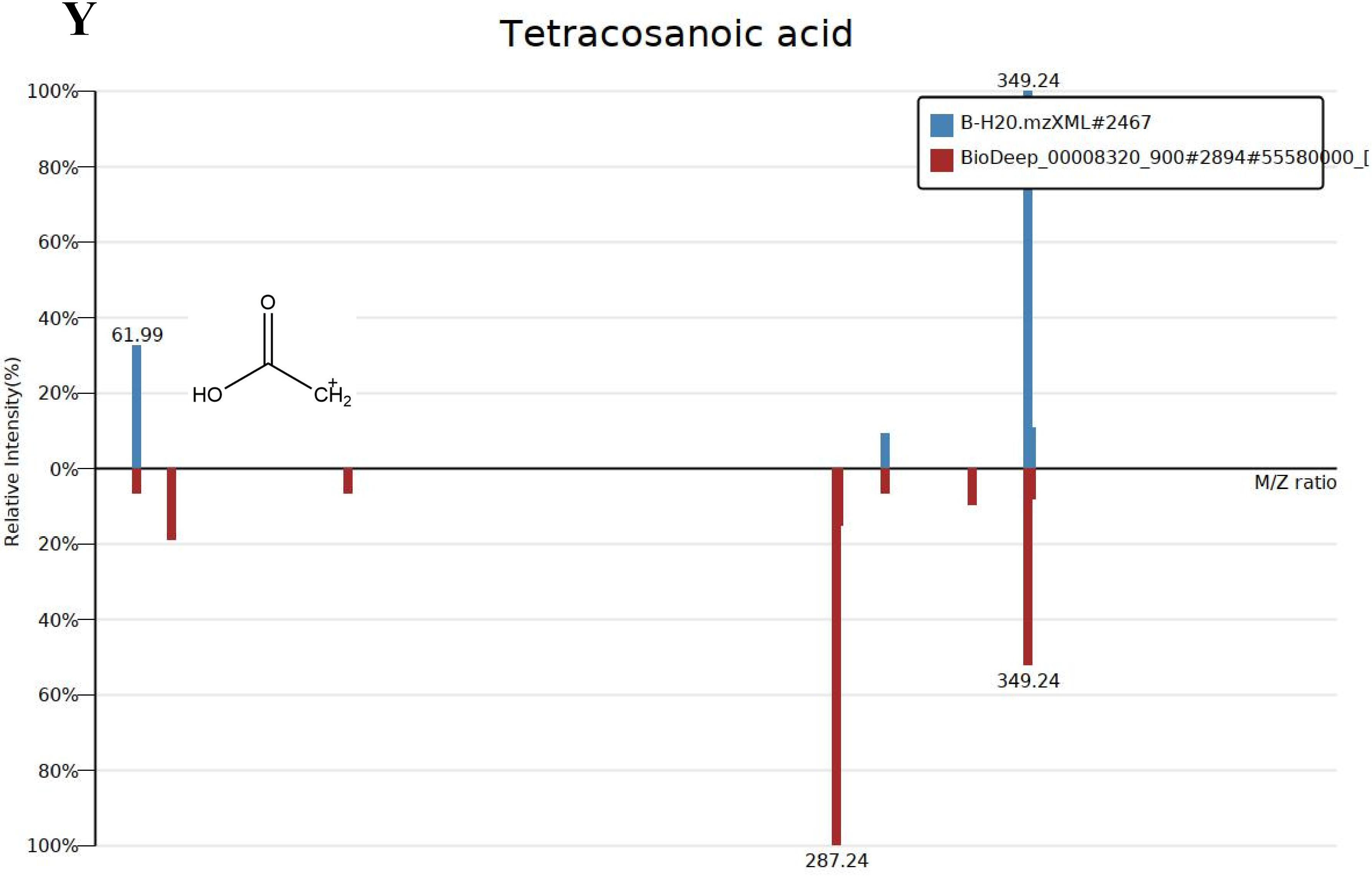
Tetracosanoic acid
349.24
B-H20.mzXML#2467
BioDeep_00008320_900#2894#55580000_[
100%
80%
60%
40%
20%
0%
20%
40%
60%
80%
100%
Relative Intensity(%)
61.99
O
HO
CH2
M/Z ratio
349.24
287.24

Z

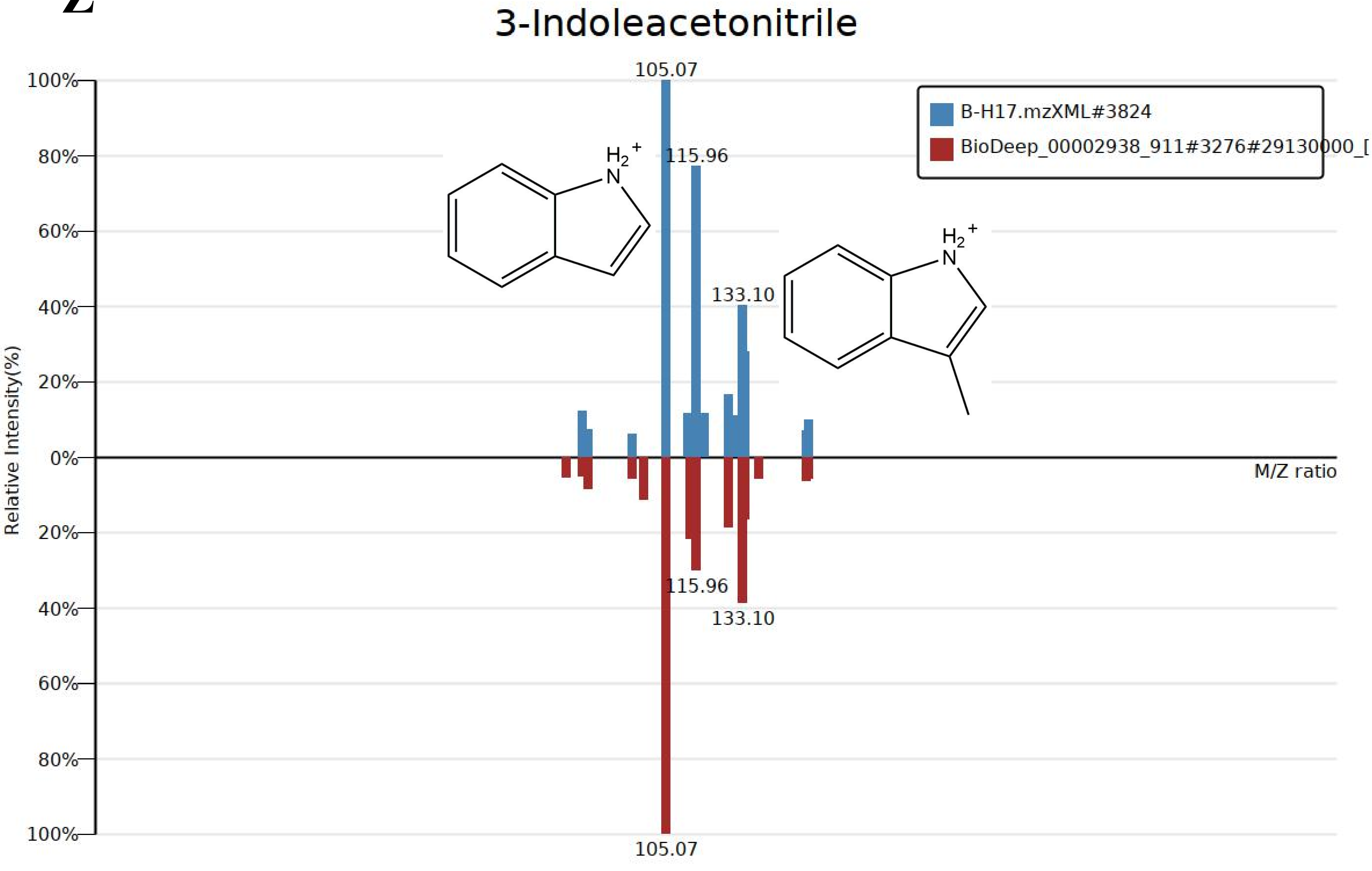
3-Indoleacetonitrile
B-H17.mzXML#3824
BioDeep_00002938_911#3276#29130000_[
105.07
115.96
133.10
Relative Intensity(%)
M/Z ratio
100%
80%
60%
40%
20%
0%
H_2^+
N
